# Strategic Synthesis of ‘Picket Fence’ Porphyrins Based on Nonplanar Macrocycles**

**DOI:** 10.1002/ejoc.202100154

**Published:** 2021-03-22

**Authors:** Karolis Norvaiša, Kathryn Yeow, Brendan Twamley, Marie Roucan, Mathias O. Senge

**Affiliations:** ^1^ Chair of Organic Chemistry School of Chemistry Trinity Biomedical Sciences Institute Trinity College Dublin The University of Dublin 152–160 Pearse Street Dublin 2 Ireland; ^2^ School of Chemistry Trinity College Dublin The University of Dublin Dublin 2 Ireland; ^3^ Institute for Advanced Study (TUM-IAS) Technical University of Munich Lichtenbergstrasse 2a 85748 Garching Germany

**Keywords:** Atropisomers, Curved π-systems, Macrocycles, Porphyrinoids, Supramolecular Chemistry

## Abstract

Traditional ‘picket fence’ porphyrin systems have been a topic of interest for their capacity to direct steric shielding effects selectively to one side of the macrocycle. Sterically overcrowded porphyrin systems that adopt macrocycle deformations have recently drawn attention for their applications in organocatalysis and sensing. Here we explore the combined benefits of nonplanar porphyrins and the old molecular design to bring new concepts to the playing field. The challenging *ortho*‐positions of meso‐phenyl residues in dodecasubstituted porphyrin systems led us to transition to less hindered *para‐* and *meta*‐sites and develop selective demethylation based on the steric interplay. Isolation of the symmetrical target compound [2,3,7,8,12,13,17,18‐octaethyl‐5,10,15,20‐tetrakis(3,5‐dipivaloyloxyphenyl)porphyrin] was investigated under two synthetic pathways. The obtained insight was used to isolate unsymmetrical [2,3,7,8,12,13,17,18‐octaethyl‐5,10,15,20‐tetrakis(2‐nitro‐5‐pivaloyloxyphenyl)porphyrin]. Upon separation of the atropisomers, a detailed single‐crystal X‐ray crystallographic analysis highlighted intrinsic intermolecular interactions. The nonplanarity of these systems in combination with ‘picket fence’ motifs provides an important feature in the design of supramolecular ensembles.

## Graphical abstract

**Nonplanar picket‐fence porphyrins**: Two sterically demanding systems, heavy beta substitution and pivalate groups, meet as an intrinsic supramolecular design on a porphyrin scaffold. The symmetrical, double‐sided, and unsymmetrical porphyrins were synthesized and analyzed by X‐ray crystallography, highlighting the combined benefits of steric interplay in designing supramolecular ensembles.

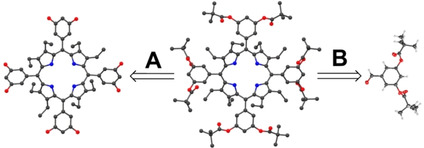

## Introduction

**Figure 1:**
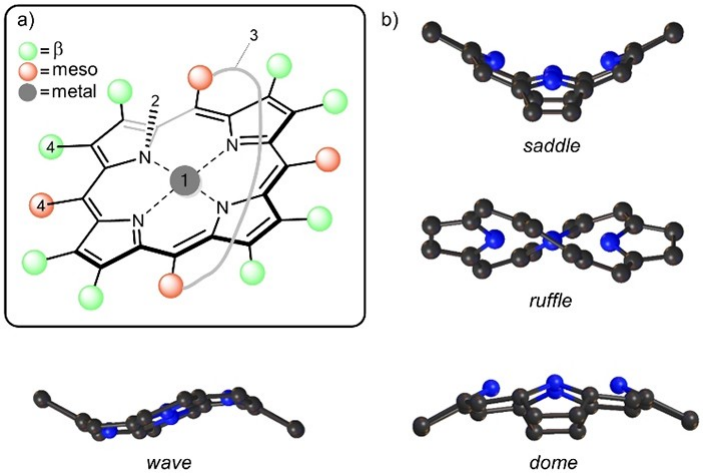
a) Methods of altering macrocycle conformation: (1) metalation; (2) *N*‐substitution; (3) covalent strapping of macrocycle; (4) sterically demanding peripheral substituents;^[5]^ b) Illustrations of the porphyrin macrocycle undergoing the saddle, ruffle, dome and wave out‐of‐plane distortions.^[7]^

The airbrushed image of a planar porphyrin^[1]^ seen in textbooks is not so commonly found in nature's catalog of tetrapyrrole‐containing proteins, where nonplanarity of the macrocycle is often evident. Heme proteins being the classical example, are a large class of metalloproteins with versatile roles from oxygen transport to electron transfer. After isolation the heme complex is planar; however, in biological systems, the porphyrin is bound to a protein scaffold which can induce distortion through covalent bonds and non‐covalent interactions.^[2]^ Reviews by Shelnutt *et al*.^[3]^ and us[^4^, ^5^] compile many examples of porphyrin‐containing proteins, highlighting how the scaffold exerts control over macrocycle planarity, affording its tunable physicochemical properties. For example, the very mechanism of heme biosynthesis requires the enzyme ferrochelatase to induce nonplanarity in order to insert the metal into the porphyrin core^[6a]^ while functional aspects of the photosynthetic reaction center are also controlled by specific chlorophyll conformations.^[6b]^ The now established concept of a porphyrin's conformational flexibility (Figure 1) has provided a much‐needed explanation as to why a single porphyrin cofactor possesses such diverse functionality.[^4^, ^5^]

Recently, much interest has been dedicated to nonplanar dodecasubstituted porphyrins as archetypical π‐curved heteroaromatic materials.^[5]^ The main means of their distortion is the overcrowding of the peripheral meso‐carbon and β‐pyrrole positions with substituents. As a result, substantial *peri*‐interactions arise, and steric strain must be relieved. The porphyrin ring distorts and adopts a nonplanar conformation, for dodecasubstituted porphyrins, this is typically the saddle distortion (Figure 1b), which provides the substituents with ample volume to comfortably occupy.

Dodecasubstituted porphyrins possess desirable properties that are unique to their nonplanar conformation.^[8]^ The accessibility of pyrrole N−H units in the inner core of the free base porphyrin enables hydrogen bonding while the exposed inner core lone pairs increase macrocycle basicity for applications in organocatalysis.^[9]^ Peripheral hydrogen bonding in cooperation with the cavities formed above and below the plane allow these molecules to arrange as supramolecular structures with nanochannels,^[10]^ or as functional sites for the binding and detection of small charged molecules.^[11]^ The full potential that sterically demanding, nonplanar porphyrin structures can offer is yet to be fully explored.

**Figure 2:**
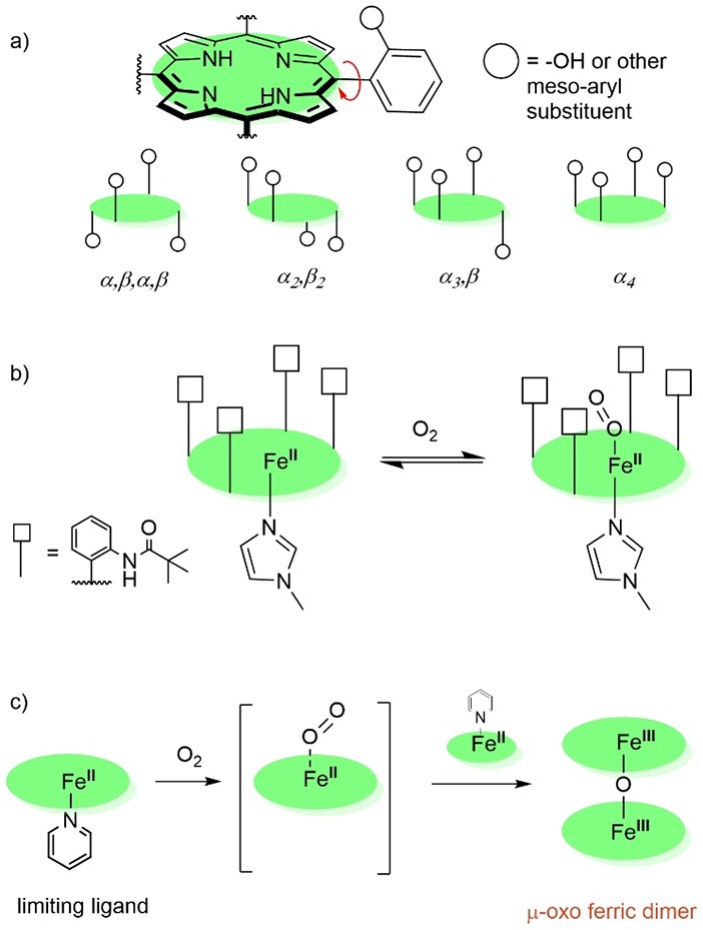
a) Possible porphyrin atropisomers based on meso‐substitution; b) The ‘picket fence’ concept: in *α_4_*‐orientation pivalic groups at meso positions forms oxygen‐binding pocket to the porphyrin ring while bulky base group disfavors coordination of base on ‘picket fence’ side and prevents oxygen coordination on the unhindered side;^[12]^ c) Without ‘picket fence’ shielding rapid autoxidation yields a μ‐oxo ferric dimer.

The existence of atropisomers amongst the meso‐arylporphyrins was first uncovered by Gottwald and Ullman.^[13]^ They successfully isolated the four individual atropisomers of 5,10,15,20‐tetrakis(*o*‐hydroxyphenyl)porphyrin, which were assigned according to the orientation of hydroxyphenyl‐substituents with respect to the porphyrin plane (Figure 2a). This biphenyl‐type atropisomerism arises from the restricted rotation of aryl groups around the porphyrin‐aryl C−C bond. At room temperature, separate atropisomers are usually stable but over time and at higher temperatures, the four atropisomers can re‐equilibrate to an expected statistical ratio of abundance (1 : 2 : 4 : 1). However, the rotational barrier to isomerization depends strongly on the steric bulk projecting from phenyl substituents.^[14]^ By applying a successful synthetic strategy, biphenyl‐type atropisomerism has since been exploited to prepare conformationally restricted systems that can be isolated.^[15]^

The term ‘picket fence’ in the field of porphyrin chemistry was first introduced by Collman *et al*. in the 1970s to describe the *α_4_*‐atropisomer of [5,10,15,20‐tetrakis(2‐pivalamidophenyl)porphyrinato]iron(II) or FeTpivPP (Figure 2b), which became a signature model for dioxygen binding to heme.^[12]^ The purpose of the bulky substituents was to form a protective pocket for the binding of dioxygen; earlier model complexes were susceptible to the irreversible iron autoxidation reaction which formed the μ‐oxo ferric dimer (Figure 2c) and gave only the illusion of reversible dioxygen binding.

Today, extensive studies on the active site of HbO_2_ and MbO_2_ have been carried out.^[16]^ These studies are made possible because of the protective cocoon‐like ‘picket fence’, which protects the subject ligand. Other coordination studies integral to life's signaling processes contain both a hydrogen bonding site and a sterically crowded component; as a result, suppressing or facilitating the binding of specific ligands.^[17]^ Moreover, the ‘picket fence’ scaffold has also been utilized for applications in novel chemical sensing^[18]^ and amperometric biosensing.^[19]^

The term ‘picket fence’ porphyrin has been broadened to include macrocycles with steric bulk extending from both sides of the plane, in other words, a ‘double‐sided’ or ‘bis‐picket fence’ porphyrin.^[20]^ meso‐Arylporphyrin systems with a (*o/m*) monosubstituted phenyl group exist as a mixture of four atropisomers. As such, the primary incentive to synthesize double‐sided porphyrins is that they possess a center of symmetry. The disubstituted phenyl at the *ortho*‐ or *meta*‐positions implies an atropisomer‐free product.^[21]^

The ‘picket fence’ substituents play an important role in creating a sterically constrained or selective environment, for hydrogen bonding or the ligation of small molecules. A synergistic situation could be realized when a porphyrin possesses both a picket fence and a distorted macrocycle. The cavities which exist are akin to the ‘lock and key’ model of molecular biology for enzyme active sites.^[8]^ The accessible inner core and N−H moieties of free base nonplanar porphyrins serve as potential substrate binding sites,^[22]^ and further intramolecular interactions between substrate and ‘picket fence’ can facilitate this. Hence, a combination of both nonplanar and ‘picket fence’ properties could have a cumulative effect in exerting more robust conformational control.

## Results and Discussion

### Target Compounds

**Figure 3:**
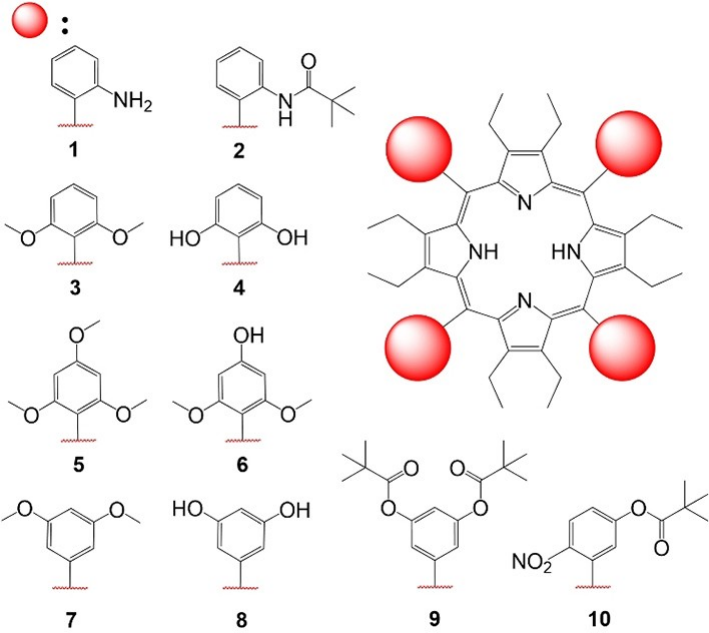
Library of synthesized nonplanar porphyrins for the corresponding project. Note, porphyrins **2** and **4** were not isolated as pure products.

With a refreshed view on the use of porphyrin macrocycles as receptors, their natural versatility, and diverse conformational landscape, in this project, we explore the possible sterically overcrowded porphyrin modifications through molecular engineering of bulky aryl substituents. We exploit challenging *ortho*‐ and *meta*‐ substitution patterns of the nonplanar porphyrin meso‐aryls to induce conformational changes. A variety of porphyrins were synthesized, presenting symmetrical (**3**–**7**) and unsymmetrical (**8**) meso‐substitutions with different levels of bulkiness to allow comparative conformational and structural analyses (Figure 3).

The 2,3,7,8,12,13,17,18‐octaethyl‐5,10,15,20‐tetraphenylporphyrin derivatives (**1**–**10**) were synthesized *via* modified Lindsey condensation reactions^[23]^ from the 3,4‐diethylpyrrole **11** and corresponding aldehydes (**3A**, **5A**, **7A**, **9A**, **10A**) in stoichiometric amounts.

### Synthesis and Challenges

First, following the ‘picket fence‘ porphyrin concept introduced by Collman *et al*. in planar porphyrin frameworks,^[12]^ a nonplanar precursor *α_4_*‐**1** was synthesized following known procedures.^[22]^

**Figure 4:**
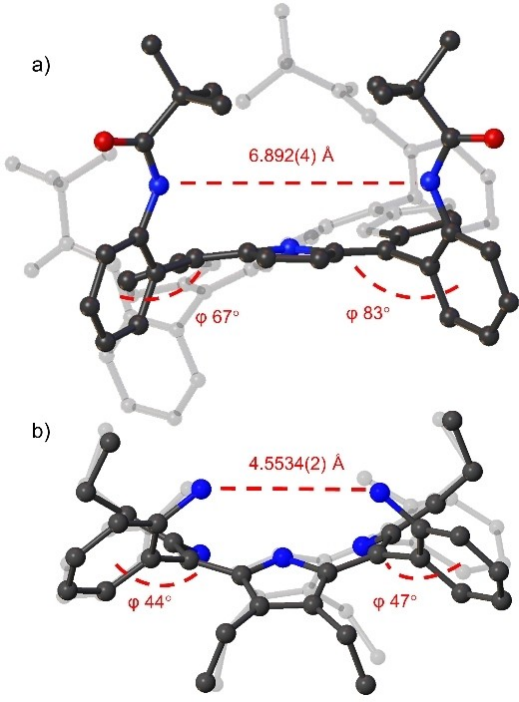
Structure comparison, the distance between the two closest peripheral nitrogen atoms as well as the degree of phenyl rotation to the mean‐plane of the 24 atom macrocycle: a) Structure of planar ‘picket fence’ porphyrin;^[25]^ b) Structure of nonplanar ‘picket fence’ precursor **1**.^[22]^

However, our attempt to introduce a ‘picket fence’ component using pivaloyl chloride resulted in incomplete substitution with the majority of *tri*substituted porphyrin derivative present in the reaction mixture after 24 hours (Figure S60). An additional 3 hours in boiling chloroform (60 °C) produced only trace amounts of *tetra*substituted porphyrin **2** (Figure S61). The challenging substitution of **1** on the *ortho*‐positions can be attributed to the proximity of amine groups (N…N∼4.55 Å) due to the ∼45° phenyl rotation (Figure 4). Additionally, bulky pivalate groups might induce atropisomerism to relieve the steric strain upon substitution. Therefore, to eliminate the possibility of atropisomerism, we targeted the double‐sided, octahydroxy system **8** as our new building block for further substitutions.

The ‘picket fence’ ester porphyrin synthesis provisionally reported on planar systems involves isolation of a methoxy porphyrin, with subsequent demethylation for further substitution.^[24]^ Following this, our so‐called conventional pathway, the corresponding nonplanar porphyrin **3** was synthesized in 70 % yield. Unfortunately, demethylation with BBr_3_⋅Et_2_O was not observed to full completion in various conditions (Table S2). Using another common demethylating agent, TMSI (trimethylsilyl iodide), no transformation was observed. The complete inactivity of TMSI could be ascribed to the radius of the coordinating atom (silicon atom being larger than boron). From the observations of complicated functionalization of **1** and unsuccessful demethylation of **3**, we concluded that *ortho‐*phenyl positions in dodecasubstituted porphyrins are sterically too hindered for potential substitution chemistry. With this in mind, less hindered *meta‐* and *para*‐phenyl sites were targeted for the demethylation procedures.

**Figure 5:**
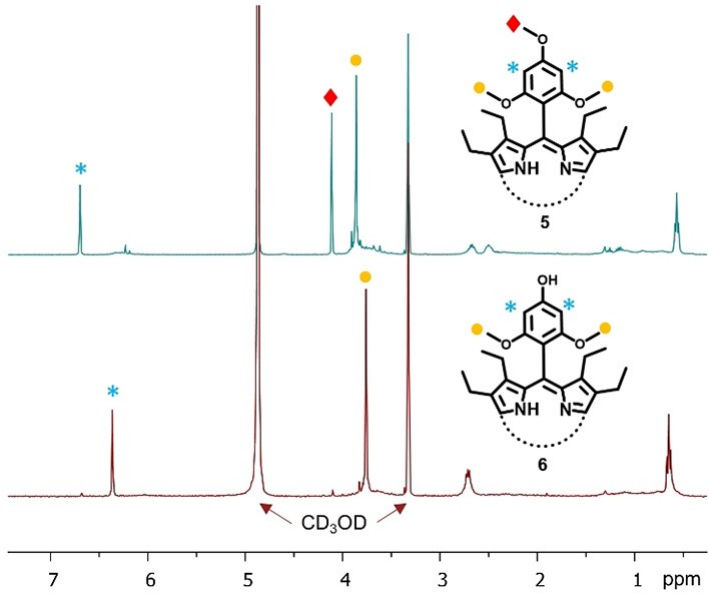
^1^H NMR spectra recorded in CD_3_OD of **5** and **6** highlighting the selective demethylation of *para*‐positions.

To investigate the accessibility and selectivity towards *para*‐sites, porphyrin **5** bearing twelve methoxy groups (eight in *ortho‐* and four in *para‐*positions) was synthesized in 10 % yield. The demethylation of **5** using 7 eq. BBr_3_⋅Et_2_O produced selectively porphyrin **6** possessing eight *ortho‐*methoxy and four *para‐*hydroxy groups in 86 % yield (Figure 5). The accessibility of *meta*‐positions was explored using porphyrin **7**. Upon isolation of octamethoxyporphyrin **7** in 19 %, the subsequent demethylation step of *meta*‐sites resulted in complete and quick conversion to octahydroxyporphyrin **8** in 76 % yield. The significant difference in demethylation attempts compared to that of **3** shows how sterically overcrowded the *ortho*‐positions become upon the introduction of β‐substituents. Demethylation of more accessible positions while sustaining the substitution on *ortho*‐sites produces an exciting supramolecular engineering technique for selective aryl modifications.

Later, pivaloyl chloride was used to provide the ‘picket fence’ feature to the dodecasubstituted hydroxyporphyin **8** to afford the desired ‘picket fence‘ porphyrin **9** in 67 %. However, only 10 % in overall yield was obtained mainly due to the relatively low‐yielding porphyrin condensation step (19 %), forming the ‘bottle‐neck’ in the synthetic pathway. Note, working with the highly polar hydroxyporphyrin **8** is synthetically tedious due to the limited solubility in aprotic solvents necessary for further functionalization.

**Scheme 1:**
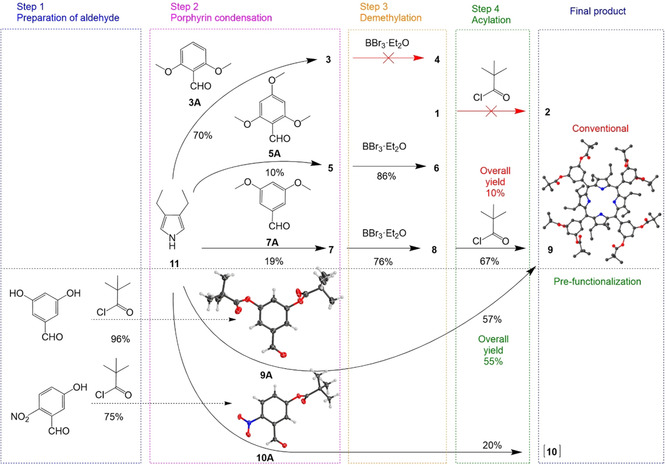
Synthetic pathways for the target compounds: Conventional route consisting of a 3 step synthesis including condensation, demethylation, and acylation steps; Alternatively, a pre‐functionalization pathway consisting of 2 steps to include the preparation of aldehyde and porphyrin condensation to yield the target compound; Aldehydes **9A**, **10A**, **8** and **9** were successfully re‐crystallized forming crystalline compounds that were later analyzed *via* X‐ray crystallography, non‐essential hydrogen atoms, solvent, and counter anion molecules omitted for clarity, thermal ellipsoids give 50 % probability.

To optimize the reaction, an alternative pathway for the synthesis of porphyrin **9** *via* pre‐functionalization of aldehyde **7A** was investigated (Scheme 1). The preparation of the aldehyde was carried out with pivaloyl chloride at 25 °C in MeCN and the presence of *N,N‐*diisopropylethylamine (DIPEA) and achieved full conversion to pivalate ester aldehyde **9A** in 96 % yield after 1 h. After the successful preparation of **9A**, further condensation with **11** gave the target porphyrin **9** in 57 % yield. Two key observations arise: firstly, the pre‐functionalization pathway is one step shorter in comparison to the conventional synthesis; and secondly, the five‐fold increase in the overall yield, 55 % by pre‐functionalization compared to 10 % *via* the conventional pathway, overcomes the formation of the ‘bottle‐neck’ in the reaction.

To explore unsymmetrical systems we chose the established pre‐functionalization pathway to obtain target compound **10**. Thus, aldehyde **10A** was prepared in 75 % yield from 5‐hydroxy‐2‐nitrobenzaldehyde and pivaloyl chloride in the presence of DIPEA (Scheme 1). The corresponding condensation reaction afforded unsymmetrical picket fence porphyrin **10** in 20 % yield as an atropisomeric mixture. Note, mass spectrometry indicated the formation of the desired macrocycle; however, it was also observed that a few of the pivalate ester groups were susceptible to cleavage in the presence of the Lewis acid BF_3_. Therefore, upon work‐up, additional four equivalents of pivaloyl chloride were used in order to isolate **10**. The weakening of the pivalate ester group could have been influenced by the highly electron‐withdrawing NO_2_ substituents on the phenyl moiety, as the previous double‐sided system **9** showed no loss of the corresponding groups. The pre‐functionalization of aldehydes is a versatile and efficient way to directly obtain nonplanar porphyrin target compounds and may be utilized in a wider scope of porphyrin systems in the future.

**Scheme 2:**
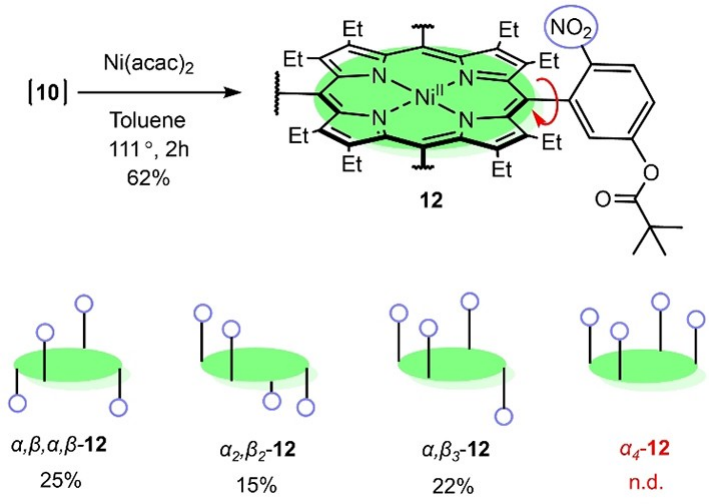
Synthesis of **12** and isolated corresponding atropisomers. In blue, each *meso*‐phenyl presents a single NO_2_ group that can be positioned either above or below the plane.

We established earlier that to provide stability for individual atropisomers during purification, the free base nonplanar porphyrin can be converted to the nickel(II) derivative.^[22]^ Therefore, an atropisomeric mixture of **10** in toluene was stirred at 111 °C with five equivalents of nickel(II) acetylacetonate for 2 h to afford a 65 % yield of **12** (Scheme 2). Thin‐layer chromatography (SiO_2_, CH_2_Cl_2_) indicated an excellent separation of three components, which were confirmed by X‐ray crystallography to be three atropisomers *α,β,α,β‐* **12**, *α_2_β_2_‐* **12**, and *α_3_β* **‐12** (Figure 7).

Typically, the unsymmetrical spatial moieties produced in a mixture of four possible atropisomers are in a statistical ratio of 1 : 2 : 4 : 1 (Figure 2a).[^12^, ^13^, ^22^] However, due to the introduction of the bulky *meta*‐position substituents, the *α,β,α,β‐* **12** rotamer was obtained in high yield, while *α_4_* **‐12** was not detected. A similar concept of introducing bulky substituents to *meta‐*positions has been provisionally reported by Arai *et al*. in planar porphyrins.^[14]^ As such, quantities of the purified atropisomers *α,β,α,β‐* **12**, *α_2_β_2_‐* **12** and *α_3_β*‐**12** were afforded in the abundance ratio of 1.7 : 1 : 1.5 respectively, opposed to the statistical ratio of 1 : 2 : 4 (Scheme 2).

The deviation from the statistically expected ratio indicated that there could be factors driving the selection of particular atropisomers. We expect that the alternating *α,β,α,β‐* substituents above and below the plane have the lowest energy and least sterically strained conformation, while *α_4_* **‐12** has the highest. The driving force for selection of this *α,β,α*,*β*‐conformation leads to the lower yield of the other atropisomers in the mixture. Upon ^1^H NMR thermal evaluation of the individual atropisomers, we have determined the stability to correspond following *α,β,α,β‐* **12**>*α_2_β_2_‐* **12**>*α_3_β*‐**12** (see Figure S62 for more details). Note, individual atropisomers were isolated upon nickel(II) insertion at 111 °C for 2 hours. Moreover, the thermal stability experiment showed that all the rotamers are prompt for changing the composition. Hence, convergence reaction to one atropisomer by heating is unlikely.^[26]^ However, the kinetic formation ratio, thermal equilibration, and enhancement of the individual **12** atropisomers call for a separate, sophisticated physicochemical project in the future.

**Figure 6:**
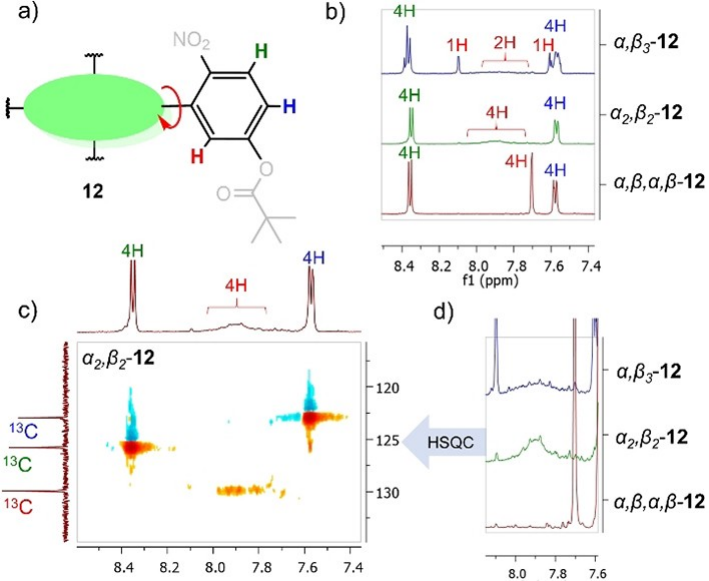
Characteristic ^1^H NMR signals of **12** individual isolated atropisomers recorded in CDCl_3_; a) illustration of aromatic proton signals: red (*o*‐Ar*H*), blue (*p*‐Ar*H*), green (*m*‐Ar*H*); b) Overlay spectra of **12** atropisomers aromatic regions; c) ^1^H‐^13^C HSQC of *α_2_β_2_‐* **12**; d) expansion of the **12** *o*‐Ar*H* region.

**Figure 7:**
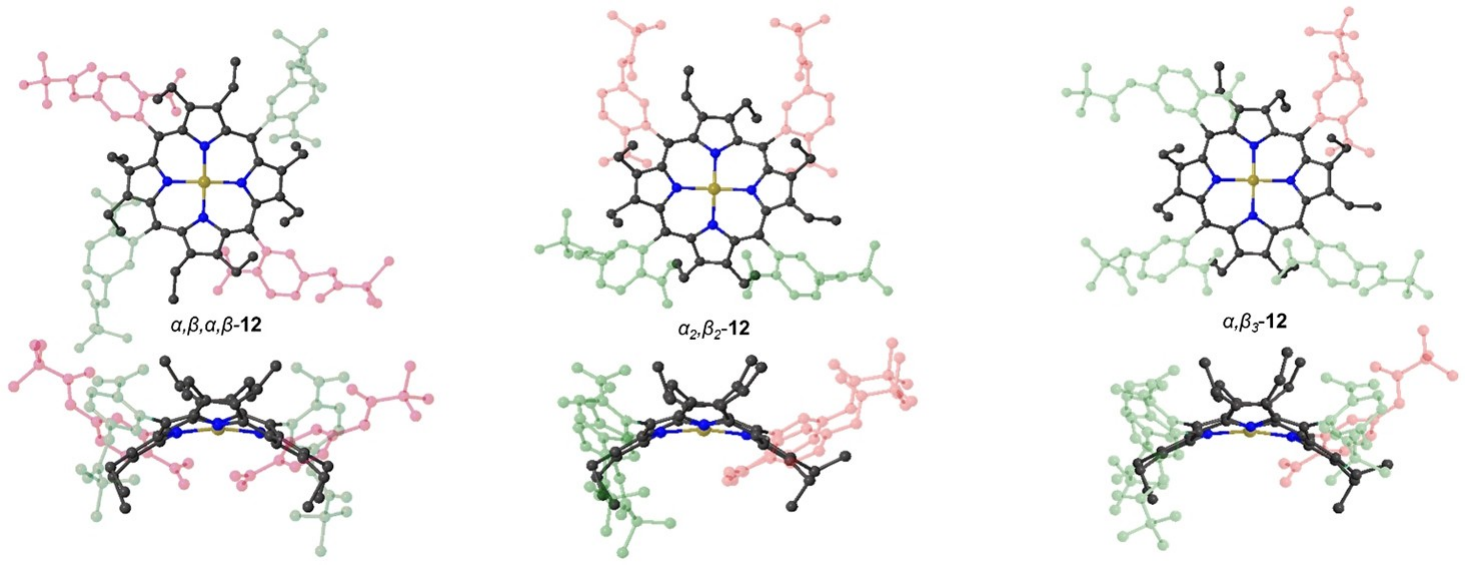
Molecular structures of the isolated *α,β,α,β‐* **12**, *α_2_β_2_‐* **12** and *α_3_β* **‐12** atropismers. Highlighted in green are the aromatic units of which NO_2_ motifs are pointing above the macrocycle plane, whereas in red, NO_2_ motifs are pointing below the plane. Hydrogen atoms and solvent molecules omitted for clarity, thermal ellipsoids give 50 % probability.

The ^1^H NMR of **12** shows distinctive aromatic region signals explicit to the isolated individual atropisomers (Figure 6). The *α_3_β*‐**12** has a plentitude of aromatic signals due to its highly unsymmetrical nature (C_*1*_), the symmetrical *α,β,α,β‐* **12** (S_*4*_) and *α_2_β_2_‐* **12** (C_*2v*_) presents *p*‐Ar*H* and *m*‐Ar*H* as well defined doublets (Figure 6b).^[27]^ While *p*‐Ar*H* and *m*‐Ar*H* signals are indistinguishable between atropisomers, the *o*‐Ar*H* have very distinct features (Figure 6d): sharp singlet in *α,β,α,β‐* **12**, broad singlet in *α_2_β_2_‐* **12**, and a broad singlet with two sharp singlets in *α_3_β*‐**12**. The broadening of the *o*‐Ar*H* in *α_2_β_2_‐* **12** could be attributed to the tendency of intermolecular interactions, forming interlocked system by the nitro groups as opposed to the *α,β,α,β‐* **12**. An in‐depth discussion on the repulsion, spatial arrangements, and intermolecular interactions of the individual **12** atropisomers is given in the single‐crystal X‐ray crystallography section. It is worth noting that the solubility of *α_2_β_2_‐* **12** in DMSO is much greater than *α,β,α,β‐* **12** suggesting possible alterations of physicochemical properties upon the supramolecular assemblies (Figure 7).

The separated atropisomers **12** have three primary features which aid further long‐term projects. Installing coordinating groups at the macrocycle periphery is of great interest as a means of introducing bifunctionality to free base porphyrins which already possess exposed N−H units.[^9a^, ^9d^] Additionally, the unsymmetrical nature of *α_3_β* **‐12** brings about the existence of a chiral atropisomer^[27]^ and the added benefit of the picket fence moieties is the potential to increase the rotational barrier to reduce the re‐equilibration of atropisomers.^[14]^

### Single Crystal X‐ray Crystallographic Analysis

**Figure 8:**
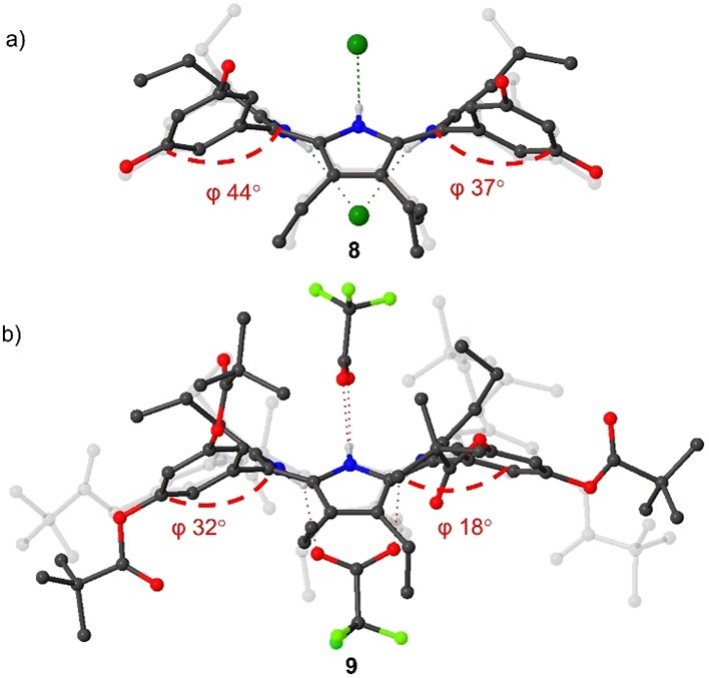
Comparison of the structure a) **8** and b) **9** of rotation degrees in phenyl moieties to the mean‐plane of 24‐atom macrocycle. Non‐essential hydrogen atoms and solvent molecules omitted for clarity, thermal ellipsoids give 50 % probability.

The structures of aldehydes **9A**, **10A**, and porphyrins **8**, **9**, *α,β,α,β‐* **12**, *α_2_β_2_‐* **12**, and *α_3_β*‐**12** were confirmed by single‐crystal X‐ray analysis (Figure S59). Aldehyde **9A** crystallized in the monoclinic lattice system with a *C2/c* space group while **10A** occupied a triclinic lattice with a *P* $\bar{1}$ space group. Porphyrins **8** and **9** were recrystallized with corresponding acids: **8** with HCl and **9** with TFA (trifluoracetic acid). As expected, in **8** the phenyl rotation to the macrocycle plane was found to be ∼40° (Figure 8a). However, the phenyl rotation was observed almost 2‐fold smaller in the structure of **9** (Figure 8b) due to the introduction of pivalate groups. In the unsymmetrical **12** systems, the phenyl rotation was found to be unaffected by the pivalate esters. This highlights the sterically demanding nature of symmetrical octa‐pivalate ester porphyrin **9**.

**Figure 9:**
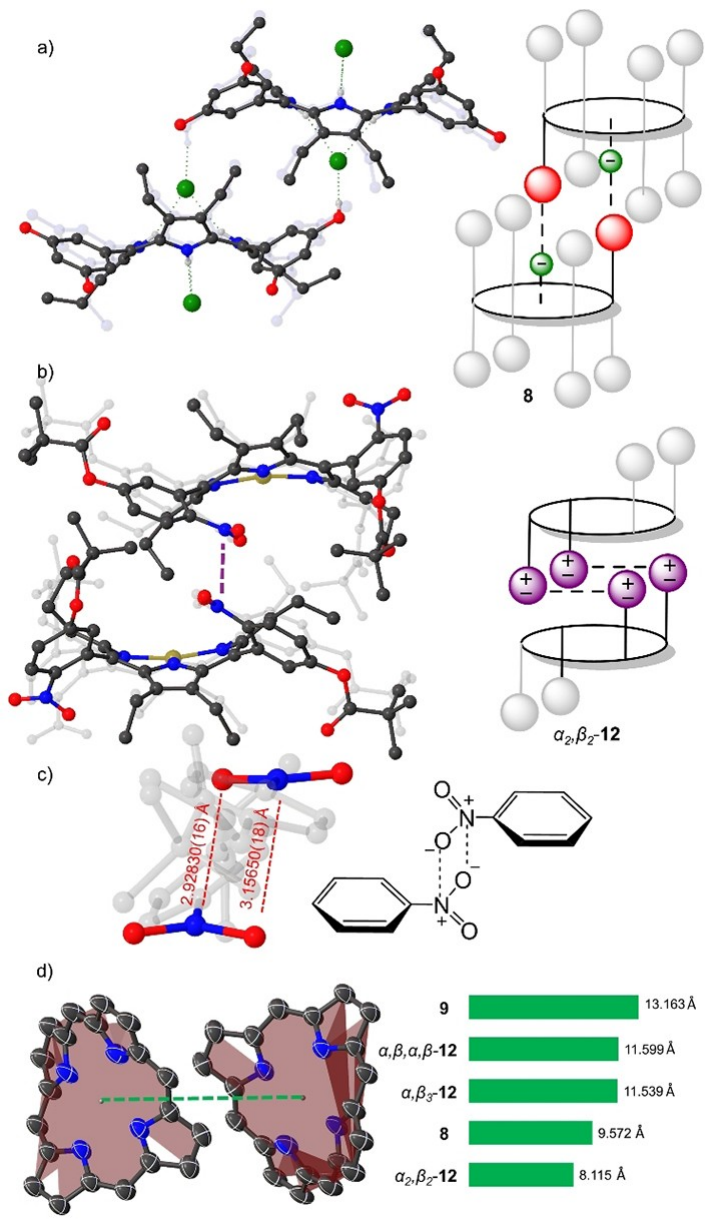
a) Representation of head‐to‐tail interactions observed in **8**; b) Interlocked *α_2_β_2_‐* **12** system by NO_2_ groups; c) N⋅⋅⋅O interactions and corresponding distances observed in *α_2_β_2_‐* **12** structure; d) Visualization of the distances between the centroids of two closest 24‐atom macrocycle planes in the obtained porphyrin structures. Hydrogen atoms and solvent molecules omitted for clarity; thermal ellipsoids give 50 % probability.

In structure **8**, a large hydrogen‐bonding network was observed due to eight peripheral donor sites (O−H) and the inner core system (N−H) as opposed to structure **9** in which all of the peripheral donor sites are substituted by pivalate esters. Both of the structures exhibit linear or “I‐shaped” complexation patterns^[27]^ where two of the counter anions are coordinated symmetrically on two opposing sides of the macrocycle (Figure 8). Additionally, head‐to‐tail^[10d]^ intermolecular interactions were observed between two of the porphyrin **8** units (Figure 9a). The hydrogen‐bonded Cl anion serves as a bridge, coordinating to the inner core system of one porphyrin and hydrogen bonding to one of the peripheral donor sites (O−H) in the other porphyrin unit.

The structure *α_2_β_2_‐* **12** exhibits multiple weak intermolecular N⋅⋅⋅O interactions of NO_2_ groups, interlocking the two of the porphyrin units (Figure 9b). Following the criteria adopted by Woźniak *et al*.^[28]^ N⋅⋅⋅O interactions are judged to be significant if the distance is less than 3.5 Å, therefore, *α_2_β_2_‐* **12** satisfy this criterion by lying in the 2.92830(16) Å–3.15650(18) Å range (Figure 9c). Other nitro group bearing structures *α,β,α,β‐* **12**, *α_3_β*‐**12**, and **10A** did not show any of the N⋅⋅⋅O interactions. The selective N⋅⋅⋅O interlocked system in *α_2_β_2_‐* **12** can be attributed to the perfect fit by positioning of the NO_2_ groups and pivalate group. While NO_2_ groups in the *α,β,α,β‐* **12**, and *α_3_β*‐**12** structures could form weak interactions, the repelling forces of pivalate groups introduced to the opposing sides eliminate this possibility.

The intermolecular interactions unambiguously affect the packing of the systems. The distance between the centroids of the two closest 24‐atom macrocycle planes are: *α_2_β_2_‐* **12**<**8**<*α_3_β*‐**12**<*α,β,α,β‐* **12**<**9** (Figure 9d). The weak N⋅⋅⋅O interactions in *α_2_β_2_‐* **12** and Cl⋅⋅⋅H in **8** which help to hold two of the corresponding units nearby can be considered to be the principal force ensuring adhesion of the layers, while the pivalate groups act as repelling units.

**Figure 10:**
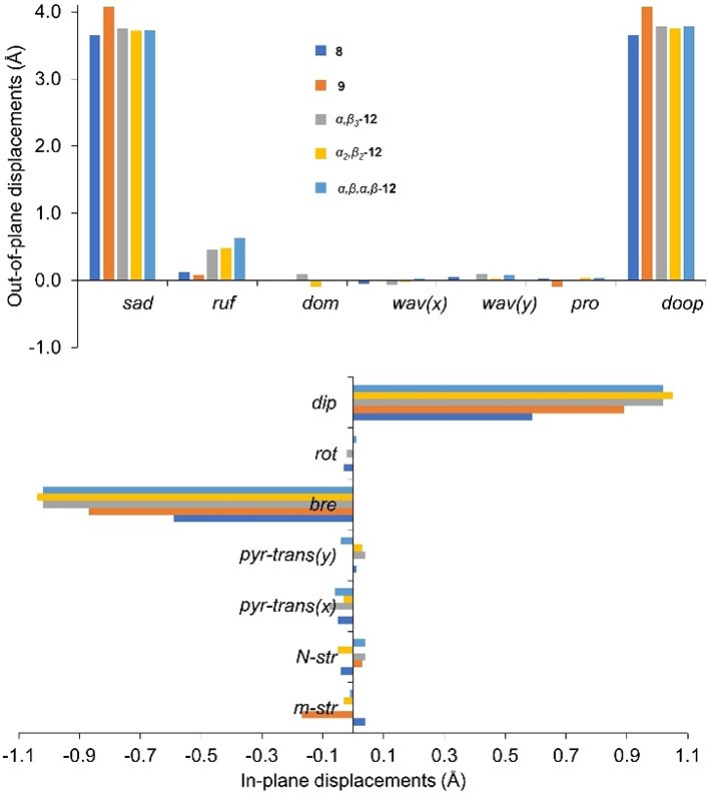
Illustration of the out‐of‐plane and in‐plane normal‐coordinate structural decomposition results for **8**, **9**, *α,β,α,β‐* **12**, *α_2_β_2_‐* **12**, and *α_3_β*‐**12** structures.

The plentitude of chemical modifications of the conformational flexible macrocycles produced a variety of altered tetrapyrroles.^[5]^ Shelnutt and co‐workers introduced NSD (normal‐coordinate structural decomposition) as an easy method to delineate, quantify, and illustrate the various distortion modes present in the porphyrin macrocycle.^[7]^ Therefore, to compare the conformational distortion of isolated crystal structures, the NSD analysis for out‐of‐plane (*oop*) and in‐plane (*ip*) distortions was performed (Figure 10). The structures displayed high levels of saddle (*sad*) distortion with minimal difference in total out‐of‐plane distortions (*doop*). The lowest out‐of‐(24‐atom)‐plane alterations were observed for **6**, while the highest value of *doop* was detected in **7**. The increase of *sad* in **7** can be contributed to the angle of which phenyl groups are rotated, forcing the pyrrole units to tilt even further *via* peri interactions (Figure 8b). The increase of the *ruf* distortion in the atropisomers of dodecasubstituted porphyrins by coordination of bulky counter anions has previously been shown.^[27]^ A significant increase of ruffling in all **12** atropisomers (*α_3_β*‐**12**<*α_2_β_2_‐* **12**<*α,β,α,β‐* **12**) introduces a new asset of controlling the ruffling profile by peripheral manipulations. The structures are comparable to that of the classic example of highly *ruf* and *sad* structure of 5,10,15,20‐tetrakis(*tert*‐butyl)porphyrindi‐ium bis(trifluoroacetate) [H_4_TtBP][CF_3_COO^−^]_2_ ⋅ 2CF_3_COOH.^[29]^

Regarding in‐plane distortions, strong breath (*bre*) deformations were observed in all isolated porphyrin structures. The highest *bre* observed in **12** atropisomers signifies the porphyrin macrocycle contraction by nickel(II) coordination. The decrease in *bre* and meso‐stretch (*m‐str*) of **9** in comparison to that of **8** can be attributed to the binary coordination of the counter anion (TFA) as opposed to the singular coordination by Cl (Figure 8). Overall, X‐ray crystallographic analysis proved to be a crucial asset in the assessment of the intermolecular interactions and flexibility of the macrocycles that are essential for supramolecular engineering.

## Conclusion

In this article, a series of pre‐functionalized aldehydes and nonplanar picket fence porphyrins were successfully prepared. It was found that *ortho*‐positions of dodecasubstituted porphyrins are sterically very demanding. The demethylation of more accessible *meta‐* and *para‐*sites over the *ortho*‐positions led to the development of a means to selectively introduce functionality into the systems. Moreover, two different synthetic pathways were investigated and it was determined that synthesis by pre‐functionalization of the aldehyde prior to condensation is the more efficient route. The concept of pre‐functionalization applied in the synthesis of unsymmetrical picket fence porphyrin **12** yielded three atropisomers isolated as Ni(II) derivatives. The successful separation and characterization of individual atropisomers can be attributed to the stability gained by the steric bulk of the picket fence moiety, as well as the stabilizing effect provided by Ni(II). The successful synthesis of a novel bis‐facial picket fence porphyrin **9** heralds the development of a new class of molecular building blocks in functional porphyrin synthesis, with attractive and accessible substrate binding sites for future in‐depth receptor studies.

The single‐crystal X‐ray crystallographic analysis of isolated structures showed the formation of weak intermolecular N⋅⋅⋅O in *α_2_β_2_‐* **12** and Cl⋅⋅⋅H in **8** bridging two of the porphyrin units in close proximity, while **9** with eight pivalate groups provided a repulsion between the corresponding units. This prearrangement of porphyrins has implications in the design of molecular sponges incorporating porphyrin components,^[10]^ as well as photophysical properties where control of intermolecular distances is important.^[30]^ A detailed NSD analysis was carried out showing higher *sad* values in octa‐pivalate ester porphyrin **9** as opposed to the other structures. An increase in saddling could be used in the preparation of a new generation of porphyrin‐based catalysts,^[9]^ since a gradual increase in *sad* distortion can drastically influence performance in organocatalysis.^[9d]^ Moreover, as *sad* distortion affects the basicity of porphyrin systems,^[31]^ the conformational control aspects could be used in the development of higher sensitivity porphyrin‐based sensors for the detection of charged analytes.^[22]^

## Experimental Section

**General Information**: Condensation reactions were carried out under an argon atmosphere. Reactions involving moisture and/or air‐sensitive reagents were carried out in pre‐dried glassware and with standard Schlenk line techniques. 3,4‐Diethyl‐1H‐pyrrole **11** ^[23]^ and [2,3,7,8,12,13,17,18‐octaethyl‐5,10,15,20‐tetrakis(2‐aminophenyl)porphyrin] **1** ^[22]^ were prepared *via* known procedures. All commercial reagents and anhydrous solvents were used as received from vendors (Sigma Aldrich, Acros Organics, Fluka, Frontier Scientific, and Fischer). Dichloromethane (CH_2_Cl_2_) was dried over P_2_O_5_. Yields refer to chromatographically and spectroscopically (^1^H NMR) homogeneous material unless otherwise noted. Reactions were monitored by thin‐layer chromatography (TLC) and absorption spectroscopy. Analytical thin‐layer chromatography was performed using silica gel 60 (fluorescence indicator F254, precoated sheets, 0.2 mm thick, 20 cm×20 cm; Merck) or aluminum oxide 60 (neutral, F254; Merck) plates and visualized by UV irradiation (*λ*=254 nm). Silica gel 60 *Merck, 230–400 mesh or aluminum oxide (neutral, activated with 6 % H_2_O, Brockman Grade III) was used for flash column chromatography. Mobile phases are given as (*v/v*). Room temperature refers to 20–25 °C.

**Instrumentation**: Melting points are uncorrected and were measured with a Digital Stuart SPM 10 melting point apparatus. NMR spectra were recorded on a Bruker Advance III 400 MHz, a Bruker Advance HD 400, and an Agilent 400 spectrometer for ^1^H (400.13 MHz) and ^13^C (100.61 MHz) NMR spectra. A Bruker Ultrashield 600 spectrometer was employed for ^1^H (600.13 MHz) and ^13^C (150.90 MHz) NMR spectra. All NMR experiments were performed at 25 °C. Resonances *δ* are given in ppm units and referenced to the deuterium peak in the NMR solvents, *d_4_*‐methanol (*δ*_H_=4.87, 3.31 ppm, *δ*_C_=49.1 ppm). CDCl_3_ (*δ*_H_=7.26 ppm, *δ*_C_=77.2 ppm). Signal multiplicities are abbreviated as follows: singlet=s, doublet=d, triplet=t, multiplet=m. Mass spectrometry was performed with a Q‐TOF Premier Waters MALDI quadrupole time‐of‐flight (Q‐TOF) mass spectrometer equipped with Z‐spray electrospray ionization (ESI) and matrix‐assisted laser desorption ionization (MALDI) sources in positive mode with *trans*‐2‐[3‐(4‐*tert*‐butylphenyl)‐2‐methyl‐2‐propenylidene]malononitrile (DCTB) as the matrix or using a Bruker Daltonics autoFlex system. ESI mass spectra were acquired in positive modes as required, using a Micromass time‐of‐flight mass spectrometer (TOF) interfaced to a Waters 2960 HPLC or a Bruker microTOF‐Q III spectrometer interfaced to a Dionex UltiMate 3000 LC. Atmospheric pressure chemical ionization (APCI) experiments were performed on a Bruker microTOF‐Q III spectrometer interfaced to a Dionex UltiMate 3000 LC. UV‐Vis spectra were recorded in solutions using a Specord 250 spectrophotometer from Quartz Glass 10 mm 6030‐UV (1 cm path length quartz cell). IR measurements were done on a PerkinElmer Spectrum 100 FT‐IR.

**Single Crystal X‐ray Crystallography**: Crystals were grown following the protocol developed by Hope, liquid‐liquid diffusion in CHCl_3,_ and methanol or oversaturated solutions in DMSO.^[32]^ Diffraction data for compounds were collected on a Bruker D8 Quest ECO and Bruker APEX 2 DUO CCD diffractometer using graphite‐monochromated Mo‐*K*_*α*_ (*λ*=0.71073 Å) or Incoatec IμS Cu‐*K*_*α*_ (*λ*=1.54178 Å) radiation. Crystals were mounted on a MiTeGen MicroMount and collected at 100(2) K using an Oxford Cryosystems Cobra low‐temperature device. Data were collected using omega and phi scans and were corrected for Lorentz and polarization effects using the APEX software suite.^[33]^ Data were corrected for absorption effects using the multi‐scan method (SADABS).^[34]^ Using Olex2, the structure was solved with the XT structure solution program, using the intrinsic phasing solution method and refined against |F^2^| with XL using least‐squares minimization.^[35]^ The C and N bound H atoms were placed in their expected calculated positions and refined as riding model: N−H=0.88 Å, C−H=0.95–0.98 Å, with *U*_iso_ (H)=1.5*U*_eq_ (C) for methyl H atoms and 1.2*U*_eq_ (C, N) for all other atoms other H atoms. Details of data refinements can be found in Table S1. All images were prepared by using Olex2.^[35a]^

**Normal‐structural decomposition**: The NSD method, as developed by Shelnutt and coworkers,^[7]^ was used to delineate, quantify, and illustrate the various distortions modes present in the tetrapyrrole macrocycles. Analysis was performed with the NSD online interface, available at https://www.sengegroup.eu/nsd.^[36]^

**General procedure of porphyrin condensation for the synthesis of 3, 5, 7, 9, and 10**: To a pre‐dried 2 L round‐bottomed flask, anhydrous dichloromethane (500 mL–1 L) was added and purged with argon. Benzaldehyde **3A**, **5A**, **7A**, **9A** or **10A** (0.9–1.1 eq.) and 3,4‐diethyl‐1H‐pyrrole **11** (1.00 eq.) were added and the reaction mixture was stirred at room temperature under a slow steady flow of argon. After 15 min, BF_3_×Et_2_O (0.1 eq.) was added and the reaction flask was shielded from ambient light. After stirring for 16 h DDQ was added and 3 h later triethylamine (0.1 eq.) was added to neutralize BF_3_×Et_2_O. The solution was concentrated *in vacuo* to yield the crude product mixture, washed with NaOH (1 M), water, and dried over anhydrous magnesium sulfate. The organic extract was concentrated *in vacuo* for further purification by silica gel flash column chromatography.

**5,10,15,20‐Tetrakis(2,6‐dimethoxyphenyl)‐2,3,7,8,12,13,17,18‐octaethylporphyrin [3]**: Synthesized *via* the General Procedure from 3,4‐diethyl‐1H‐pyrrole **11** (1 mL, 8.12 mmol, 1 eq.), 2,6‐dimethoxybenzaldehyde **3 A** (1.21 g, 7.31 mmol, 0.9 eq.), BF_3_×OEt_2_ (0.1 mL, 0.812 mmol, 0.1 eq.) and DDQ (1.84 g, 8.12 mmol, 1 eq.). The reaction mixture was filtered through a silica gel using CH_2_Cl_2_ to remove the unreacted aldehyde. The compound was then removed from the silica using CH_2_Cl_2_/MeOH (9 : 1) and gave compound **3** as a green powder [1.352 g, 1.28 mmol, 70 %] upon evaporation of the solvent under reduced pressure. M.p. <149–175 °C; *R*_f_=0.60 (SiO_2_, CH_2_Cl_2_:MeOH=9 : 1, v/v); ^1^H NMR (600 MHz, CDCl_3_, 25 °C): *δ*=0.28 (broad s, 24H, CH_2_ *CH_3_*), 2.23 (broad s, 8H, *CH_2_*CH_3_), 2.46 (broad s, 8H, *CH_2_*CH_3_), 3.89 (s, 24H, *o*‐OCH_3_), 6.89–6.91 (d, *J*=8.3 Hz 8H, H_aryl_), 7.66–7.68 ppm (t, *J*=8.2 Hz, 4H, H_aryl_); ^13^C NMR (100 MHz, CDCl_3_, 25 °C): *δ*=160.9, 143.5, 136.9, 132.6, 116.3, 107.2, 104.3, 56.0, 18.4, 14.3 ppm; UV‐vis (CHCl_3_+1 % NEt_3_): *λ_max_* (log *ϵ*)=463 (4.81), 591 (3.64), 635 (3.72), 692 nm (3.57); IR (ATR): ṽ=3206, 2935, 2833, 2219, 1579, 1472, 1451, 1252, 1107, 1018, 888, 775, 743, 715 cm^−1^; HRMS (MALDI) *m/z* calcd. for C_68_H_78_N_4_O_8_ [M+H]^+^: 1079.5892, found 1079.5898.

**2,3,7,8,12,13,17,18‐Octaethyl‐5,10,15,20‐tetrakis(2,4,6‐trimethoxyphenyl)porphyrin [5]**: Synthesized *via* the General Procedure from 3,4‐diethyl‐1H‐pyrrole **11** (1 mL, 8.12 mmol, 1 eq.), 2,4,6‐trimethoxybenzaldehyde **5A** (1.43 g, 7.31 mmol, 0.9 eq.), BF_3_×OEt_2_ (0.1 mL, 0.812 mmol, 0.1 eq.) and DDQ (1.84 g, 8.12 mmol, 1 eq.). The reaction mixture was filtered through a silica gel using DCM to remove the unreacted aldehyde. The compound was then removed from the silica using DCM/MeOH (9 : 1) and lead, after evaporation of the solvent under reduce pressure to a green powder, compound **5** [0.217 mg, 0. 181 mmol, 10 %]. M.p.=186–199 °C; R_f_=0.58 (SiO_2_, CH_2_Cl_2_:MeOH=9 : 1, v/v); ^1^H NMR (600 MHz, CDCl_3_, 25 °C): δ=0.44 (broad s, 24H, CH_2_ *CH_3_*), 2.32 (broad s, 8H, *CH_2_*CH_3_), 2.48 (broad s, 8H, *CH_2_*CH_3_), 3.79 (s, 24H, *o*‐OCH_3_), 4.06 (s, 12H, *o*‐OCH_3_), 6.47 ppm (d, 8H, H_aryl_), ^13^C NMR (100 MHz, CDCl_3_, 25 °C): δ=163.69, 161.64, 144.77, 11.09, 106.31, 90.96, 56.08, 55.79, 18.49, 15.30, 14.34 ppm; UV‐Vis (CHCl_3_): λ_max_ (log ϵ)=472 (4.84), 594 (3.62), 643 (3.73), 703 nm (3.64); IR (ATR): ṽ=2933, 2830, 2202, 1599, 1578, 1453, 1412, 1331, 1224, 1204, 1154, 1123, 1123, 1056, 1033, 951, 812 cm^−1^; HRMS (MALDI) *m/z* calcd. for C_40_H_38_N_4_O_6_ [M+H]^+^: 1199.6368, found 1199.6321.

**5,10,15,20‐Tetrakis(2,6‐dimethoxy‐4‐hydroxyphenyl) 2,3,7,8,12,13,17,18‐octaethylporphyrin [6]**: In a pre‐dried 50 mL round bottomed flask, 2,3,7,8,12,13,17,18‐octaethyl‐5,10,15,20‐tetrakis(2,4,6‐trimethoxyphenyl)porphyrin (**5**) (17.2 mg, 14.34 μmol, 1.0 eq.) was dissolved in dry CHCl_3_ (4 mL). Under an argon atmosphere the mixture was treated dropwise with BBr_3_ (1 M in CH_2_Cl_2_, 0.1 mL, 100 μmol, 7 eq). After 2 h of stirring the reaction at 25 °C, the solvent was removed under vacuum. The reaction mixture was dissolved in DCM/MeOH (9 : 1) and filtered through a silica gel using DCM/MeOH (9 : 1) to remove the impurities. The compound was then collected using MeOH, after evaporation of solvent under reduced pressure provided compound **6** as dark green powder [14.1 mg, 12.33 μmol, 86 %]; M.p.: >300 °C; *R*_f_=0.2 (SiO_2_, MeOH); ^1^H NMR (400 MHz, *d*‐methanol, 25 °C) δ=6.36 (s, 8H, *m*‐phenyl‐*H*), 3.76 (s, *J*=13.5 Hz, 24H, *‐OCH_3_*), 2.84–2.52 (m, 16H, −*CH_2_*), 0.65 (t, *J*=7.4 Hz, 24H, −CH_2_ *CH_3_*).^13^C NMR (100 MHz, *d*‐methanol, 25 °C): *δ*=161.79, 160.48, 146.06, 138.17, 108.63, 107.36, 94.87, 55.19, 18.28, 14.71 ppm; UV/Vis (methanol): *λ*_max_ [nm] (log *ϵ*[L mol^−1^ cm^−1^])=488 (4.97), 695 (4.11); IR (ATR) *ṽ*=3369.4 (br), 3194.8 (br), 2970, 2935.6, 2876, 2844, 2224.6, 2158, 1595, 1455.1, 1418, 1327.4, 1154, 1118.1, 1056, 1024, 960, 920, 814.48 cm^−1^; HRMS (MALDI) *m/z* calcd. for C_68_H_79_N_4_O_12_ [M+H]^+^: 1143.5689, found 1143.5625.

**5,10,15,20‐Tetrakis(3,5‐dimethoxyphenyl)‐2,3,7,8,12,13,17,18‐octaethylporphyrin [7]**: Synthesized *via* the General Procedure from 3,5‐dimethoxybenzaldehyde **7A** (1.95 g, 11.7 mmol, 1.11 eq.), 3,4‐diethyl‐1H‐pyrrole **11** (1.3 g, 10.55 mmol, 1.00 eq.), BF_3_×Et_2_O (0.01 mL, 0.8 mmol) and DDQ (2.76 g, 12.18 mmol). After aqueous work up, the organic extract was transferred directly to the column (Al_2_O_3_). Less polar side products and starting material were eluted first (CH_2_Cl_2_), followed by the collection of the major green band (CH_2_Cl_2_:MeOH=50 : 1 to 150 : 1 *v/v*). Removal of solvent *in vacuo* yielded the product **3** as a dark purple colored solid [0.545 g, 0.505 mmol, 19 %]; M.p.: 260 °C (decomposition); *R*_f_=0.25 (SiO_2_, Ethyl acetate); ^1^H NMR (400 MHz, CDCl_3_, 25 °C) δ=7.71 (s, 8H, *o*‐phenyl‐*H*), 6.95 (s, 4H, *p*‐phenyl‐*H*), 4.07 (s, 24H, *‐OCH_3_*), 2.62–2.08 (m, 16H, −*CH_2_*), 0.30 ppm (t, *J*=7.3 Hz, 24H, −*CH_3_*); ^13^C NMR (100 MHz, CDCl_3_, 25 °C): *δ*=160.26, 143.90, 138.39, 118.74, 115.32, 102.56, 56.25, 18.55, 15.83 ppm; UV/Vis (chloroform): *λ*_max_ [nm] (log *ϵ*[L mol^−1^ cm^−1^])=454.9 (5.28), 552.3 (4.11), 604.4 (3.78), 638.6 (3.62), 697.7 (3.48); HRMS (MALDI) *m/z* calcd. for C_68_H_79_N_4_O_8_ [M+H]^+^: 1079.5909, found 1079.5898. IR (ATR) *ṽ*=2971.7, 2933.58, 2875.7, 2838.29, 1665.29, 1586.79 (s), 1452.86, 1421.62, 1355.18, 1314.41, 1290.24, 1199.57, 1153.91, 1132.14, 1059.16, 1021.31, 948.20, 835.80, 800.92, 717.10, 687.61 cm^−1^_._

**5,10,15,20‐Tetrakis(3,5‐dihydroxyphenyl)‐2,3,7,8,12,13,17,18‐octaethylporphyrin [8]**: In a pre‐dried 100 mL round bottomed flask, 2,3,7,8,12,13,17,18‐octaethyl‐5,10,15,20‐tetrakis(3,5‐dimethoxyphenyl)porphyrin **7** (550 mg, 0.506 mmol, 1.0 eq.) was dissolved in dry CH_2_Cl_2_ (60 mL). Under an argon atmosphere at 0 °C the mixture was treated dropwise with BBr_3_ (1 M in CH_2_Cl_2_, 10.5 mL, 10.5 mmol, 16 eq.). The reaction flask was warmed to room temperature and after 18 h of stirring, the mixture was added dropwise to ice water (50 mL). NaOH (1 M, 100 mL) was slowly added and washed with dichloromethane (2×50 mL). The aqueous phase was acidified with conc. HCl until the product started to coagulate. The green solid was filtered and washed with water and dichloromethane. The green solid was dissolved with methanol and after evaporation of solvent under reduced pressure provided a dark green crystalline solid **8**. [519 mg, 0.499 mol, 76 %]; M.p.: >300 °C; *R*_f_=0.63 (SiO_2_, MeOH); ^1^H NMR (400 MHz, d‐methanol, 25 °C) δ=7.40 (s, 8H, *o*‐phenyl‐*H*), 6.83 (s, 4H, *p*‐phenyl‐*H*), 2.67–2.42 (m, 16H, −*CH_2_*), 0.52 ppm (t, *J*=7.4 Hz, 24H, −CH_2_ *CH_3_*); ^13^C NMR (100 MHz, d‐methanol, 25 °C): *δ*=158.41, 143.60, 139.10, 138.95, 118.60, 115.86, 104.08, 18.19, 14.96 ppm; UV/Vis (Methanol): *λ*_max_ [nm] (log *ϵ*[L mol^−1^ cm^−1^])=475.9 (5.36), 689.2 (4.45); HRMS (MALDI) *m/z* calcd. for C_60_H_63_N_4_O_8_ [M]^+^: 967.4617, found 967.4646. IR (ATR) *ṽ*=3148.18 (br), 2972.31, 2933.66, 2873, 1587.88 (s), 1502.13, 1439.13, 1354.45, 1292.03, 1199.71, 1152.15 (s), 1054.38, 1003.66, 994.74, 980.09, 951.42, 886.88, 847.24, 809.12, 777.76, 685.77 cm^−1^.

**3,5‐Dipivaloyloxybenzaldehyde [9 A]**: In a 250 mL RBF at room temperature, 3,5‐dihydroxybenzaldehyde (0.5 g, 3.62 mmol, 1.0 eq.) was dissolved in acetonitrile (100 mL). *N,N*‐diisopropylethylamine (5 mL, 28.96 mmol, 8.0 eq.) was added. The reaction was treated dropwise with pivaloyl chloride (1.75 mL, mol, eq.) and stirred for 1 h. The crude mixture was extracted with dichloromethane, washed with NaHCO_3_ (1×150 mL), brine (1×150 mL) and water (1×150 mL). The organic phase was dried with anhydrous magnesium sulfate, filtered and evaporated *in vacuo* to yield a white crystalline solid **9A**. [1.069 g, 3.49 mmol, 96 %]; M.p.: 146.5 °C; *R*_f_=0.89 (SiO_2_, EtOAc/*n‐*hexane 1 : 1 v/v); ^1^H NMR (400 MHz, CDCl_3_, 25 °C) δ=9.99 (s, 1H, CO*H*), 7.51 (d, *J*_m_ *=*2.17 Hz, 2H, *o‐*phenyl‐*H*), 7.17 (t, *J*_m=_2.1 Hz, 1H, *p‐*phenyl‐*H*), 1.39 ppm (s, 18H, −*CH_3_*); ^13^C NMR (100 MHz, CDCl_3_, 25 °C): *δ*=190.29, 176.47, 152.09, 138.00, 121.50, 119.79, 39.22, 27.06, 26.53 ppm; HRMS (APCI) *m/z* calcd. for C_17_H_23_O_5_ [M]^+^: 307.1535, found 307.1540; IR (ATR) *ṽ*=2974.91, 2937.33, 2874.84, 1752.35 (CO stretch), 1697.34, 1592.1, 1480.72, 1396.93, 1368.58, 1300.07, 1269.26, 1091.23 (s, br), 1030.07 (sh), 980.16, 941.15, 905.88, 803.73, 803.41, 757.89, 732.27, 672.11, 597.75, 552.37 cm^−1^.

**5,10,15,20‐Tetrakis(3,5‐dipivaloyloxyphenyl)‐2,3,7,8,12,13,17,18‐octaethylporphyrin [9]**: A: In a pre‐dried 50 mL Schleck flask precursor porphyrin **8** (46 mg, 0.048 mmol, 1 eq.) was stirred in THF (8 mL) with pivaloyl chloride (0.53 mL, 3 mmol, 64 eq.) in the presence of *N,N‐*diisopropylamine (0.374 mL, 3 mmol, 64 eq.) for 5 h. Aqueous workup with saturated NaHCO_3_ (2×50 mL) and water (50 mL) followed by evaporation of solvent *in vacuo* yielded product **9** as a green crystalline solid [52 mg, 0.032 mmol, 67 %]. Introduction of eight nonpolar *tert*‐butyl groups was easily monitored by TLC due to the large difference in R_f_ values between the highly polar starting material and product.

B: Synthesized *via* the General Procedure using 3,5‐dipivaloyloxybenzaldehyde **9A** (1.069 g, 3.49 mmol, 1.0 eq.), 3,4‐diethylpyrrole **11** (481 mg, 3.90 mmol, 1.1 eq.), BF_3_×Et_2_O (0.05 mL, 4.46 mmol) and DDQ (1.2 g, 5.29 mmol) in CH_2_Cl_2_ (500 mL). After aqueous work up, the residue was transferred directly to a silica gel column and non‐polar impurities eluted with CH_2_Cl_2_. The crude mixture was acidified with 1 % TFA and product was collected *via* silica gel column (CH_2_Cl_2_/EtOAc 4 : 1 v/v). Aqueous work up with NaOH (1 M, 2×150 mL) and water (150 mL) followed by evaporation of solvent *in vacuo* yielded the neutralized product as green crystalline solid. This was recrystallized by slow diffusion in DMSO/acetonitrile to yield dark green crystals of **9** [0.809 g, 0.493 mmol, 57 %]; M.p.: 276 °C (decomposition); *R*_f_=0.72 (SiO_2_, *n‐*hexane/EtOAc 1 : 1 v/v); ^1^H NMR (600 MHz, CDCl_3_, 25 °C) δ=8.17 (s, 8H, *o*‐phenyl‐*H*), 7.38 (s, 4H, *p‐*phenyl‐*H*), 2.56–2.30 (m, 16H, −*CH_2_*), 1.51 (s, 72H, *t‐*Bu), 0.42 ppm (t, *J=*7.4 Hz, 24H, −CH_2_C*H_3_*); ^13^C NMR (100 MHz, CDCl_3_, 25 °C): *δ*=176.77, 151.21, 144.00, 139.30, 138.71, 127.20, 116.79, 116.52, 39.33, 27.24, 18.71, 15.73 ppm; UV/Vis (chloroform): *λ*_max_ [nm] (log *ϵ*[L mol^−1^ cm^−1^])=456.3 (5.17), 553.4 (4.10), 600.4 (3.48), 631.0 (3.62), 702.1 (3.62); HRMS (MALDI) *m/z* calcd. for C_100_H_127_N_4_O_16_ [M+H]^+^: 1639.9247, found 1639.9216; IR (ATR) *ṽ*=2970.96, 2934.29, 2873.32, 1754.12 (s, CO stretch) 1605.2, 1587.04, 1479.79, 1458.96, 1428.24, 1397.00, 1367.91 1268.48 1163.57, 1090.14 (s, br), 1028.82 (sh), 978.72, 945.95, 904.07, 862.88, 822.38, 798.92, 688.04, 617.29, 559.51 cm^−1^_._

**5‐Pivaloyloxy‐2‐nitrobenzaldehyde [10 A]**: In a 250 mL RBF at room temperature, 5‐hydroxy‐2‐nitrobenzaldehyde (0.500 g, 2.99 mmol, 1.0 eq.) was dissolved in acetonitrile (120 mL). *N,N*‐Diisopropylethylamine (5 mL, 28.96 mmol, 8.0 eq.) was added. The reaction was treated dropwise with pivaloyl chloride (1.46 mL, 11.98 mmol, 4 eq.) and stirred for 1 h. The crude mixture was extracted with dichloromethane, washed with NaHCO_3_ (1×150 mL), brine (1×150 mL) and water (1×150 mL). The organic phase was dried with anhydrous magnesium sulfate, filtered, and concentrated *in vacuo* to yield a crystalline solid. Purification by silica gel column (CH_2_Cl_2_) collected a pale yellow fraction, which was dried to afford the product **10A** as a white crystalline solid [0.359 g, 1.43 mmol, 75 %]; M.p.: 92–96 °C; *R*_f_=0.64 (SiO_2_, CH_2_Cl_2_); ^1^H NMR (400 MHz, CDCl_3_, 25 °C) δ=10.47 (s, 1H, CO*H*), 8.22 (d, *J*_o_ *=*8.9 Hz, 1H, *m‐*phenyl‐*H*), 7.65 (d, *J*_m_ *=*2.4 Hz, 1H, *o‐*phenyl‐*H*), 7.49 (dd, *J*_o_ *=*9.0 Hz, *J*_m_=2.8 Hz, 1H, *p‐*phenyl‐*H*), 1.40 ppm (s, 9H, *t‐*Bu); ^13^C NMR (100 MHz, CDCl_3_, 25 °C): *δ*=187.30, 173.91, 155.38, 146.25, 133.23, 126.50, 122.63, 39.40, 26.98 ppm; HRMS (APCI) *m/z* calcd. for C_12_H_12_NO_5_ [M]^−^: 250.0721, found 250.0718; IR (ATR) *ṽ*=2973.75, 2873.12, 1757.43 (s, C=O stretch.), 1610.98, 1578.45, 1525.08 (s, NO asym. stretch.), 1465.06, 1397.46, 1340.72 (s, NO sym. stretch), 1260.34, 1182.77, 1093.08 (s, C−O stretch.), 1023.02 (sh, C−O stretch.), 951.08, 900.54, 850.25, 751.93, 696.63, 652.16, 622.27, 574.91, 539.58 cm^−1^.

**2,3,7,8,12,13,17,18‐Octaethylporphyrin‐5,10,15,20‐tetrakis(5‐pivaloyloxy‐2‐nitrophenyl) [10]**: Synthesized *via* the General Procedure from 5‐pivaloyloxy‐2‐nitrobenzaldehyde **10A** (0.934 g; 3.72 mmol; 1.0 eq.), 3,4‐diethylpyrrole **9** (0.504 g; 4.09 mmol; 1.1 eq.), BF_3_×Et_2_O (0.075 mL, 0.66 mmol) and DDQ (1.2 g, 5.29 mmol). The crude reaction mixture was quenched with 10 % triethylamine. After aqueous work up, the concentrated organic extract was purified by silica gel column (CH_2_Cl_2_/EtOAc 10 : 1 *v/v*) and eluted (CH_2_Cl_2_/TEA 10 : 1 *v/v*) as a dark brown/green fraction. The residue was transferred to a 250 mL RBF and dissolved in chloroform (100 mL). *N,N*‐Diisopropylethylamine (1.39 mL, 7.97 mmol, 8.0 eq.) was added and the reaction was treated dropwise with pivaloyl chloride (0.5 mL, 3.98 mmol, 4 eq.) and stirred for 1 h. The crude mixture was quenched with a few drops of NaOH, followed by extraction with dichloromethane, and washed with NaHCO_3_ (1×150 mL) and water (1×150 mL). The organic phase was dried with anhydrous magnesium sulfate, filtered, and dried under reduced pressure. Purification by a silica gel column removed black impurities (CH_2_Cl_2_/acetone 10 : 1 *v/v*) and the desired intermediate was collected (CH_2_Cl_2_/TEA 10 : 1 *v/v*) as a brown colored product **10** [0.558 g, 3.93 mmol, 20 %] HRMS (MALDI) *m/z* calcd. for C_80_H_91_N_8_O_16_ [M+H]^+^: 1419.6553, found 1419.6549.

**[5,10,15,20‐Tetrakis(5‐pivaloyloxy‐2‐nitrophenyl)‐2,3,7,8,12,13,17,18‐octaethylporphyrinato]nickel(II) [12]**: In a 250 mL RBF, the atropisomeric mixture of **10** (0.335 g, 0.236 mmol,1.0 eq.) was dissolved in toluene (100 mL) at room temperature. Nickel(II) acetylacetonate (0.303 g, 1.180 mmol, 5.0 eq.) was added and the reaction was heated to reflux at 111 °C. After 2 h, the mixture was cooled, and solvent was evaporated *in vacuo*. The mixture was dissolved in a small volume of dichloromethane and transferred to a silica gel column (CH_2_Cl_2_). Three green fractions (CH_2_Cl_2_) were collected and dried under reduced pressure to yield three products as dichromatic (green/purple) crystalline solids:

*α,β,α*,*β*‐**12**: [87.2 mg, 0.059 mmol, 25 %]; M.p.: >300 °C; *R*_f_=0.82 (SiO_2_, CH_2_Cl_2_); ^1^H NMR (600 MHz, CDCl_3_, 25 °C): *δ*=8.35 (d, *J*_o_ *=*8.7 Hz, 4H, *m*‐phenyl‐*H*), 7.70 (d, *J*_m_ *=*2.6 Hz, 4H, *o‐*phenyl‐*H*), 7.57 (dd, *J*_o_ *=*8.8 Hz, *J*_m_ *=*2.2 Hz, 4H, *p*‐phenyl‐*H*), 2.40 (br, 16H, −C*H_2_*), 1.45 (s, 36H, *t*‐Bu), 0.62 (br, 24H, −CH_2_C*H_3_*) ppm; ^13^C NMR (100 MHz, CDCl_3_, 25 °C): *δ*=176.45, 153.18, 148.90, 144.03, 136.41, 130.28, 125.60, 123.10, 111.40, 39.40, 27.09, 19.55 ppm; UV/Vis (chloroform): *λ*_max_ [nm] (log *ϵ*[L mol^−1^ cm^−1^])=442.6 (5.00), 572.2 (4.07), 611.3 (4.04); HRMS (MALDI) *m/z* calcd. for C_80_H_88_N_8_NiO_16_ [M]^+^: 1474.5672, found 1474.5699; IR (ATR) *ṽ*=2972.50, 2934.84, 2873.17, 1757.33 (s, C=O stretch.), 1610.59, 1578.45, 1523.98 (s, ‐NO asym. stretch.), 1466.13 (m, C*H_2_* bend), 1397.58, 1342.75 (s, ‐NO sym. stretch.), 1261.73, 1184.14, 1081.52 (s, C−O stretch.), 1023.05 (sh, C−O stretch.), 950.37, 900.54, 853.95, 828.03, 801.43, 752.00, 699.37, 651.67, 617.94 cm^−1^_._

*α_2_β_2_*‐**12**: [50.7 mg, 0.034 mmol, 15 %]; M.p.: 279–280 °C (decomposition); *R*_f_=0.56 (SiO_2_, CH_2_Cl_2_); ^1^H NMR (600 MHz, CDCl_3_, 25 °C): *δ*=8.35 (d, *J*_o_ *=*8.7 Hz, 4H, *m*‐phenyl‐*H*), 7.90 (br, 4H, *o‐*phenyl‐*H*), 7.57 (dd, *J*_o_ *=*8.9 Hz, *J*_m_ *=*2.0 Hz, 4H, *p‐*phenyl‐*H*), 2.40 (br, 16H, −C*H_2_*), 1.46 (s, 36H, *t*‐Bu), 0.63 (br, 24H, −CH_2_C*H_3_*) ppm; ^13^C NMR (100 MHz, CDCl_3_, 25 °C): *δ*=176.37, 152.89, 149.31, 144.80, 144.18, 136.32, 129.92, 125.79, 122.95, 39.40, 27.11, 19.52; UV/Vis (chloroform): *λ*_max_ [nm] (log *ϵ*[L mol^−1^ cm^−1^])=442.1 (4.97), 573.1 (4.05), 609.4 (4.01); HRMS (MALDI) *m/z* calcd. for C_80_H_88_N_8_NiO_16_ [M]^+^: 1474.5672, found 1474.5707; IR (ATR) *ṽ*=2973.48, 2934.36, 2873.37, 1758.47 (s, C=O stretch.), 1611.04, 1578.83, 1525.46 (s, ‐NO asym. stretch), 1465.10 (m, C*H_2_*‐ bend.), 1397.75, 1341.15 (s, ‐NO sym. stretch.), 1260.08, 1182.72, 1092.89 (s, C−O stretch.), 1022.92 (sh, C−O stretch.), 951.10, 900.74, 851.23, 826.04, 800.37, 752.30, 697.90, 652.48, 620.27, 578.09 cm^−1^_._

*α_3_β*‐**12**: [76.6 mg, 0.052 mmol, 22 %]; M.p.: 272–276 °C (decomposition); *R*_f_=0.44 (SiO_2_, CH_2_Cl_2_); ^1^H NMR (600 MHz, CDCl_3_, 25 °C): *δ*=8.44–8.32 (m, 4H, *m*‐phenyl‐*H*), 8.10 (s, 1H, *o‐*phenyl‐*H*), 8.04–7.68 (br, 2H, *o‐*phenyl‐*H*), 7.61 (s, 1H, *o‐*phenyl‐*H*), 7.60–7.53 (m, 4H, *p‐*phenyl‐*H*), 2.41 (br, 16H, −C*H_2_*), 1.45 (s, 36H, *t*‐Bu), 0.63 (br, 24H, −CH_2_C*H_3_*) ppm; ^13^C NMR (100 MHz, CDCl_3_, 25 °C): *δ*=176.36, 153.32, 152.86, 152.62, 149.59, 148.48, 143.92, 136.48, 136.30, 136.12, 130.70, 129.75, 129.57, 126.08, 125.90, 125.52, 123.12, 122.94, 122.80, 111.76, 111.27, 39.39, 27.10, 19.53; UV/Vis (chloroform): *λ*_max_ [nm] (log *ϵ*[L mol^−1^ cm^−1^])=442.7 (5.01), 571.8 (4.10), 610.8 (4.06); HRMS (MALDI) *m/z* calcd. for C_80_H_88_N_8_NiO_16_ [M]^+^: 1474.5672, found 1474.5724; IR (ATR) *ṽ*=2973.74, 2934.01, 2873.13, 1757.65 (s, C=O stretch.), 1610.96, 1578.41, 1525.07 (s, ‐NO asym. stretch), 1465.21 (m, C*H_2_* bend.), 1397.54, 1340.27 (s, ‐NO sym. stretch.), 1260.09, 1182.28, 1092.81 (s, C−O stretch.), 1022.79 (sh, C−O stretch.), 951.19, 900.55, 850.52, 826.47, 800.62, 752.16, 697.14, 651.97, 620.27, 575.07 cm^−1^_._

**Supporting Information** (see footnote on the first page of this article): Spectroscopic data of all compounds and X‐ray crystallographic data.

Deposition Numbers 2058266 (for *α,β,α,β‐**12***), 2058269 (for *α_2_β_2_‐**12***), 2058267 (for *α_3_β*‐**12**), 2058270 (for **8**), 2058268 (for **9**), 2058264 (for **10A**), and 2058265 (for **9A**) contain the supplementary crystallographic data for this paper. These data are provided free of charge by the joint Cambridge Crystallographic Data Centre and Fachinformationszentrum Karlsruhe Access Structures service www.ccdc.cam.ac.uk/structures.

## Conflict of interest

The authors declare no conflict of interest.

## Supporting information

As a service to our authors and readers, this journal provides supporting information supplied by the authors. Such materials are peer reviewed and may be re‐organized for online delivery, but are not copy‐edited or typeset. Technical support issues arising from supporting information (other than missing files) should be addressed to the authors.

Supplementary
