# Supplementary material for: Strategic Synthesis of ‘Picket Fence’ Porphyrins Based on Nonplanar Macrocycles
Source: European J Org Chem. 2021 Mar 22;2021(12):1871–82. doi: 10.1002/ejoc.202100154 (PMC8048935; doi:10.1002/ejoc.202100154)
Supplement: Supplementary file 1 — Supplementary [file EJOC-2021-1871-s001.pdf]

European Journal of Organic Chemistry

Supporting Information

Strategic Synthesis of 'Picket Fence' Porphyrins Based on Nonplanar Macrocycles**

Karolis Norvaiša, Kathryn Yeow, Brendan Twamley, Marie Roucan, and Mathias O. Senge*

Table of Contents

Experimental Procedures 2
General Materials and Methods 2
Synthesis and Characterization of Compounds 3
*Synthesis and characterization of **3***..4
*Synthesis and characterization of **5***..7
*Synthesis and characterization of **6***..11
*Synthesis and characterization of **7***..14
*Synthesis and characterization of **8***..17
*Synthesis and characterization of **9A***...20
*Synthesis and characterization of **9***..22
*Synthesis and characterization of **10A***...25
*Synthesis and characterization of **10***..27
*Synthesis and characterization of **12***..28
Results and Discussion 37
Structural Determination of Isolated Structures..37
Demethylation and Substitution attempts...40
Stability of Individual Atropisomers 42
References 43

Experimental Procedures

General Materials and Methods

All chemicals were supplied by Sigma Aldrich, Acros Organics, Fluka, Frontier Scientific, and Fischer and handled without further purification unless otherwise stated. Anhydrous DCM used in large scale reactions, obtained *via* drying with phosphorus pentoxide followed by distillation, while smaller amounts of EtOH and toluene were used as commercially available HPLC grade solvents. Chloroform used in titration studies was neutralized with potassium carbonate and filtered over celite before carrying out any measurements. Condensation reactions were carried out under an argon atmosphere. Reactions involving moisture and/or air-sensitive reagents were carried out in pre-dried glassware and with standard Schlenk line techniques. The 3,4-diethyl-1H-pyrrole **11**[1] and [2,3,7,8,12,13,17,18-octaethyl-5,10,15,20-tetrakis(2-aminophenyl)porphyrin] **1**[2] was prepared *via* known procedures. Dichloromethane (CH_2Cl_2) was dried over P_2O_5. Yields refer to chromatographically and spectroscopically (^{1}H NMR) homogeneous material, unless otherwise noted. Reactions were monitored by thin layer chromatography (TLC) and absorption spectroscopy.

Analytical thin layer chromatography was performed using silica gel 60 (fluorescence indicator F254, precoated sheets, 0.2 mm thick, 20 cm × 20 cm; Merck) or aluminum oxide 60 (neutral, F254; Merck) plates and visualized by UV irradiation (λ = 254 nm).

Column chromatography was carried out using Fluka Silica Gel 60 (230–400 mesh; Merck) or aluminum oxide (neutral, activated with 6% H_2O, Brockman Grade III). Mobile phases are given as (*v/v*).

Mass spectrometry was performed with a Q-Tof Premier Waters MALDI quadrupole time-of-flight (Q-TOF) mass spectrometer equipped with Z-spray electrospray ionization (ESI) and matrix-assisted laser desorption ionization (MALDI) sources in positive mode with DCTB *trans*-2-[3-(4-*tert*-butylphenyl)-2-methyl-2-propenylidene]malononitrile (DCTB) as the matrix. ESI mass spectra were acquired in positive modes as required, using a Micromass time-of-flight mass spectrometer (TOF) interfaced to a Waters 2960 HPLC or a Bruker microTOF-Q III spectrometer interfaced to a Dionex UltiMate 3000 LC. Atmospheric pressure chemical ionization (APCI) experiments were performed on a Bruker microTOF-Q III spectrometer interfaced to a Dionex UltiMate 3000 LC.

Melting points are uncorrected and were measured with a Stuart SP-10 melting point apparatus.

NMR spectra were recorded on a Bruker Advance III 400 MHz, a Bruker Advance HD 400 and an Agilent 400 spectrometer for ^{1}H (400.13 MHz) and ^{13}C (100.61 MHz) NMR spectra. A Bruker Ultrashield 600 spectrometer was employed for ^{1}H (600.13 MHz) and ^{13}C (150.90 MHz) NMR spectra. All NMR experiments were performed at 25 ºC. Resonances δ are given in ppm units and referenced to the deuterium peak in the NMR solvents, d_4-methanol (δ_H = 4.87, 3.31 ppm, δ_C = 49.1 ppm). $CDCl_3$ (δ_H = 7.26 ppm, δ_C = 77.2 ppm). Signal multiplicities are abbreviated as follows: singlet = s, doublet = d, triplet = t, multiplet = m.

Single crystal X-ray crystallography: Diffraction data for all compounds were collected on a a Bruker D8 Quest ECO or Bruker APEX 2 DUO CCD diffractometer using graphite-monochromated Mo-K_α (λ = 0.71073 Å) or Incoatec IµS Cu-K_α (λ = 1.54178 Å) radiation. Crystals were mounted on a MiTeGen MicroMount and collected at 100(2) K using an Oxford Cryosystems Cobra low-temperature device. Data were collected using omega and phi scans and were corrected for Lorentz and polarization effects using the APEX software suite.[3] Data were corrected for absorption effects using the multi-scan method (SADABS).[4]

UV-Vis absorption measurements were recorded in solutions using a Specord 250 spectrophotometer from Quartz Glass 10mm 6030-UV (1 cm path length quartz cell).

IR measurements were done on a PerkinElmer Spectrum 100 FT-IR.

Shelnutt's NSD (normal structural decomposition) method was used to delineate, quantify and illustrate the various distortions modes present in the tetrapyrrole macrocycles.[5] NSD calculations were performed with the NSD online interface, available at https://www.sengegroup.eu/nsd.[6]

Synthesis and Characterization of Compounds

The general procedure of porphyrin condensation to synthesize 3, 5, 7, 9 and 10.

To a pre-dried 2L round-bottomed flask, anhydrous dichloromethane (500 mL – 1 L) was added and purged with argon. Benzaldehyde **3A, 5A, 7A, 9A or 10A** (0.9 – 1.1 eq.) and 3,4-diethyl-1H-pyrrole **11** (1.00 eq.) were added and the reaction mixture was stirred at room temperature under a slow steady flow of argon. After 15 min, $BF_3{\times}Et_2O$ (0.1 eq.) was added and the reaction flask was shielded from ambient light. After stirring for 16 h DDQ was added and 3 h later triethylamine (0.1 eq.) was added to neutralize $BF_3{\times}Et_2O$. The solution was concentrated *in vacuo* to yield the crude product mixture, washed with NaOH (1 M), water, and dried over anhydrous magnesium sulfate. The organic extract was concentrated *in vacuo* for further purification by silica gel flash column chromatography.

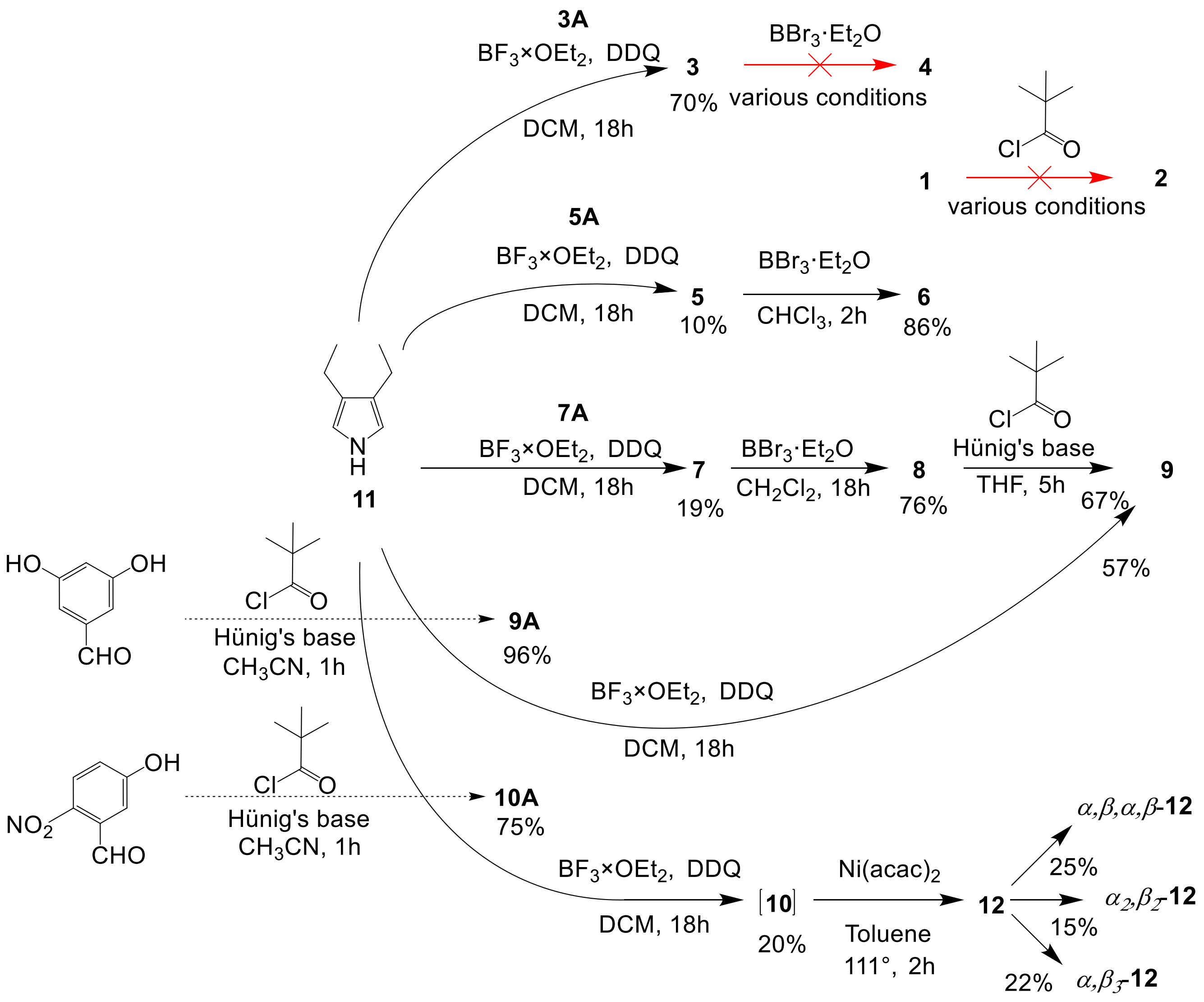

Scheme S1. Synthetic scheme for the preparation of the target compounds.

*Synthesis and characterization of **3***

2,3,7,8,12,13,17,18-Octaethyl-5,10,15,20-tetrakis(2,6-dimethoxyphenyl)porphyrin [3]

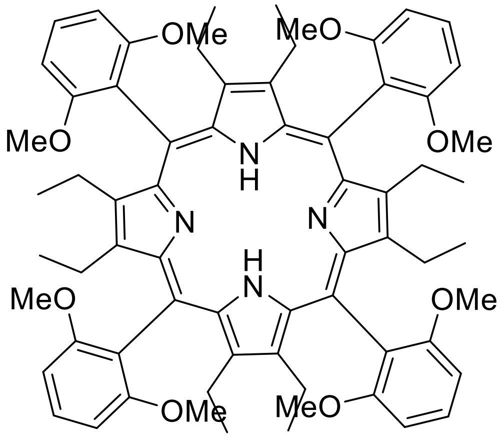

Synthesized *via* the General Procedure from 3,4-diethyl-1H-pyrrole **11** (1 mL, 8.12 mmol, 1 eq.), 2,6-dimethoxybenzaldehyde **3A** (1.21 g, 7.31 mmol, 0.9 eq.), $BF_3 \times OEt_2$ (0.1 mL, 0.812 mmol, 0.1 eq.) and DDQ (1.84 g, 8.12 mmol, 1 eq.). The reaction mixture was filtered through a silica gel using CH_2Cl_2 to remove the unreacted aldehyde. The compound was then removed from the silica using CH_2Cl_2/MeOH (9:1) and gave compound **3** as a green powder [1.352 g, 1.28 mmol, 70%] upon evaporation of the solvent under reduced pressure. M.p. <149–175 °C; R_f = 0.60 (SiO_2, CH_2Cl_2:MeOH = 9:1, v/v); ^1H NMR (600 MHz, $CDCl_3$, 25 °C): δ = 0.28 (broad s, 24H, CH_2CH_3), 2.23 (broad s, 8H, CH_2CH_3), 2.46 (broad s, 8H, CH_2CH_3), 3.89 (s, 24H, *o*-OCH_3), 6.89-6.91 (d, J = 8.3 Hz 8H, H_{aryl}), 7.66-7.68 ppm (t, J = 8.2 Hz, 4H, H_{aryl}); ^{13}C NMR (100 MHz, $CDCl_3$, 25 °C): δ = 160.9, 143.5, 136.9, 132.6, 116.3, 107.2, 104.3, 56.0, 18.4, 14.3 ppm; UV-vis ($CHCl_3$ + 1% NEt_3): λ_{max} (log ε) = 463 (4.81), 591 (3.64), 635 (3.72), 692 nm (3.57); IR (ATR): $\tilde{\nu}$ = 3206, 2935, 2833, 2219, 1579, 1472, 1451, 1252, 1107, 1018, 888, 775, 743, 715 cm^{-1}; HRMS (MALDI) *m/z* calcd. for $C_{68}H_{78}N_4O_8$ $[M+H]^+$: 1079.5892, found 1079.5898.

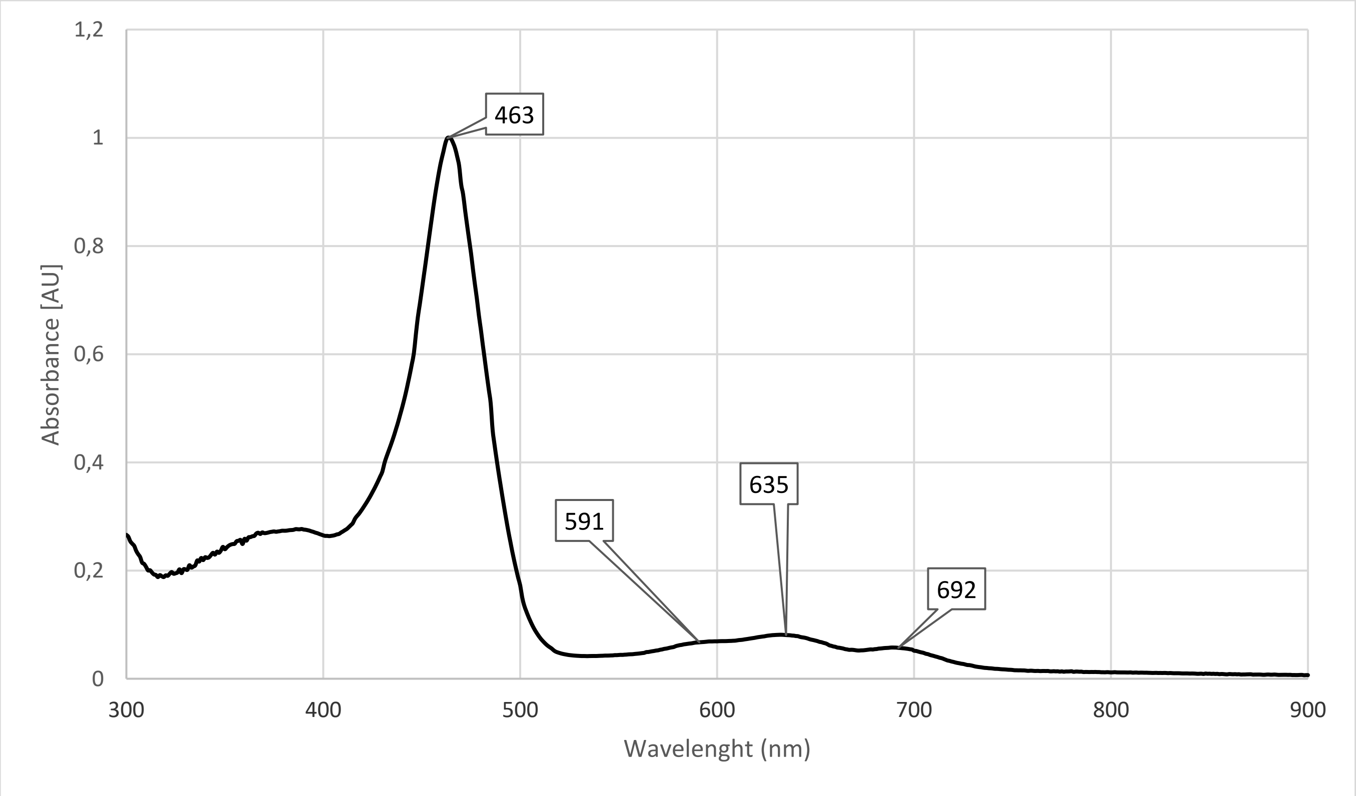

Figure S1. UV-vis spectrum of **3** in $CHCl_3$.

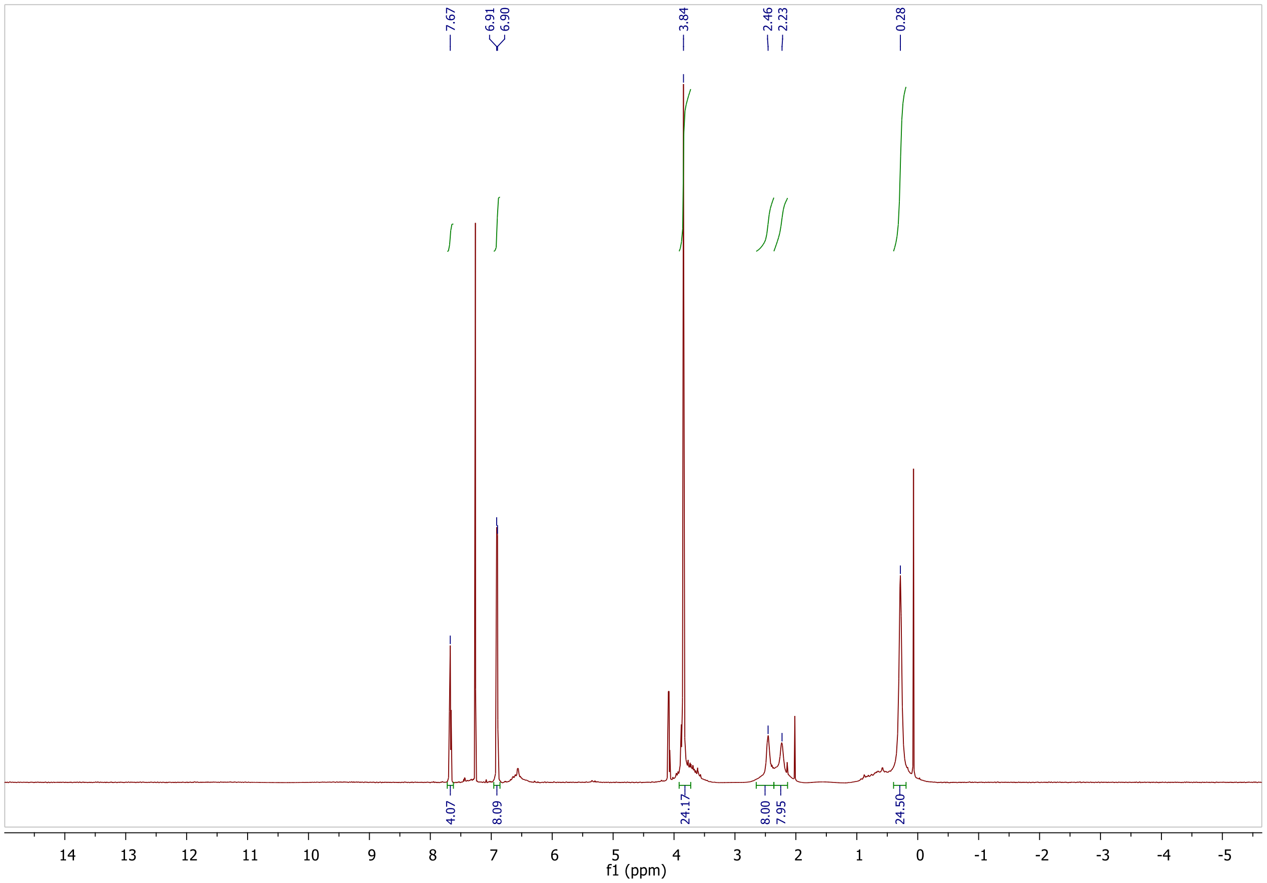

Figure S2. ^{1}H NMR spectrum of **3** (600 MHz, *d*-chloroform, 25 °C).

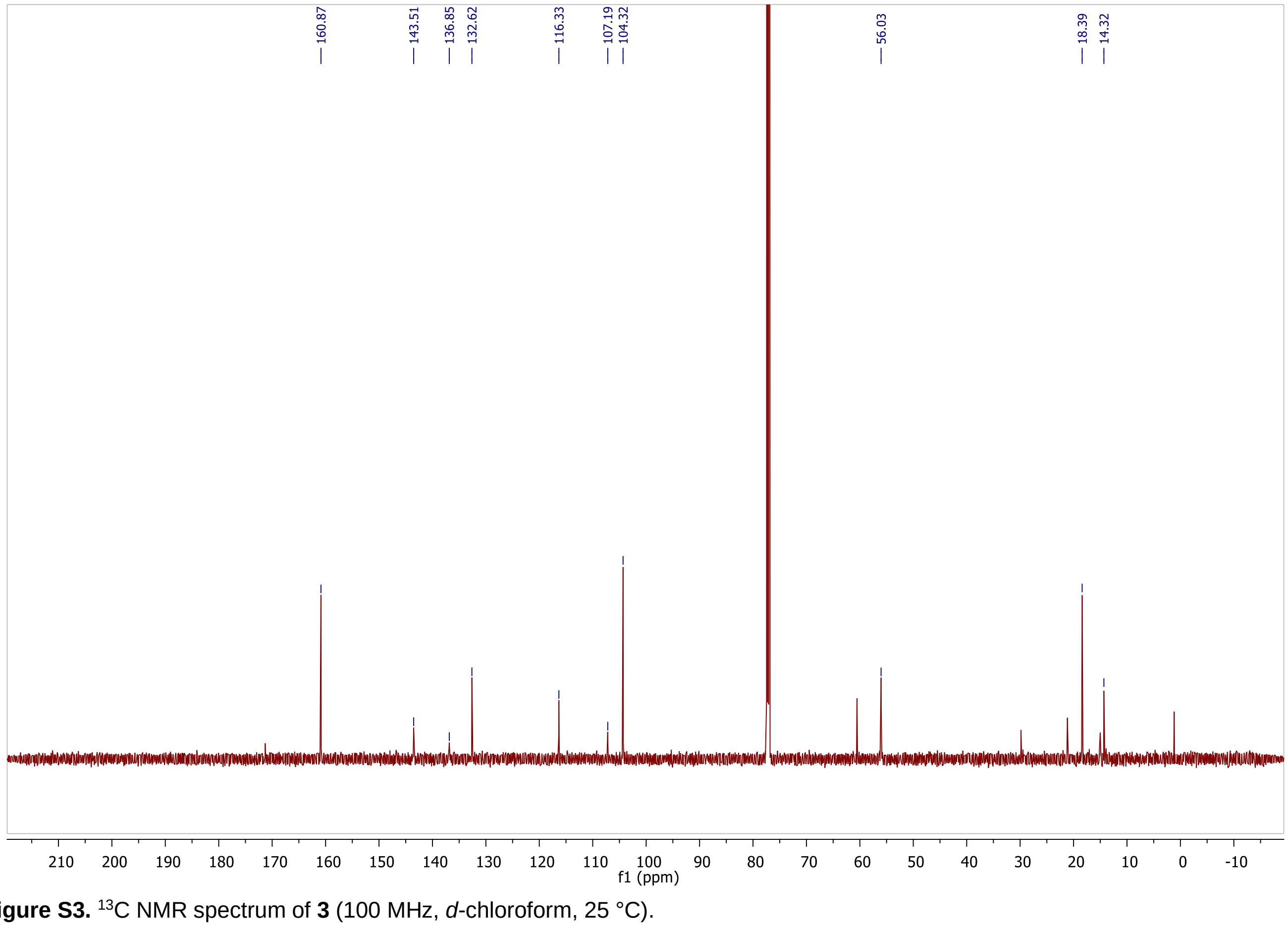

Figure S3. ^{13}C NMR spectrum of **3** (100 MHz, *d*-chloroform, 25 °C).

Minimum:				-1.5			
Maximum:		5.0	100.0	200.0			
Mass	Calc. Mass	mDa	PPM	DBE	i-FIT	i-FIT (Norm)	Formula
1079.5887	1079.5898	-1.1	-1.0	31.5	39.2	0.0	C68 H79 N4 O8

Figure S4. HRMS (MALDI) of **3**.

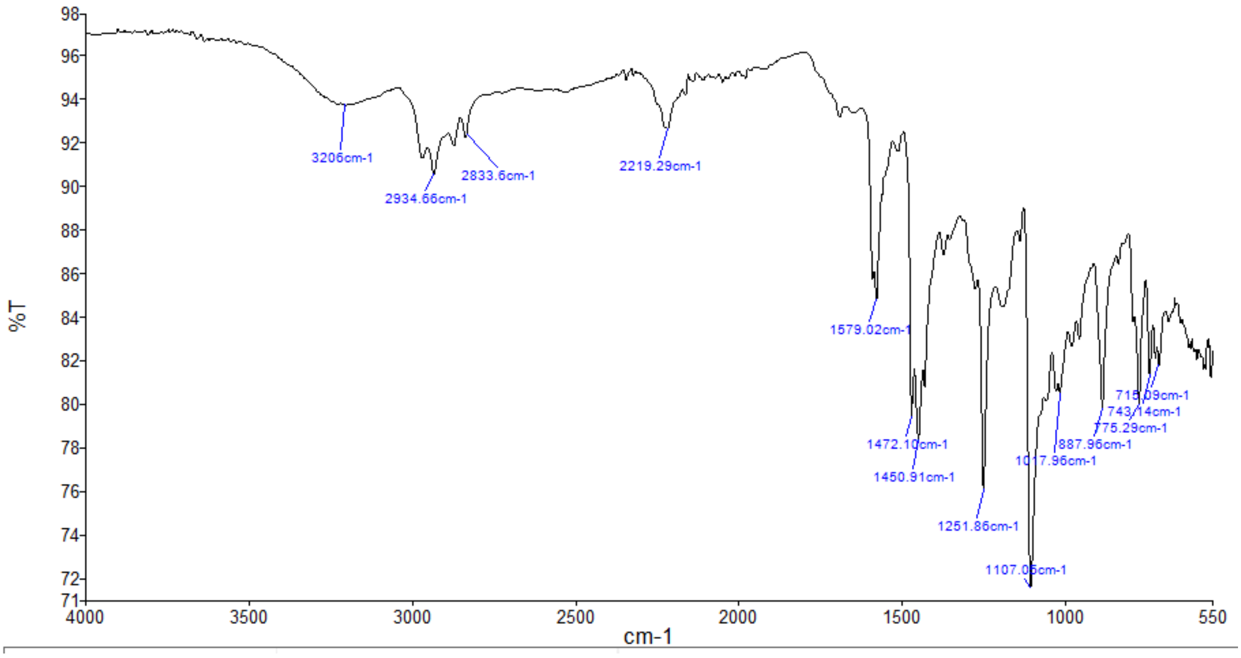

Figure S5. FTIR spectrum of **3**.

Synthesis and characterization of **5**

2,3,7,8,12,13,17,18-Octaethyl-5,10,15,20-tetrakis(2,4,6-trimethoxyphenyl)porphyrin [5]

Synthesized *via* the General Procedure from 3,4-diethyl-1H-pyrrole **11** (1 mL, 8.12 mmol, 1eq.), 2,4,6-trimethoxybenzaldehyde **5A** (1.43 g, 7.31 mmol, 0.9 eq.), BF_3×OEt_2 (0.1 mL, 0.812 mmol, 0.1 eq.) and DDQ (1.84 g, 8.12 mmol, 1 eq.). The reaction mixture was filtered through a silica gel using DCM to remove the unreacted aldehyde. The compound was then removed from the silica using DCM/MeOH (9:1) and lead, after evaporation of the solvent under reduce pressure to a green powder, compound **5** [0.217 mg, 0. 181 mmol, 10%]. M.p. = 186 – 199 °C; R_f = 0.58 (SiO_2, CH_2Cl_2:MeOH = 9:1, v/v); ^{1}H NMR (600 MHz, $CDCl_3$, 25 °C): δ = 0.44 (broad s, 24H, CH_2*CH_3*), 2.32 (broad s, 8H, *CH_2*CH_3), 2.48 (broad s, 8H, *CH_2*CH_3), 3.79 (s, 24H, *o*-OCH_3), 4.06 (s, 12H, *o*-OCH_3), 6.47 ppm (d, 8H, H_{aryl}), ^{13}C NMR (100 MHz, $CDCl_3$, 25 °C): δ = 163.69, 161.64, 144.77, 11.09, 106.31, 90.96, 56.08, 55.79, 18.49, 15.30, 14.34 ppm; UV-Vis ($CHCl_3$): λ_{max} (log ε) = 472 (4.84), 594 (3.62), 643 (3.73), 703 nm (3.64); IR (ATR): ṽ = 2933, 2830, 2202, 1599, 1578, 1453, 1412, 1331, 1224, 1204, 1154, 1123, 1123, 1056, 1033, 951, 812 cm^{-1}; HRMS (MALDI) m/z calcd. for $C_{40}H_{38}N_4O_6$ $[M+H]^+$: 1199.6368, found 1199.6321.

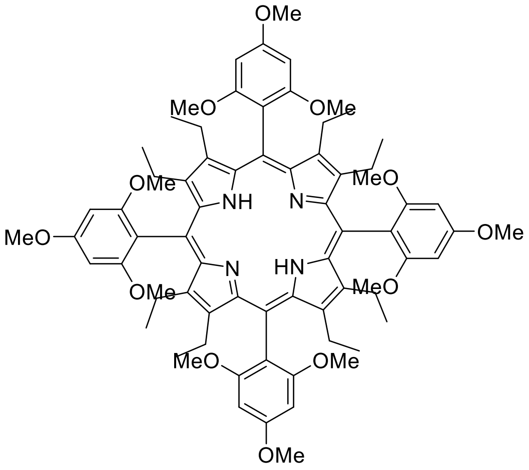

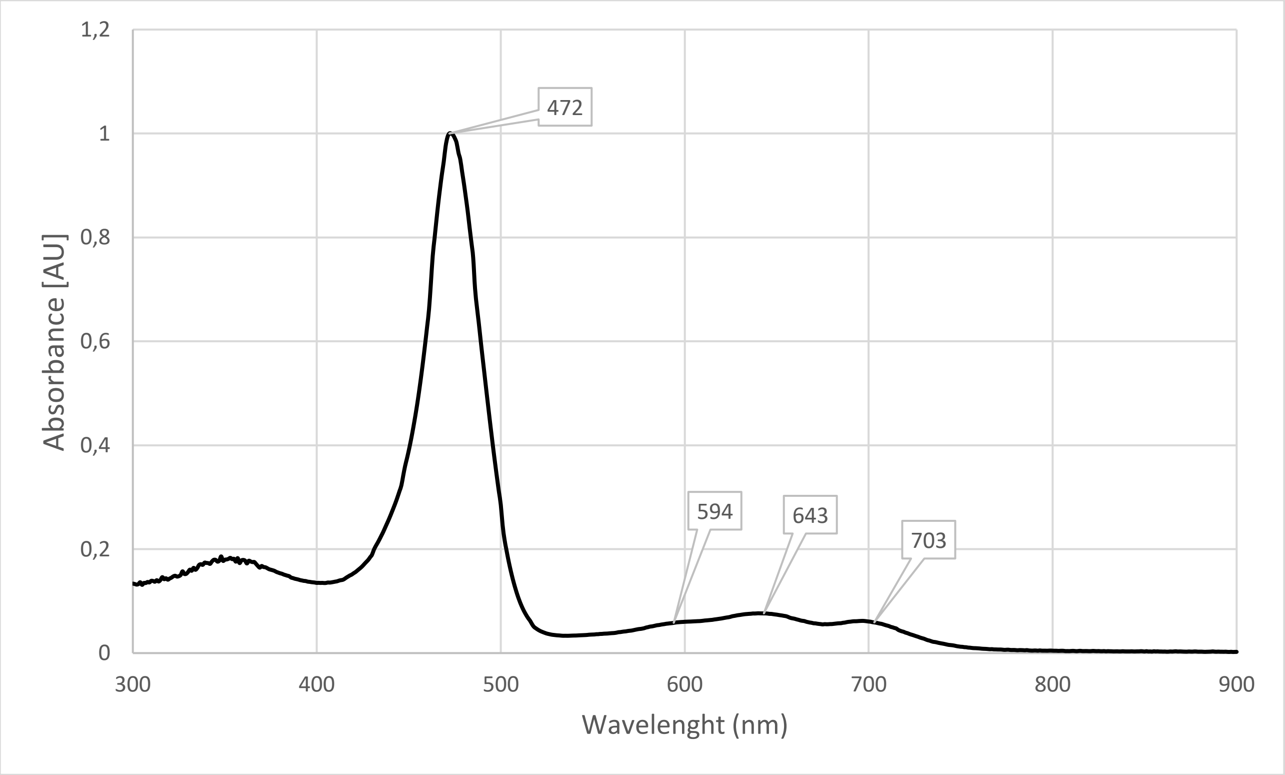

Figure S6. UV-vis spectrum of **5** in chloroform.

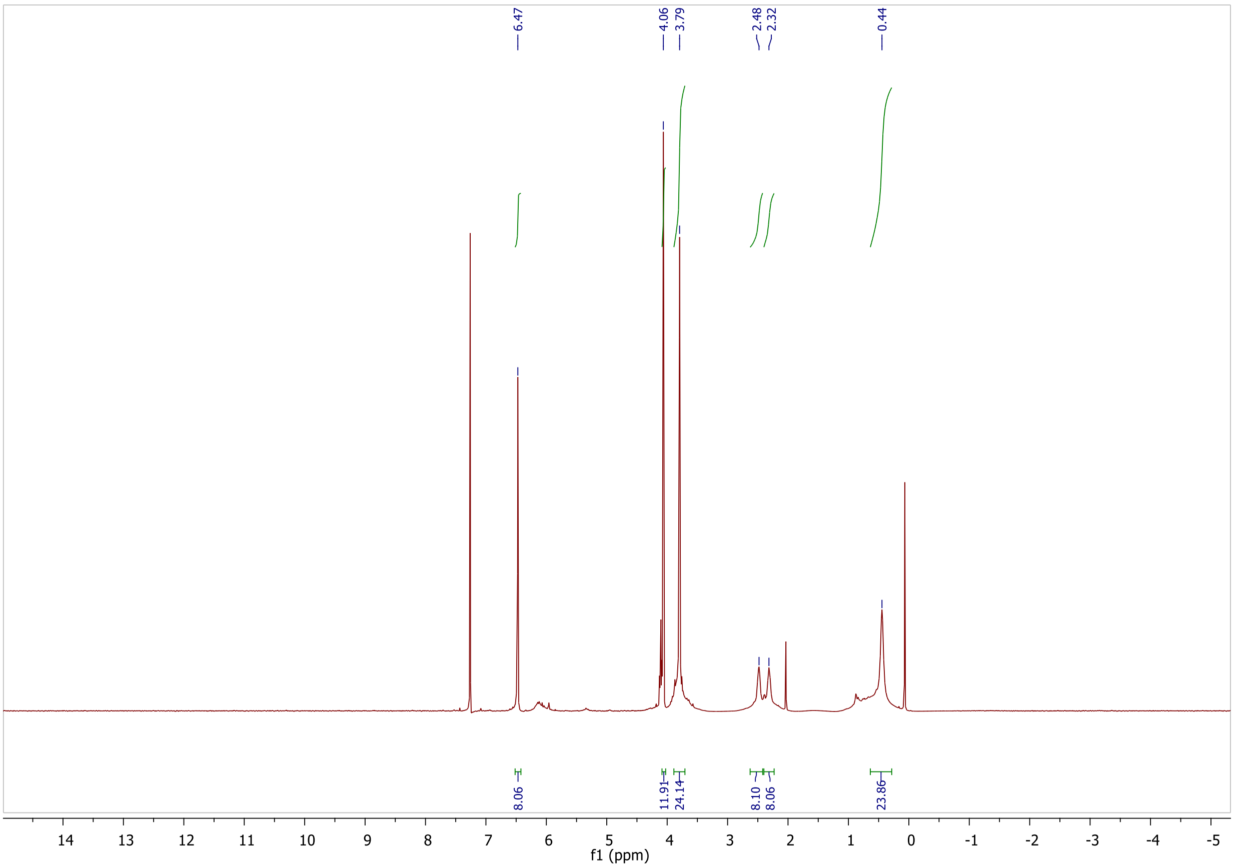

Figure S7. ^{1}H NMR spectrum of **5** (600 MHz, *d*-chloroform, 25 °C).

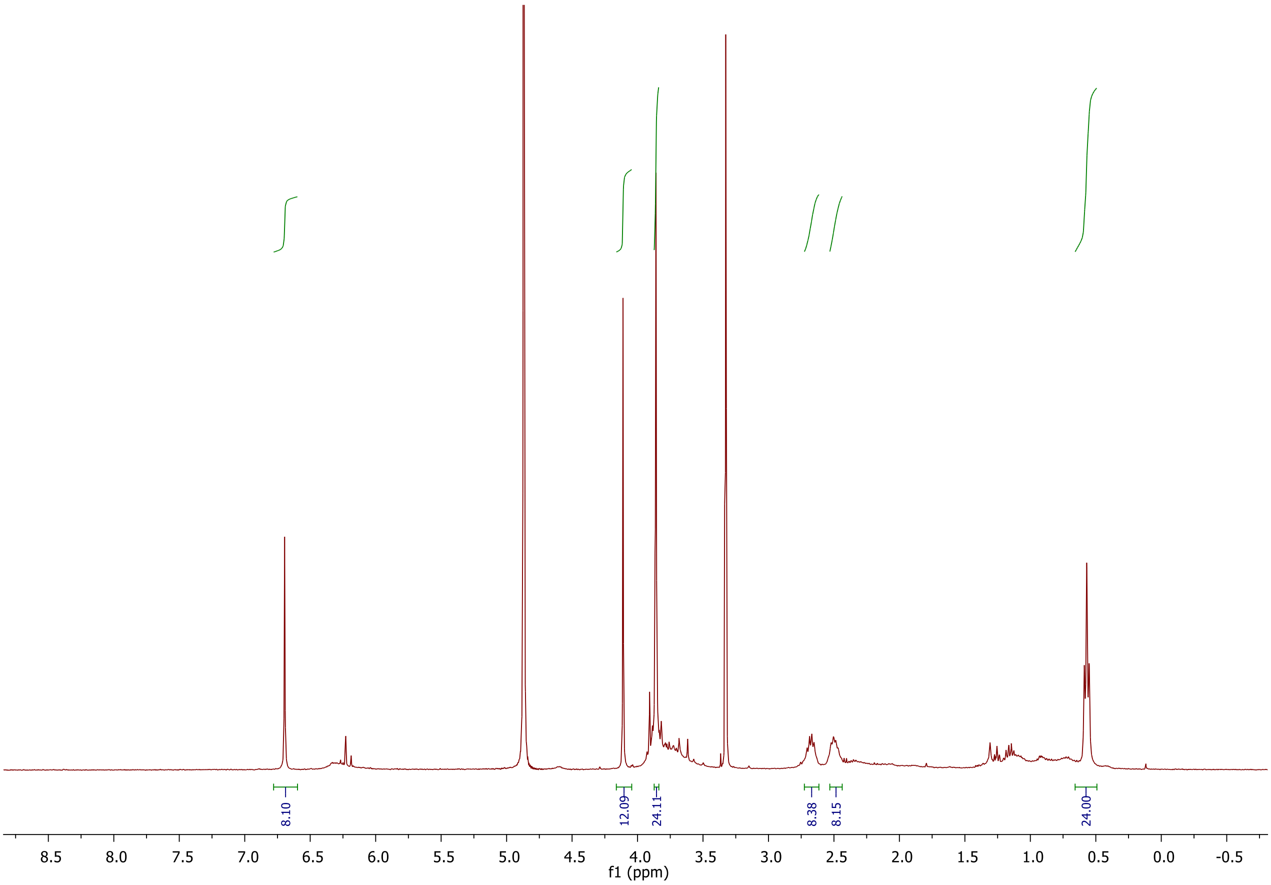

Figure S8. ^{1}H NMR spectrum of **5** (400 MHz, *d*-methanol, 25 °C).

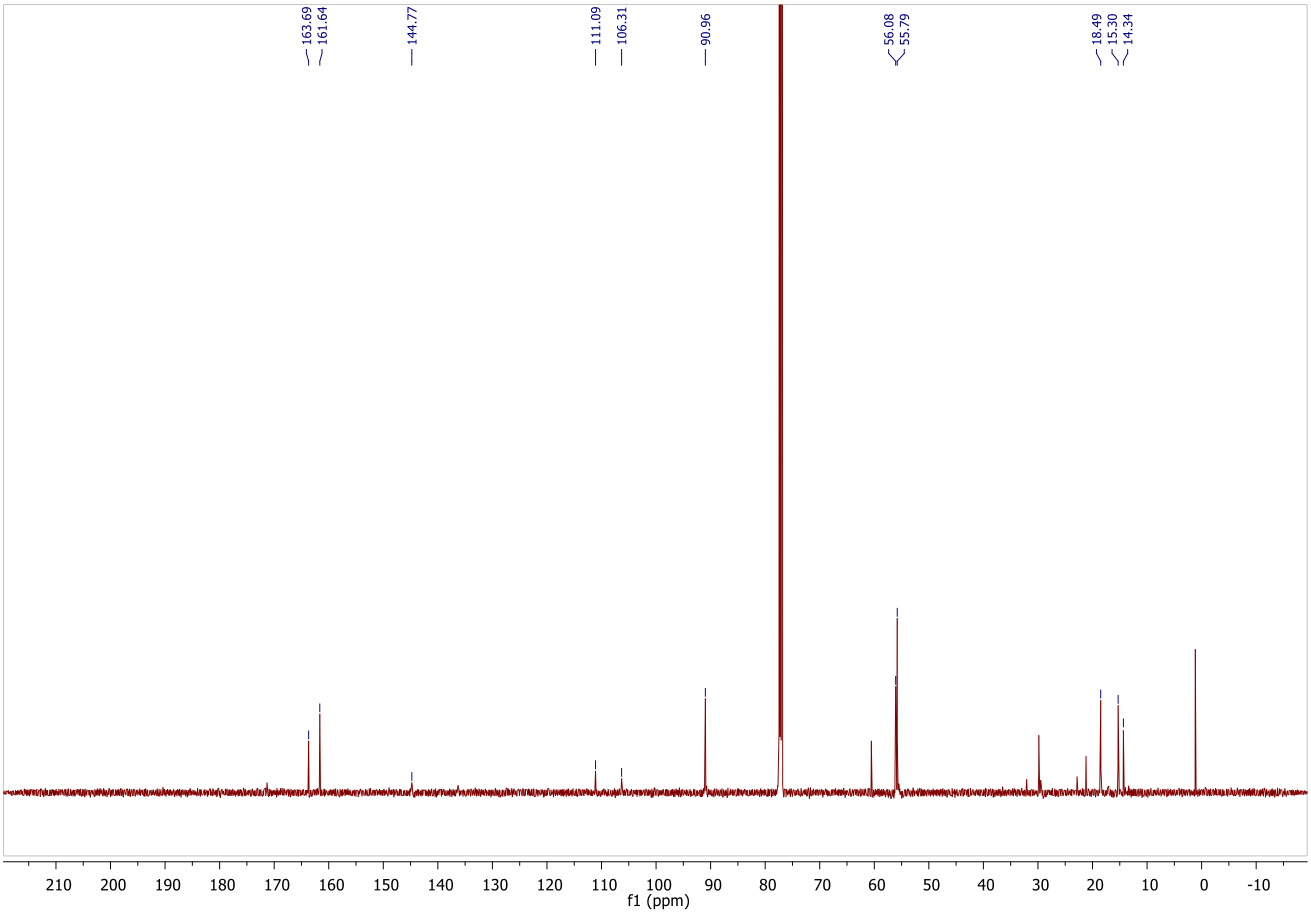

Figure S9. ^{13}C NMR spectrum of **5** (100 MHz, *d*-chloroform, 25 °C).

Minimum:				-1.5			
Maximum:		5.0	20.0	200.0			
Mass	Calc. Mass	mDa	PPM	DBE	i-FIT	i-FIT (Norm)	Formula
1199.6368	1199.6321	4.7	3.9	31.5	55.4	0.0	C72 H87 N4 O12

Figure S10. HRMS (MALDI) of **5**.

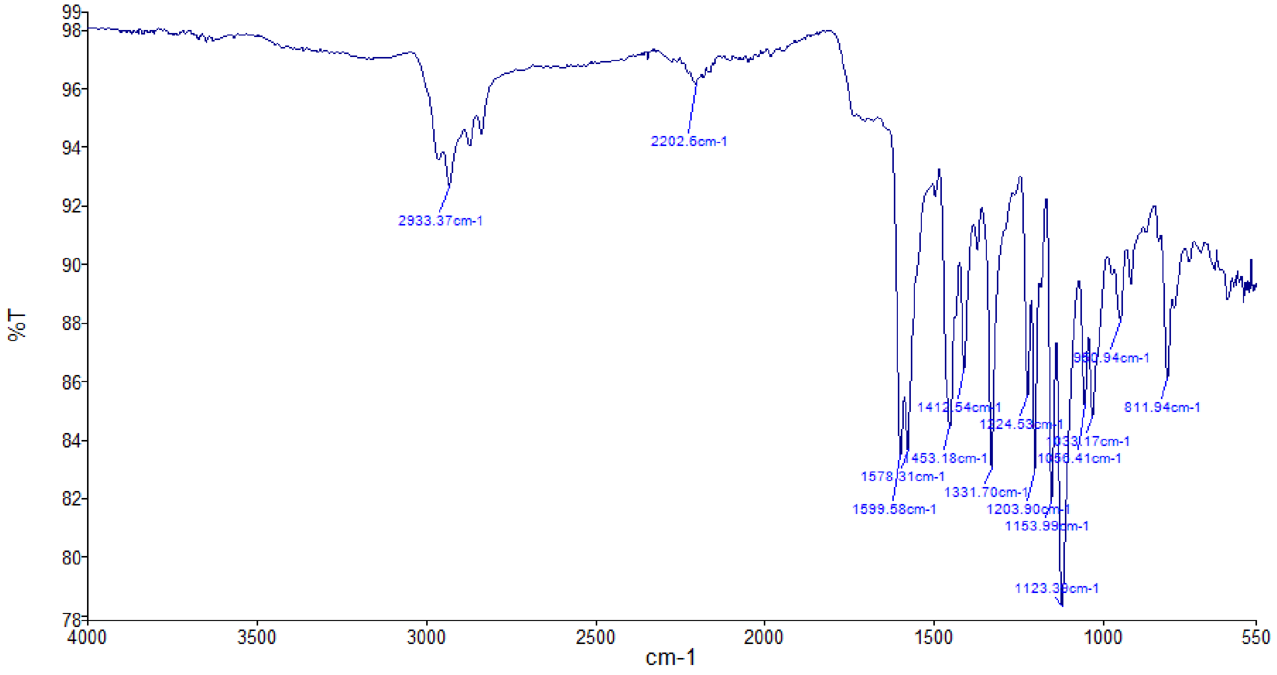

Figure S11. FTIR spectrum of **5**.

Synthesis and characterization of **6**

2,3,7,8,12,13,17,18-Octaethyl-5,10,15,20-tetrakis(2,6-dimethoxy-4-hydroxyphenyl)porphyrin [6]

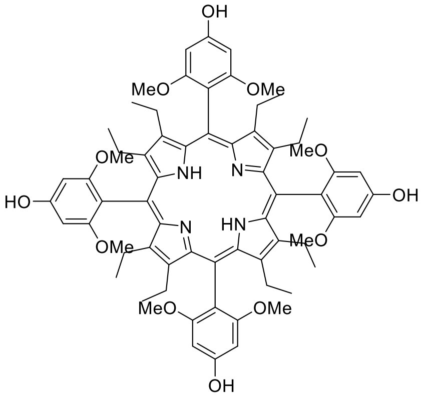

In a predried 50 mL round bottomed flask, 2,3,7,8,12,13,17,18-octaethyl-5,10,15,20-tetrakis(2,4,6-trimethoxyphenyl)porphyrin (**5**) (17.2 mg, 14.34 μmol, 1.0 eq.) was dissolved in dry $CHCl_3$ (4 mL). Under an argon atmosphere the mixture was treated dropwise with BBr_3 (1M in CH_2Cl_2, 0.1 mL, 100 μmol, 7 eq). After 2 h of stirring the reaction at 25 °C, the solvent was removed under vacuum. The reaction mixture was dissolved in DCM/MeOH (9:1) and filtered through a silica gel using DCM/MeOH (9:1) to remove the impurities. The compound was then collected using MeOH, after evaporation of solvent under reduced pressure provided compound **6** as dark green powder [14.1 mg, 12.33 μmol,, 86%]; M.p.: >300 °C; R_f = 0.2 (SiO_2, MeOH); ^{1}H NMR (400 MHz, *d*-methanol, 25 °C) δ= 6.36 (s, 8H, *m*-phenyl-*H*), 3.76 (s, *J* = 13.5 Hz, 24H, *-OCH₃*), 2.84 – 2.52 (m, 16H, *-CH₂*), 0.65 (t, *J* = 7.4 Hz, 24H, -CH_2CH_3).^{13}C NMR (100 MHz, *d*-methanol, 25 °C): δ = 161.79, 160.48, 146.06, 138.17, 108.63, 107.36, 94.87, 55.19, 18.28, 14.71 ppm; UV/Vis (methanol): λ_{max} [nm] (log ε[L·mol^{-1} cm^{-1}]) = 488 (4.97), 695 (4.11); IR (ATR) $\tilde{\nu}$ = 3369.4 (br), 3194.8 (br), 2970, 2935.6, 2876, 2844, 2224.6, 2158, 1595, 1455.1, 1418, 1327.4, 1154, 1118.1, 1056, 1024, 960, 920, 814.48 cm^{-1}

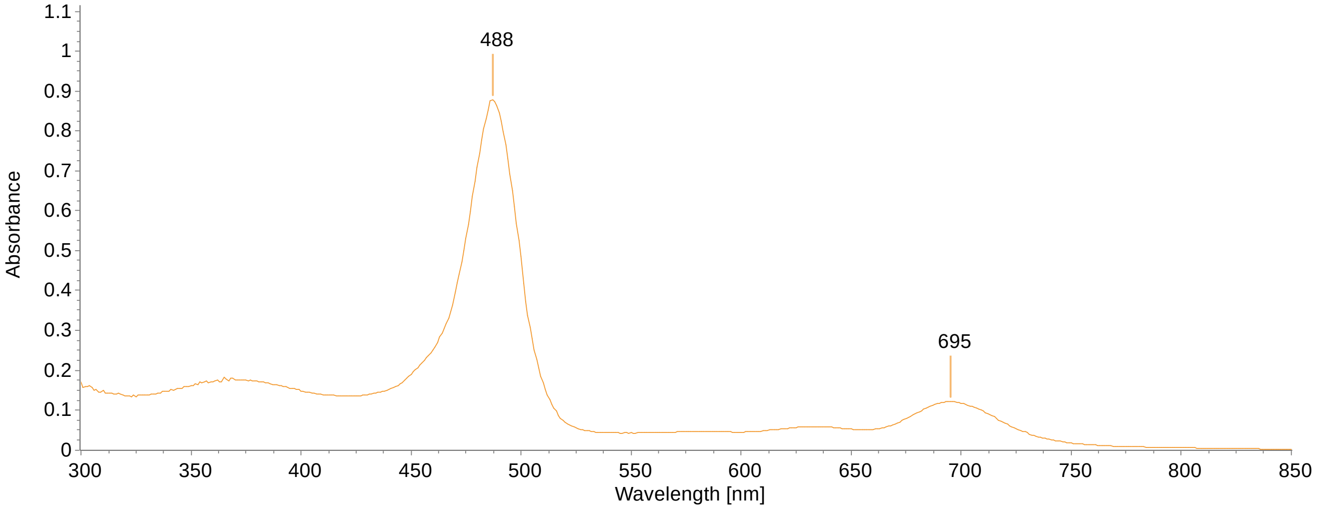

Figure S12. UV-vis spectrum of **6** in methanol.

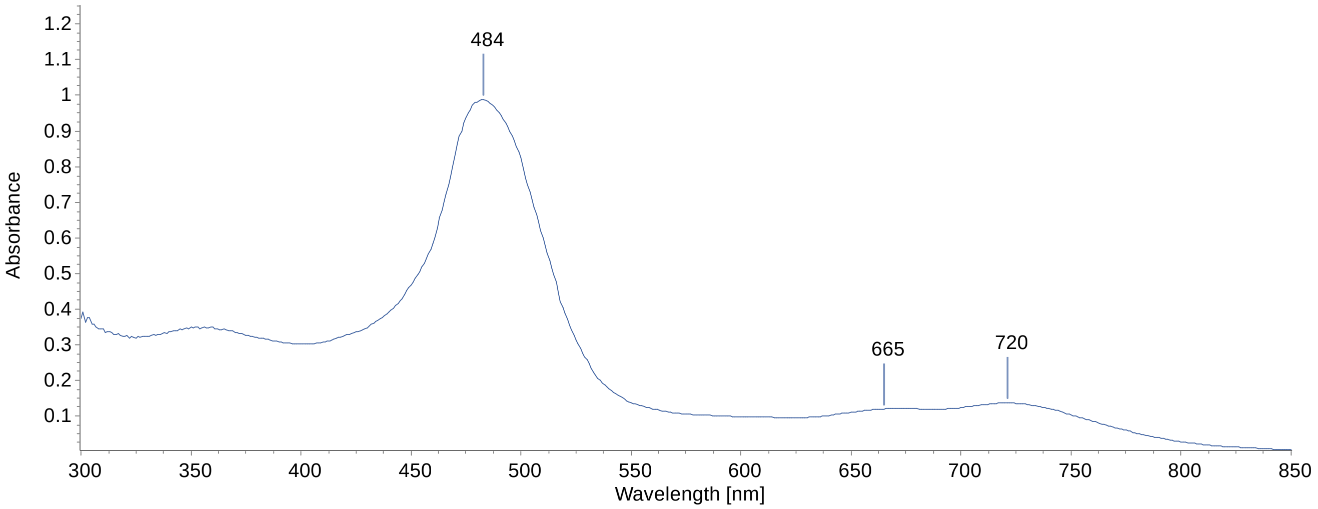

Figure S13. UV-vis spectrum of **6** in methanol with 1% DBU.

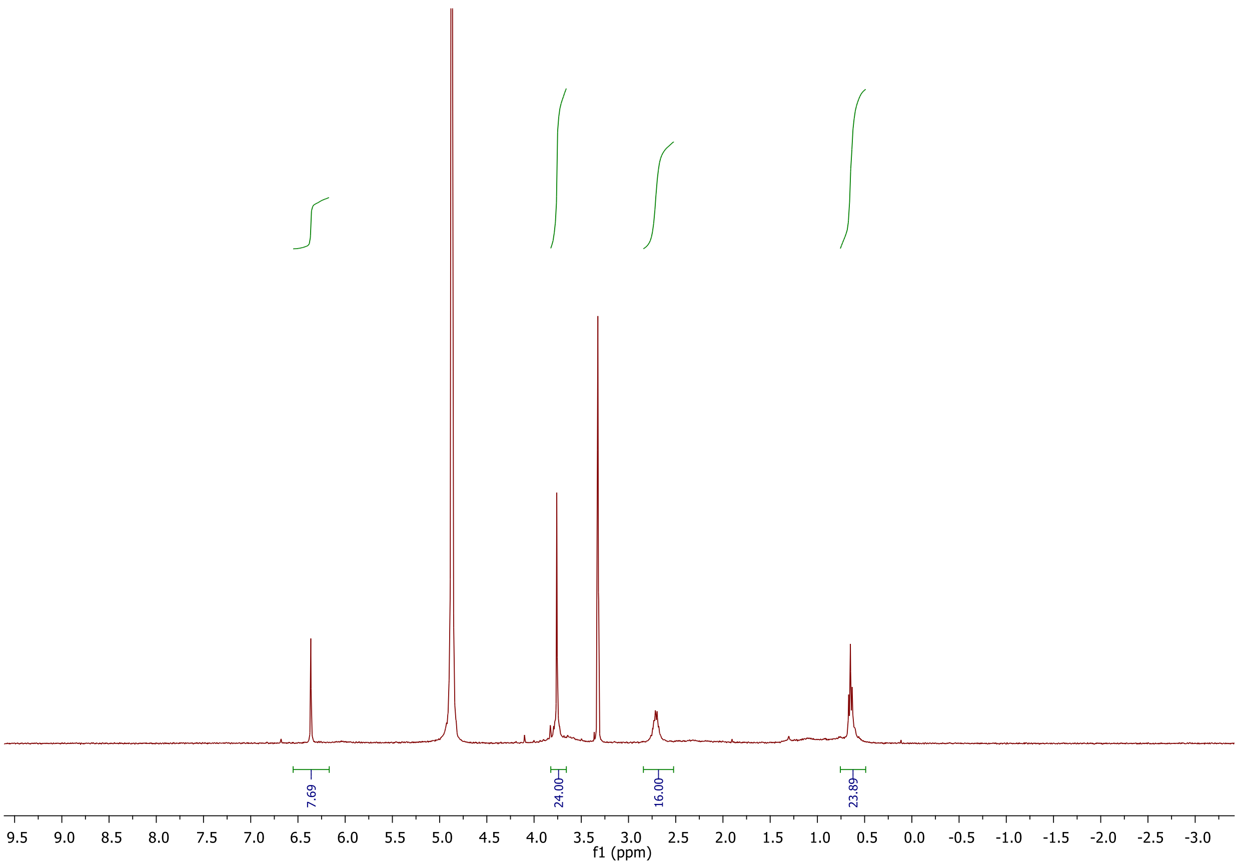

Figure S14. ^{1}H NMR spectrum of **6** (400 MHz, *d*-methanol, 25 °C).

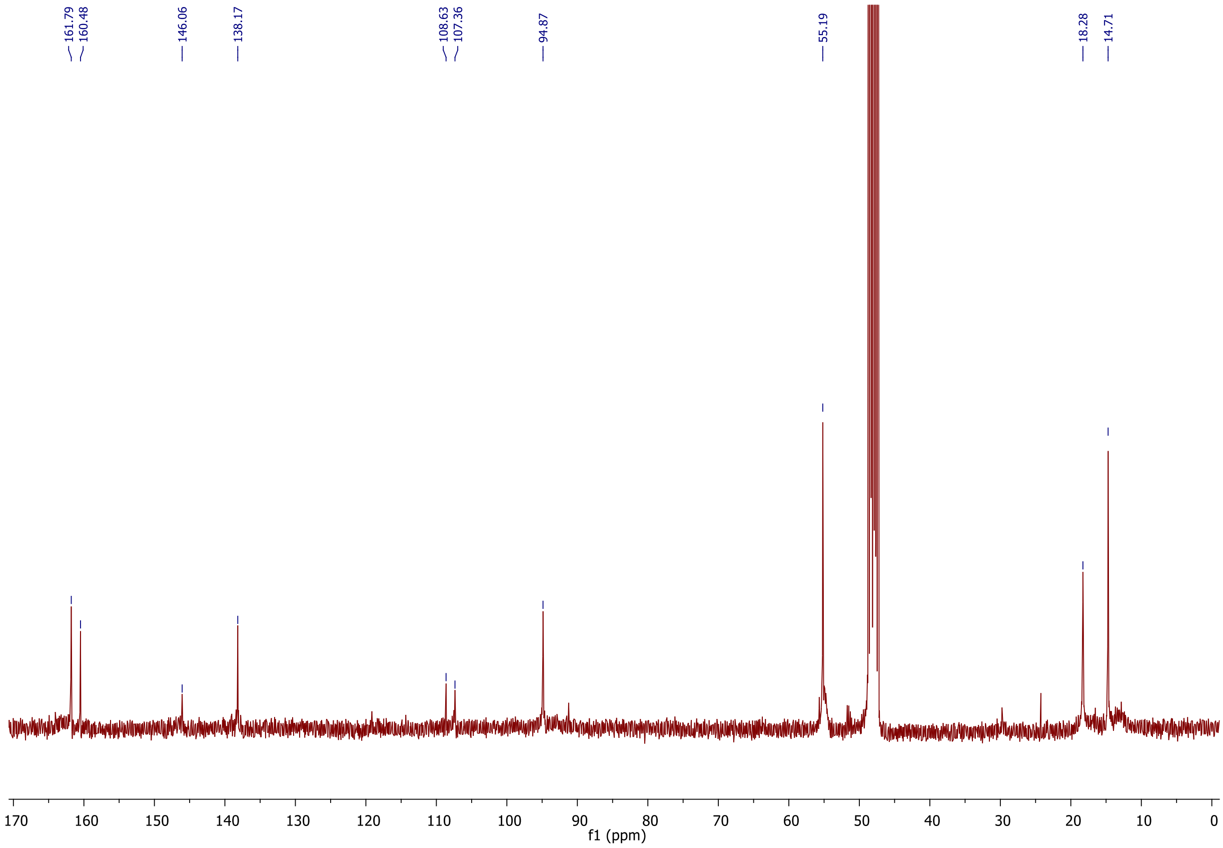

Figure S15. ^{13}C NMR spectrum of **6** (100 MHz, *d*-methanol, 25 °C).

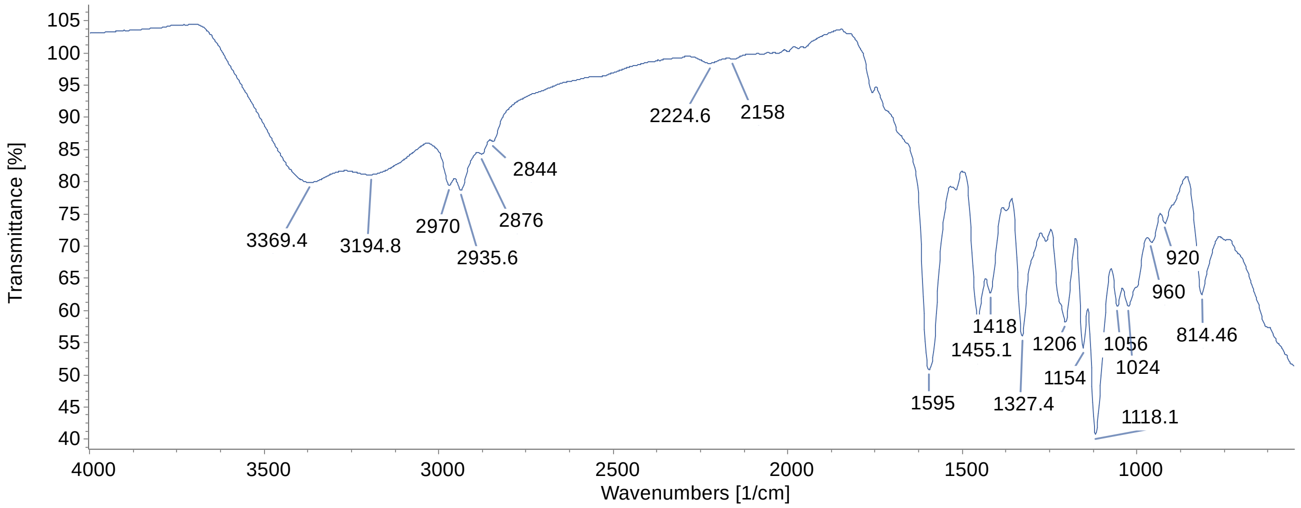

Figure S16. FTIR spectrum of **6**.

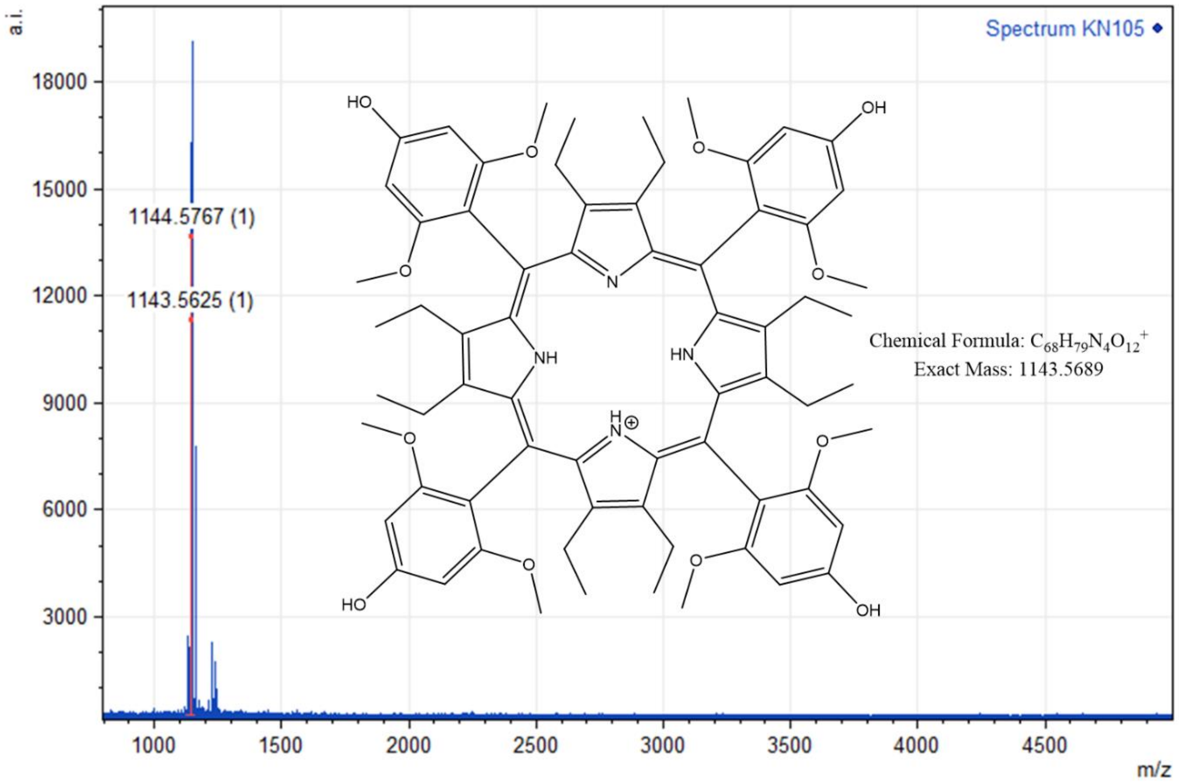

Figure S17. Mass spectrum of **6**, HRMS (MALDI) *m/z* calcd. for $C_{68}H_{79}N_4O_{12}$ $[M+H]^+$: 1143.5689, found 1143.5625, recorded by Bruker Daltonics autoFlex.

Synthesis and characterization of **7**

2,3,7,8,12,13,17,18-Octaethyl-5,10,15,20-tetrakis(3,5-dimethoxyphenyl)porphyrin [7]

Synthesized *via* the General Procedure from 3,5-dimethoxybenzaldehyde **7A** (1.95 g, 11.7 mmol, 1.11 eq.), 3,4-diethyl-1H-pyrrole **11** (1.3 g, 10.55 mmol, 1.00 eq.), $BF_3 \times Et_2O$ (0.01 mL, 0.8 mmol) and DDQ (2.76 g, 12.18 mmol). After aqueous work up, the organic extract was transferred directly to the column (Al_2O_3). Less polar side products and starting material were eluted first (CH_2Cl_2), followed by the collection of the major green band (CH_2Cl_2:MeOH = 50:1 to 150:1 *v/v*). Removal of solvent *in vacuo* yielded the product **3** as a dark purple coloured solid [0.545 g, 0.505 mmol, 19%]; M.p.: 260 °C (decomposition); R_f = 0.25 (SiO_2, Ethyl acetate); ^{1}H NMR (400 MHz, $CDCl_3$, 25 °C) δ= 7.71 (s, 8H, *o*-phenyl-*H*), 6.95 (s, 4H, *p*-phenyl-*H*), 4.07 (s, 24H, -*OCH3*), 2.62 – 2.08 (m, 16H, -*CH2*), 0.30 ppm (t, *J* = 7.3 Hz, 24H, -*CH3*); ^{13}C NMR (100 MHz, $CDCl_3$, 25 °C): δ = 160.26, 143.90, 138.39, 118.74, 115.32, 102.56, 56.25, 18.55, 15.83 ppm; UV/Vis (chloroform): λ_{max} [nm] (log ε[L·mol^{-1} ·cm^{-1}]) = 454.9 (5.28), 552.3 (4.11), 604.4 (3.78), 638.6 (3.62), 697.7 (3.48); HRMS (MALDI) *m/z* calcd. for $C_{68}H_{79}N_4O_8$ $[M+H]^+$: 1079.5909, found 1079.5898. IR (ATR) $\tilde{\nu}$ = 2971.7, 2933.58, 2875.7, 2838.29, 1665.29, 1586.79 (s), 1452.86, 1421.62, 1355.18, 1314.41, 1290.24, 1199.57, 1153.91, 1132.14, 1059.16, 1021.31, 948.20, 835.80, 800.92, 717.10, 687.61 cm^{-1}.

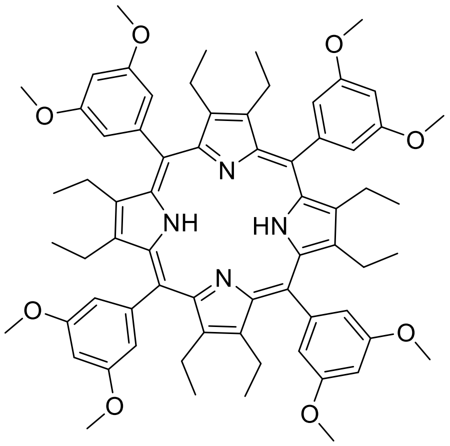

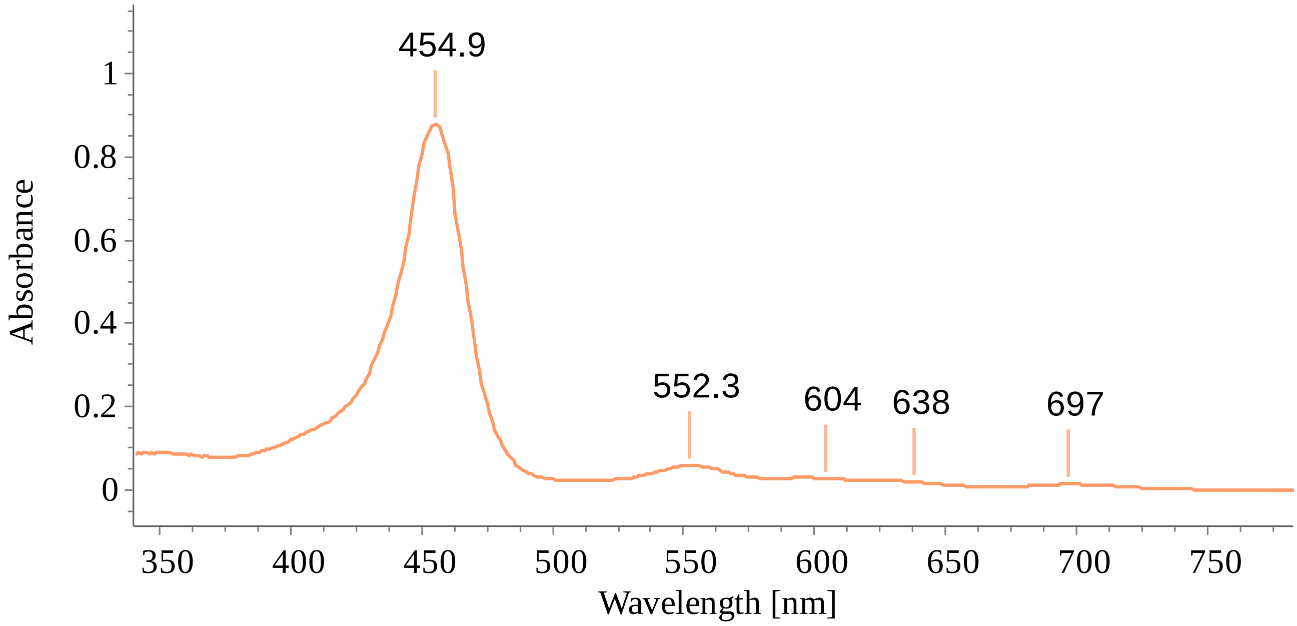

Figure S18. UV-vis spectrum of **7** in chloroform.

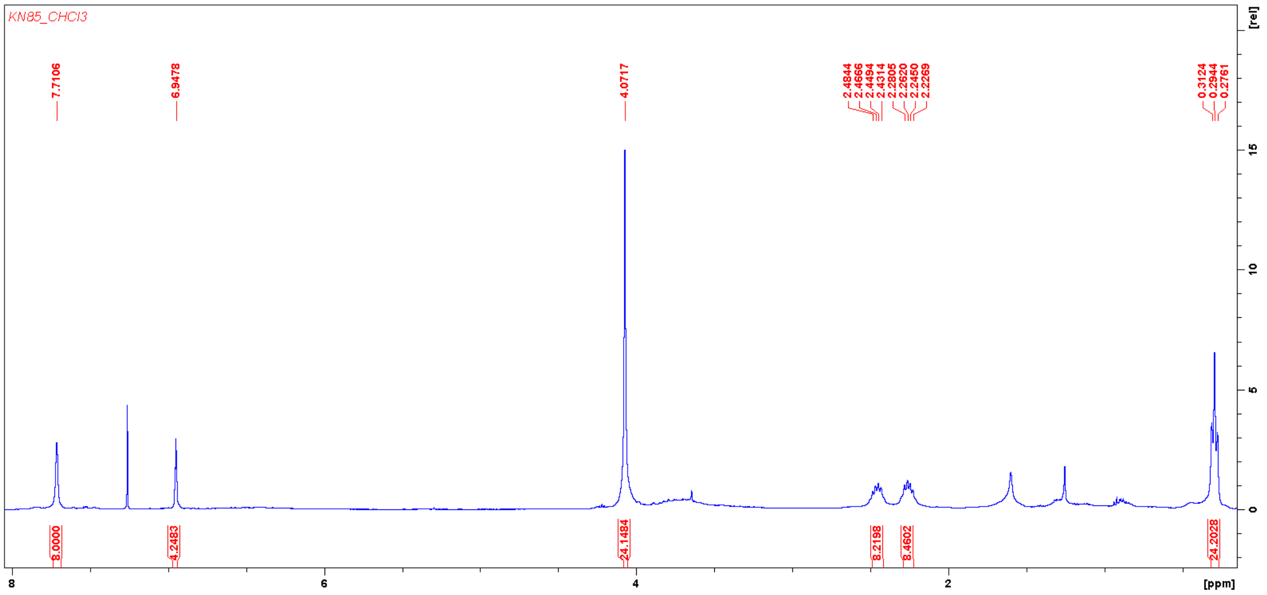

Figure S19. ^{1}H NMR spectrum of **7** (400 MHz, *d*-chloroform, 25 °C).

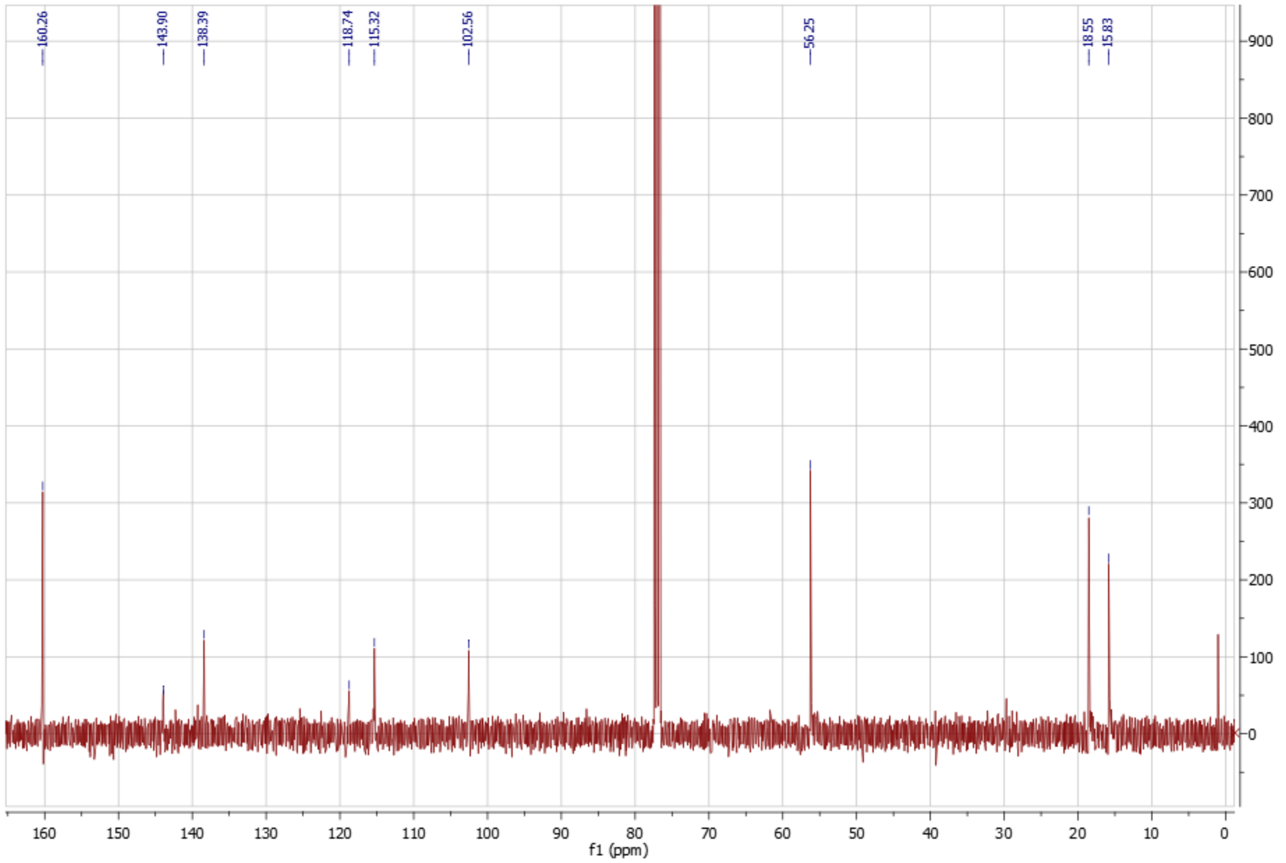

Figure S20. ^{13}C NMR spectrum **7** (100 MHz, *d*-chloroform, 25 °C).

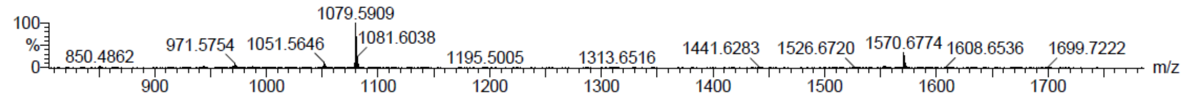

Minimum:				-1.5			
Maximum:		5.0	50.0	200.0			
Mass	Calc. Mass	mDa	PPM	DBE	i-FIT	i-FIT (Norm)	Formula
1079.5909	1079.5898	1.1	1.0	31.5	50.6	0.0	C68 H79 N4 O8

Figure S21. HRMS (MALDI) **7**.

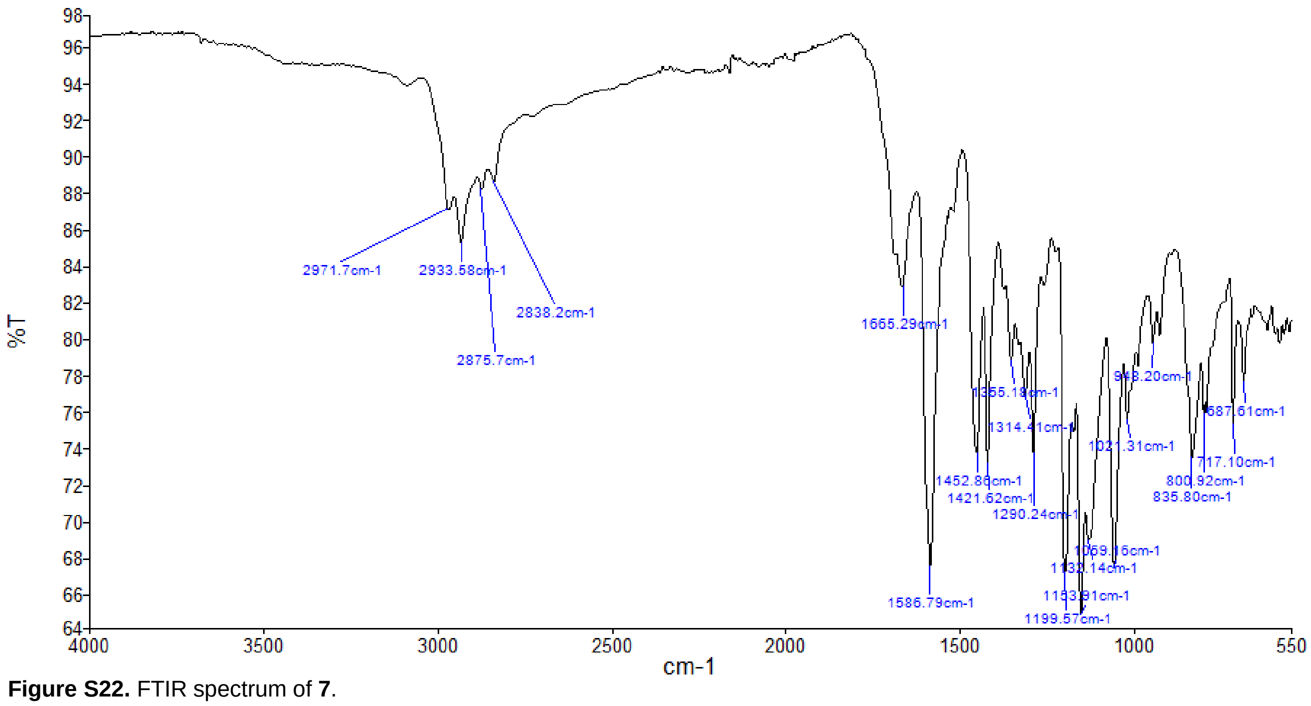

Figure S22. FTIR spectrum of **7**.

Synthesis and characterization of **8**

2,3,7,8,12,13,17,18-Octaethyl-5,10,15,20-tetrakis(3,5-dihydroxyphenyl)porphyrin [8]

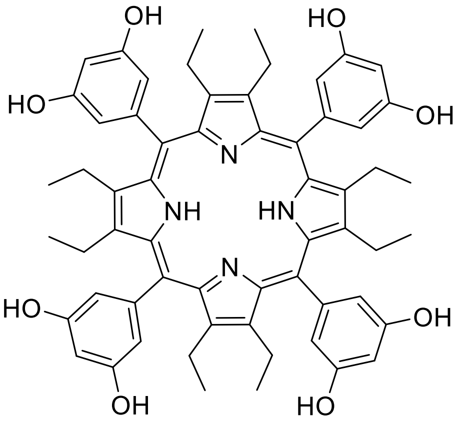

In a predried 100 mL round bottomed flask, 2,3,7,8,12,13,17,18-octaethyl-5,10,15,20-tetrakis(3,5-dimethoxyphenyl)porphyrin **7** (550 mg, 0.506 mmol, 1.0 eq.) was dissolved in dry CH_2Cl_2 (60 mL). Under an argon atmosphere at 0 °C the mixture was treated dropwise with BBr_3 (1M in CH_2Cl_2, 10.5 mL, 10.5 mmol, 16 eq). The reaction flask was warmed to room temperature and after 18 h of stirring, the mixture was added dropwise to ice water (50 mL). NaOH (1 M, 100 mL) was slowly added and washed with dichloromethane (2 x 50 mL). The aqueous phase was acidified with conc. HCl until the product started to coagulate. The green solid was filtered and washed with water and dichloromethane. The green solid was dissolved with methanol and after evaporation of solvent under reduced pressure provided a dark green crystalline solid **8**. [519 mg, 0.499 mol, 76%]; M.p.: >300 °C; R_f = 0.63 (SiO_2, MeOH); ^{1}H NMR (400 MHz, d-methanol, 25 °C) δ= 7.40 (s, 8H, *o*-phenyl-*H*), 6.83 (s, 4H, *p*-phenyl-*H*), 2.67–2.42 (m, 16H, -*CH*$_2$), 0.52 ppm (t, *J* = 7.4 Hz, 24H, -CH_2*CH*$_3$); ^{13}C NMR (100 MHz, d-methanol, 25 °C): δ = 158.41, 143.60, 139.10, 138.95, 118.60, 115.86, 104.08, 18.19, 14.96 ppm; UV/Vis (Methanol): λ_{max} [nm] (log ε[L·mol^{-1} cm^{-1}]) = 475.9 (5.36), 689.2 (4.45); HRMS (MALDI) *m/z* calcd. for $C_{60}H_{63}N_4O_8$ [M+H]$^+$: 967.4617, found 967.4646. IR (ATR) $\tilde{\nu}$ = 3148.18 (br), 2972.31, 2933.66, 2873, 1587.88 (s), 1502.13, 1439.13, 1354.45, 1292.03, 1199.71, 1152.15 (s), 1054.38, 1003.66, 994.74, 980.09, 951.42, 886.88, 847.24, 809.12, 777.76, 685.77 cm^{-1}

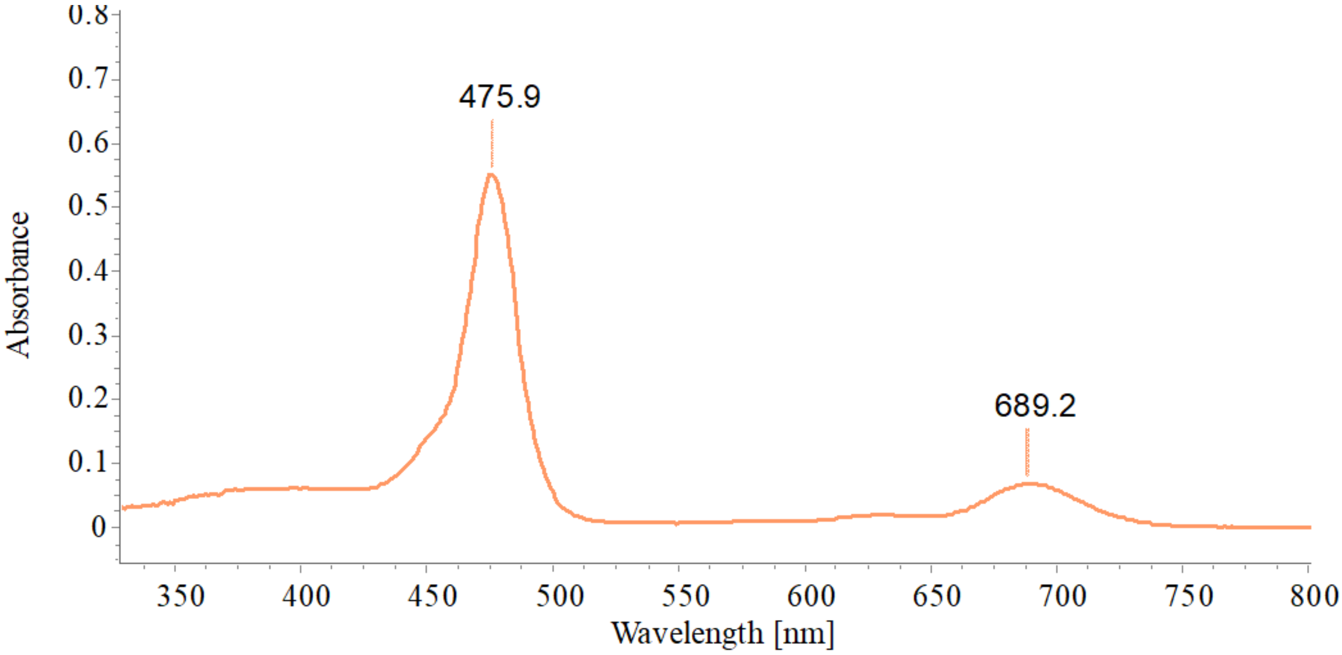

Figure S23. UV-vis spectrum of **8** in methanol. Note, the porphyrin was isolated protonated as Cl salt.

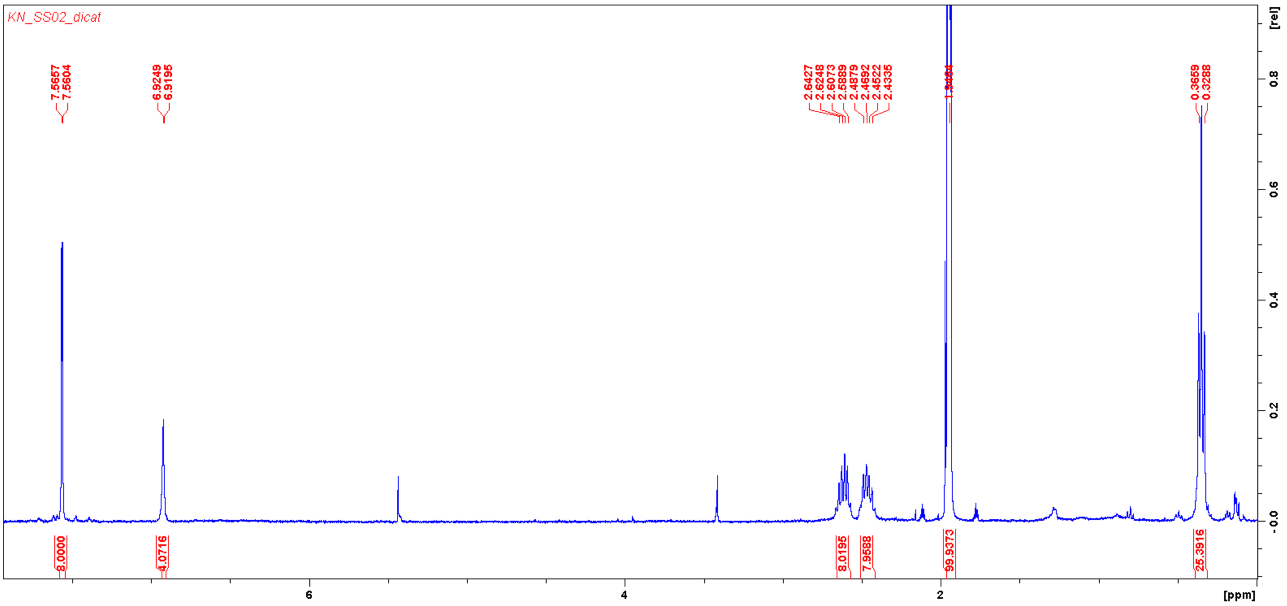

Figure S24. ^{1}H NMR spectrum of **8** (400 MHz, CD_3OD, 25 °C).

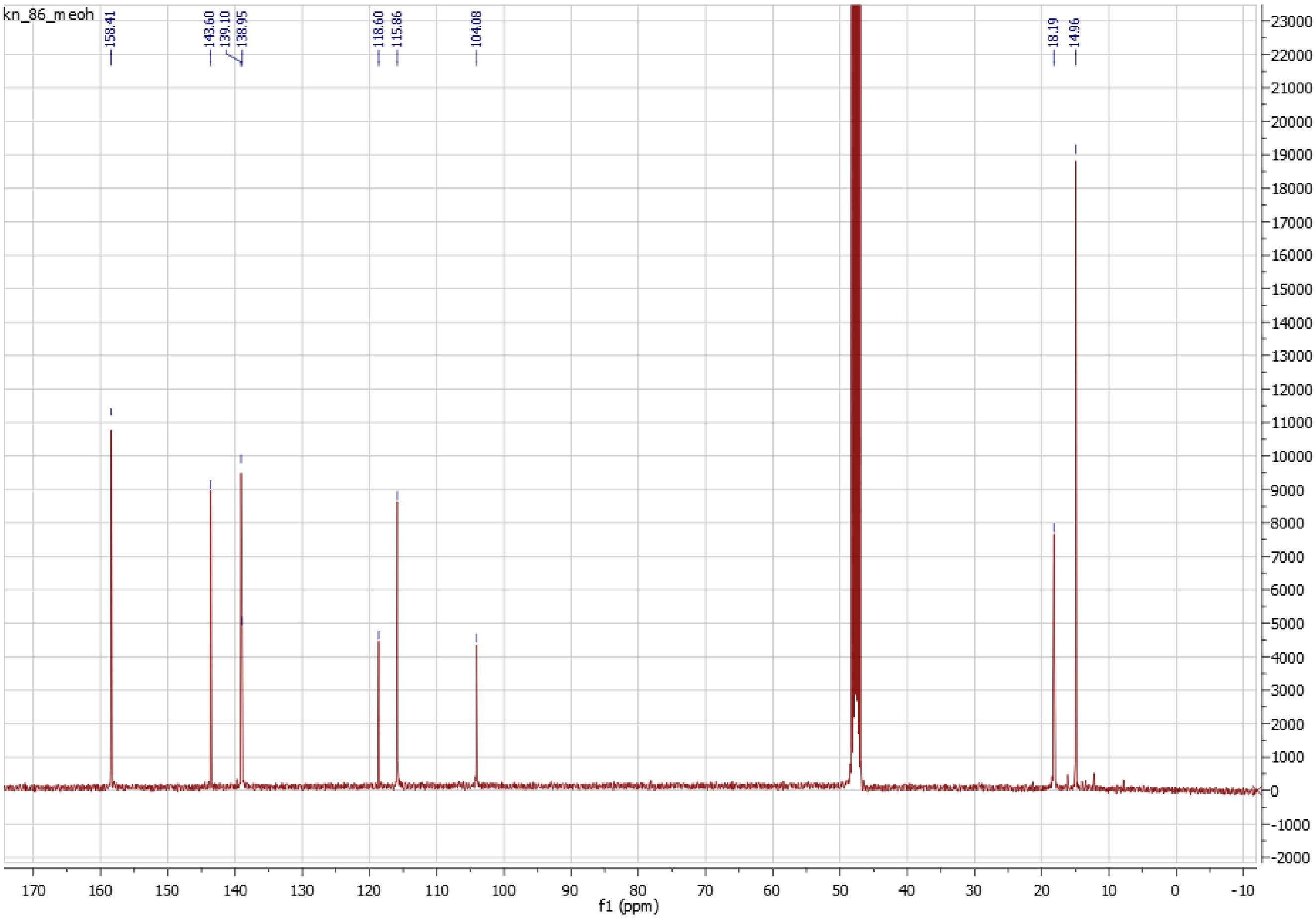

Figure S25. ^{13}C NMR spectrum of **8** (400 MHz, CD_3OD, 25 °C).

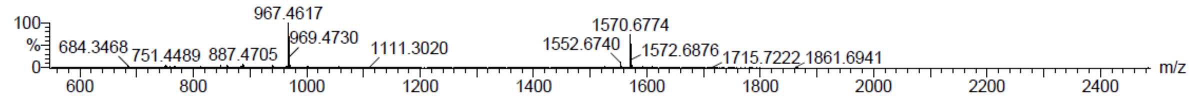

	Mass	Calc. Mass	mDa	PPM	DBE	i-FIT	i-FIT (Norm)	Formula
Minimum:					-1.5			
Maximum:			5.0	10.0	200.0			
	967.4617	967.4646	-2.9	-3.0	31.5	38.2	0.0	C60 H63 N4 O8

Figure S26. HRMS (MALDI) of **8**.

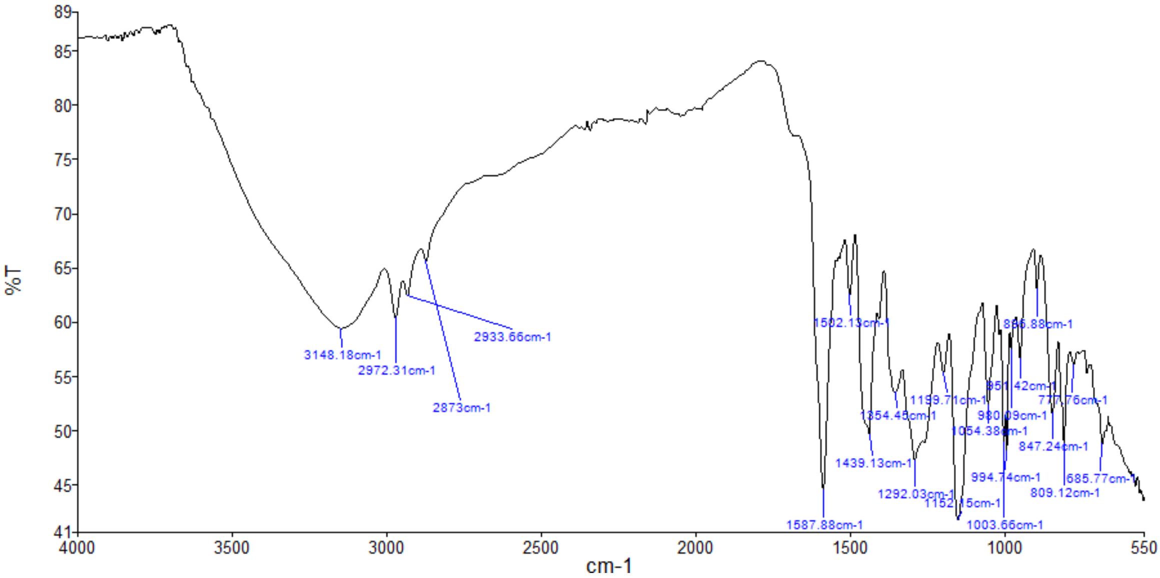

Figure S27. FTIR spectum of **8.**

Synthesis and characterization of **9A**

3,5-Dipivaloyloxybenzaldehyde [9A]

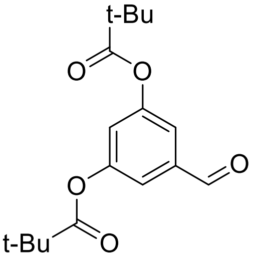

In a 250 mL RBF at room temperature, 3,5-dihydroxybenzaldehyde (0.5 g, 3.62 mmol, 1.0 eq.) was dissolved in acetonitrile (100 mL). *N,N*-diisopropylethylamine (5 mL, 28.96 mmol, 8.0 eq) was added. The reaction was treated dropwise with pivaloyl chloride (1.75 mL, mol, eq) and stirred for 1 h. The crude mixture was extracted with dichloromethane, washed with $NaHCO_3$ (1 × 150 mL), brine (1 × 150 mL) and water (1 × 150 mL). The organic phase was dried with anhydrous magnesium sulfate, filtered and evaporated *in vacuo* to yield a white crystalline solid **9A**. [1.069 g, 3.49 mmol, 96%]; M.p.: 146.5 °C; R_f = 0.89 (SiO_2, EtOAc/*n*-hexane 1:1 v/v); ^{1}H NMR (400 MHz, $CDCl_3$, 25 °C) δ= 9.99 (s, 1H, CO*H*), 7.51 (d, J_m= 2.17 Hz, 2H, *o*-phenyl-*H*), 7.17 (t, J_m= 2.1 Hz, 1H, *p*-phenyl-*H*), 1.39 ppm (s, 18H, -*CH*$_3$); ^{13}C NMR (100 MHz, $CDCl_3$, 25 °C): δ = 190.29, 176.47, 152.09, 138.00, 121.50, 119.79, 39.22, 27.06, 26.53 ppm; HRMS (APCI) *m/z* calcd. for $C_{17}H_{23}O_5$ $[M]^+$: 307.1535, found 307.1540; IR (ATR) $\tilde{\nu}$ = 2974.91, 2937.33, 2874.84, 1752.35 (CO stretch), 1697.34, 1592.1, 1480.72, 1396.93, 1368.58, 1300.07, 1269.26, 1091.23 (s, br), 1030.07 (sh), 980.16, 941.15, 905.88, 803.73, 803.41, 757.89, 732.27, 672.11, 597.75, 552.37 cm^{-1}.

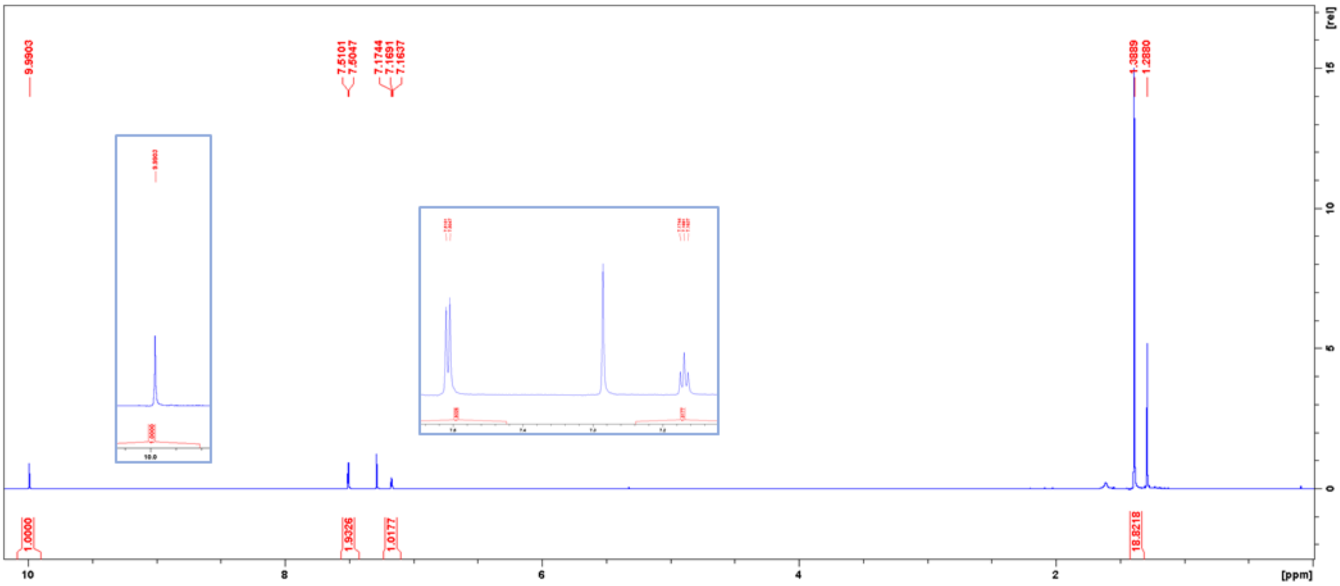

Figure S28. ^{1}H NMR spectrum of 9A (400 MHz, *d*-chloroform, 25 °C).

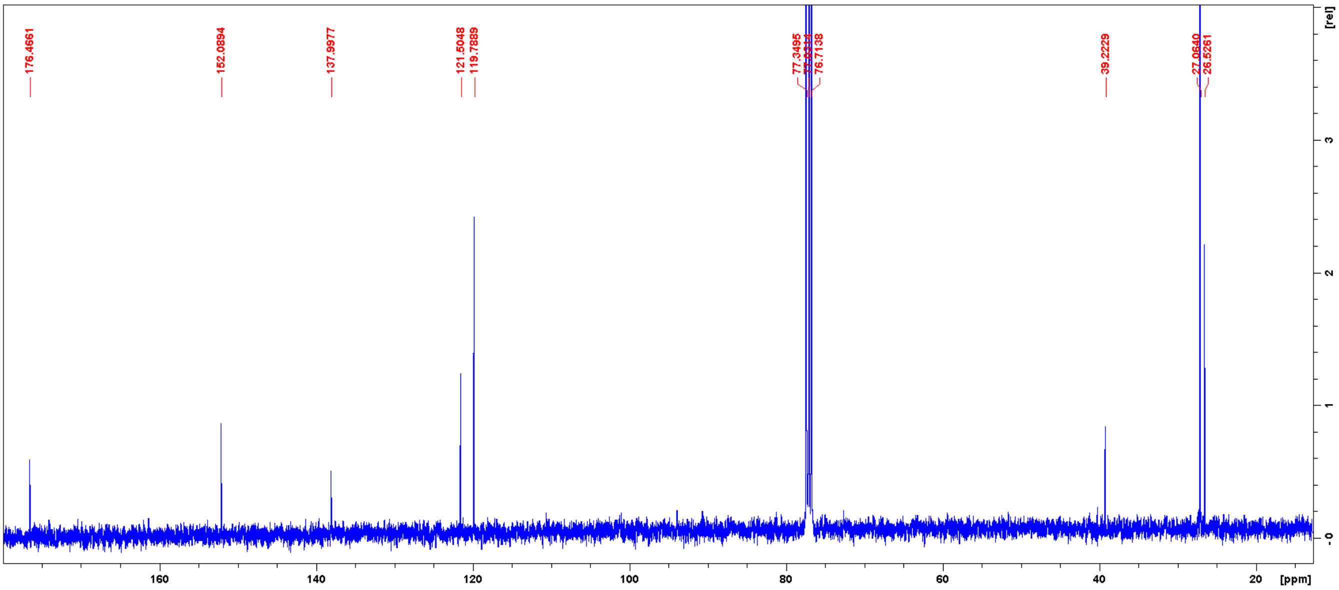

Figure S29. ^{13}C NMR spectrum of **9A** (101 MHz, *d*-chloroform, 25 °C).

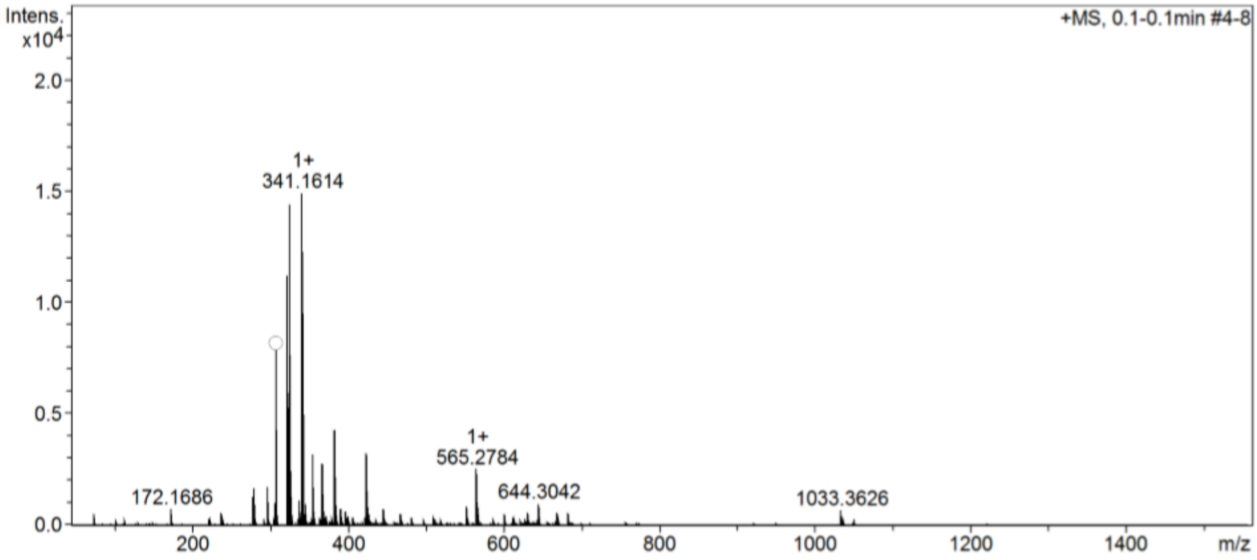

Meas. m/z	#	Ion Formula	m/z	err [mDa]	err [ppm]	rdb	N-Rule	e⁻ Conf	mSigma
307.153519	1	C17H23O5	307.154000	0.5	1.6	6.5	ok	even	10.6

Figure S30. HRMS (APCI) of **9A**.

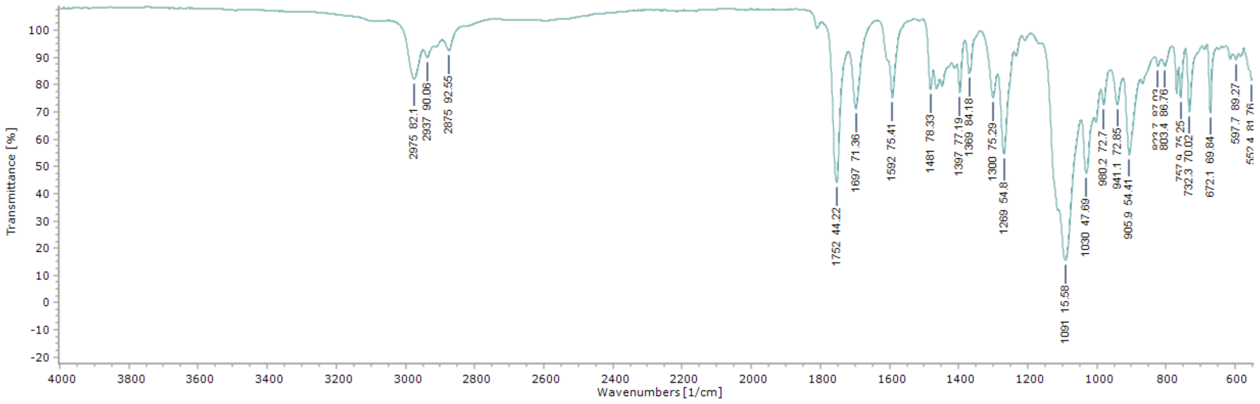

Figure S31. FTIR spectrum of **9A**.

Synthesis and characterization of **9**

2,3,7,8,12,13,17,18-Octaethyl-5,10,15,20-tetrakis(3,5-dipivaloyloxyphenyl)porphyrin [9]

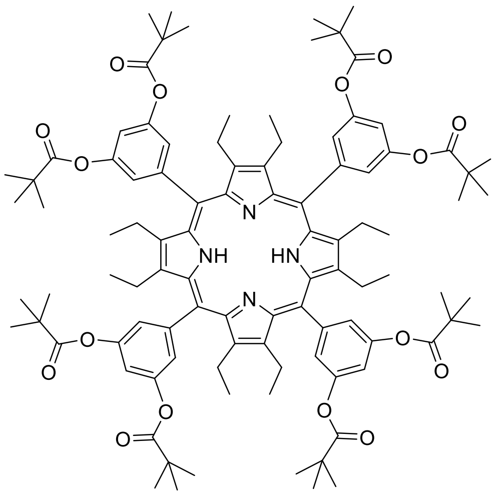

A: In a pre-dried 50 mL Schleck flask precursor porphyrin **8** (46 mg, 0.048 mmol, 1 eq.) was stirred in THF (8 mL) with pivaloyl chloride (0.53 mL, 3 mmol, 64 eq.) in the presence of *N,N*-diisopropylamine (0.374 mL, 3 mmol, 64 eq.) for 5 h. Aqueous work up with saturated $NaHCO_3$ (2 x 50 mL) and water (50 mL) followed by evaporation of solvent *in vacuo* yielded product **9** as green crystalline solid [52 mg, 0.032 mmol, 67%]. Introduction of eight nonpolar *tert*-butyl groups was easily monitored by TLC due to the large difference in R_f values between the highly polar starting material and product.

B: Synthesized *via* the General Procedure using 3,5-dipivaloyloxybenzaldehyde **9A** (1.069 g, 3.49 mmol, 1.0 eq.), 3,4-diethylpyrrole **11** (481 mg, 3.90 mmol, 1.1 eq.), BF_3×Et_2O (0.05 mL, 4.46 mmol) and DDQ (1.2 g, 5.29 mmol) in CH_2Cl_2 (500 mL). After aqueous work up, the residue was transferred directly to a silica gel column and non-polar impurities eluted with CH_2Cl_2. The crude mixture was acidified with 1% TFA and product was collected *via* silica gel column (CH_2Cl_2/EtOAc 4:1 v/v). Aqueous work up with NaOH (1 M, 2 x 150 mL) and water (150 mL) followed by evaporation of solvent *in vacuo* yielded the neutralized product as green crystalline solid. This was recrystallized by slow diffusion in DMSO/acetonitrile to yield dark green crystals of **9** [0.809 g, 0.493 mmol, 57%]; M.p.: 276 °C (decomposition); R_f = 0.72 (SiO_2, *n*-hexane/EtOAc 1:1 v/v); ^{1}H NMR (600 MHz, $CDCl_3$, 25 °C) δ= 8.17 (s, 8H, *o*-phenyl-*H*), 7.38 (s, 4H, *p*-phenyl-*H*), 2.56–2.30 (m, 16H, -CH_2), 1.51 (s, 72H, *t*-Bu), 0.42 ppm (t, *J*= 7.4 Hz, 24H, -CH_2CH_3); ^{13}C NMR (100 MHz, $CDCl_3$, 25 °C): δ = 176.77, 151.21, 144.00, 139.30, 138.71, 127.20, 116.79, 116.52, 39.33, 27.24, 18.71, 15.73 ppm; UV/Vis (chloroform): λ_{max} [nm] (log ε[L·mol^{-1} ·cm^{-1}]) = 456.3 (5.17), 553.4 (4.10), 600.4 (3.48), 631.0 (3.62), 702.1 (3.62); HRMS (MALDI) *m/z* calcd. for $C_{100}H_{127}N_4O_{16}$ [M+H]$^+$: 1639.9247, found 1639.9216; IR (ATR) $\tilde{\nu}$ = 2970.96, 2934.29, 2873.32, 1754.12 (s, CO stretch) 1605.2, 1587.04, 1479.79, 1458.96, 1428.24, 1397.00, 1367.91 1268.48 1163.57, 1090.14 (s, br), 1028.82 (sh), 978.72, 945.95, 904.07, 862.88, 822.38, 798.92, 688.04, 617.29, 559.51 cm^{-1}.

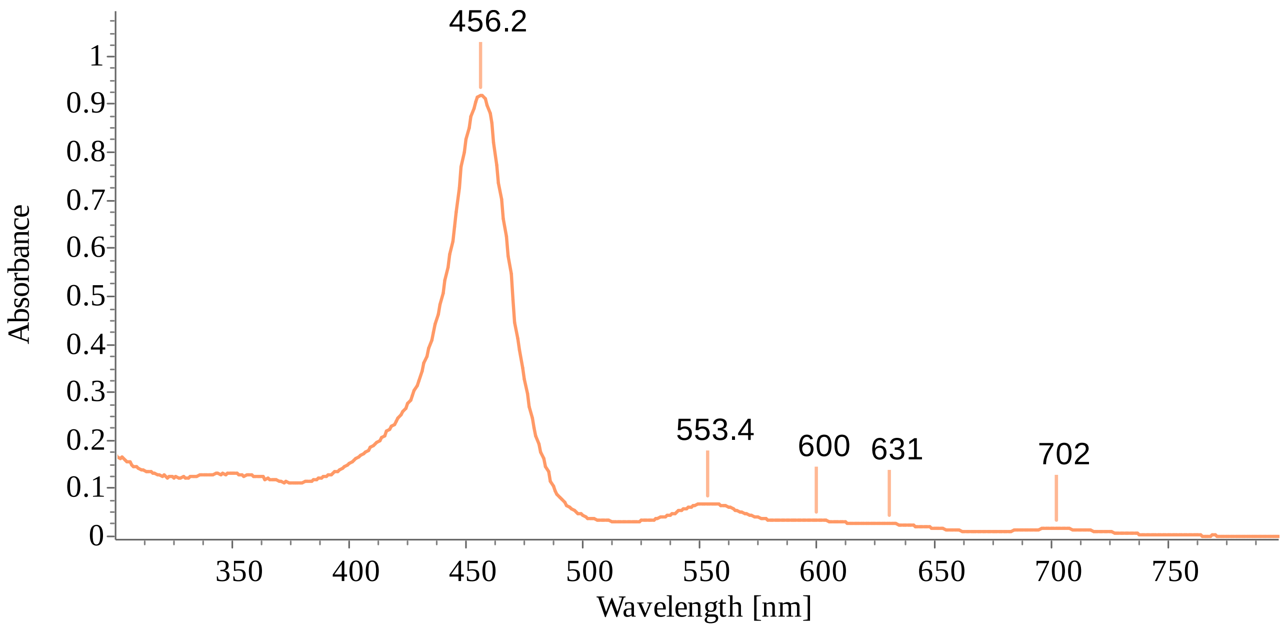

Figure S32. UV-vis spectrum of **9** in chloroform.

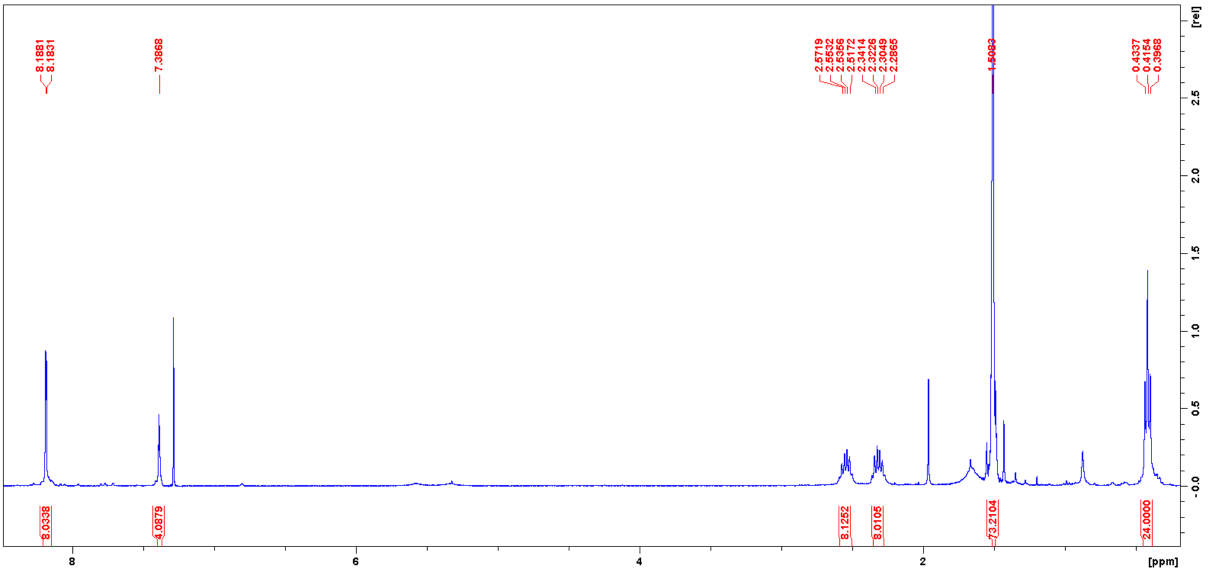

Figure S33. ^{1}H NMR spectrum of **9** (600 MHz, *d*-chloroform, 25 °C).

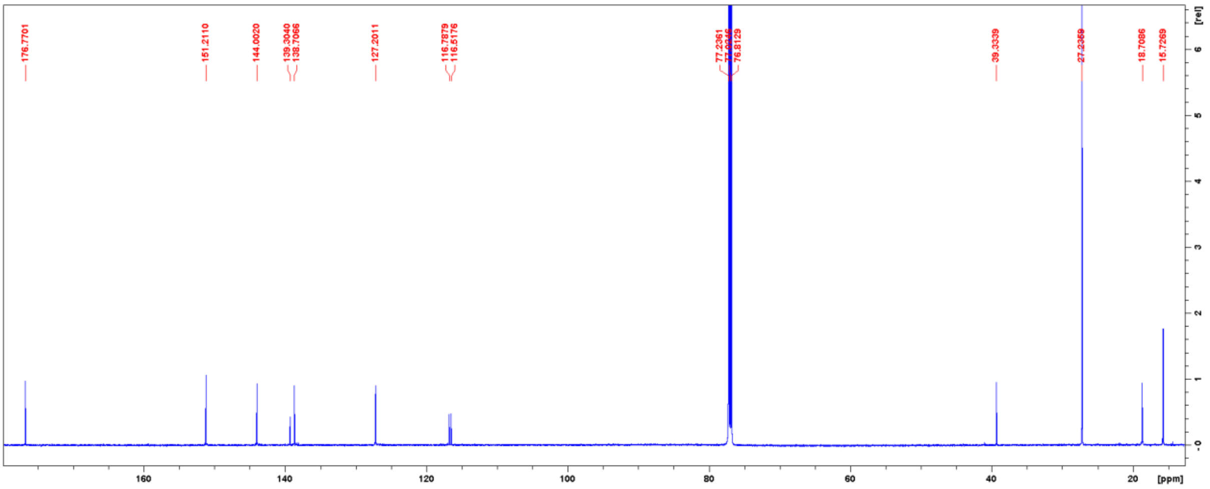

Figure S34. ^{13}C NMR spectrum of **9** (101 MHz, *d*-chloroform, 25 °C).

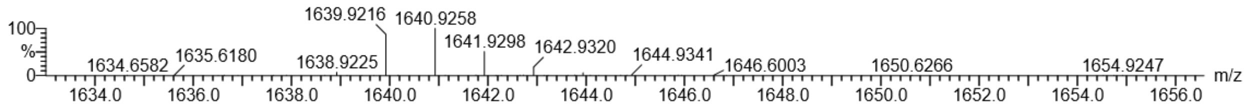

Minimum:				-1.5			
Maximum:		5.0	50.0	400.0			
Mass	Calc. Mass	mDa	PPM	DBE	i-FIT	i-FIT (Norm)	Formula
1639.9216	1639.9247	-3.1	-1.9	39.5	34.4	0.0	C100 H127 N4 O16

Figure S35. HRMS (MALDI) of **9**.

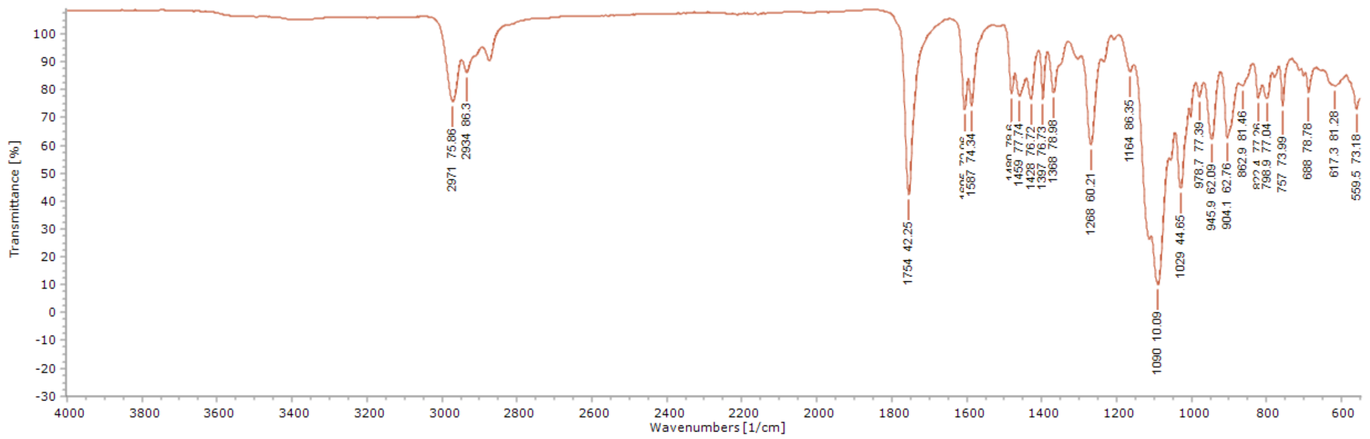

Figure S36. FTIR spectrum of **9**.

Synthesis and characterization of **10A**

5-Pivaloyloxy-2-nitrobenzaldehyde [10A]

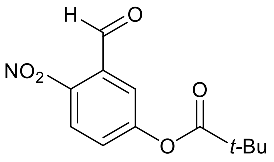

In a 250 mL RBF at room temperature, 5-hydroxy-2-nitrobenzaldehyde (0.500 g, 2.99 mmol, 1.0 eq.) was dissolved in acetonitrile (120 mL). *N,N*-Diisopropylethylamine (5 mL, 28.96 mmol, 8.0 eq) was added. The reaction was treated dropwise with pivaloyl chloride (1.46 mL, 11.98 mmol, 4 eq.) and stirred for 1h. The crude mixture was extracted with dichloromethane, washed with $NaHCO_3$ (1 × 150 mL), brine (1 × 150 mL) and water (1 × 150 mL). The organic phase was dried with anhydrous magnesium sulfate, filtered and concentrated *in vacuo* to yield a crystalline solid. Purification by silica gel column (CH_2Cl_2) collected a pale yellow fraction, which was dried to afford the product **10A** as a white crystalline solid [0.359 g, 1.43 mmol, 75 %]; M.p.: 92 - 96 °C; R_f = 0.64 (SiO_2, CH_2Cl_2); ^{1}H NMR (400 MHz, $CDCl_3$, 25 °C) δ= 10.47 (s, 1H, CO*H*), 8.22 (d, J_o= 8.9 Hz, 1H, *m*-phenyl-*H*), 7.65 (d, J_m= 2.4 Hz, 1H, *o*-phenyl-*H*), 7.49 (dd, J_o= 9.0 Hz, J_m = 2.8 Hz, 1H, *p*-phenyl-*H*), 1.40 ppm (s, 9H, *t*-Bu); ^{13}C NMR (100 MHz, $CDCl_3$, 25 °C): *δ*= 187.30, 173.91, 155.38, 146.25, 133.23, 126.50, 122.63, 39.40, 26.98 ppm; HRMS (APCI) *m/z* calcd. for $C_{12}H_{12}NO_5$ $[M]^-$: 250.0721, found 250.0718; IR (ATR) $\tilde{\nu}$ = 2973.75, 2873.12, 1757.43 (s, C=O stretch.), 1610.98, 1578.45, 1525.08 (s, NO asym. stretch.), 1465.06, 1397.46, 1340.72 (s, NO sym. stretch), 1260.34, 1182.77, 1093.08 (s, C-O stretch.), 1023.02 (sh, C-O stretch.), 951.08, 900.54, 850.25, 751.93, 696.63, 652.16, 622.27, 574.91, 539.58 cm^{-1}.

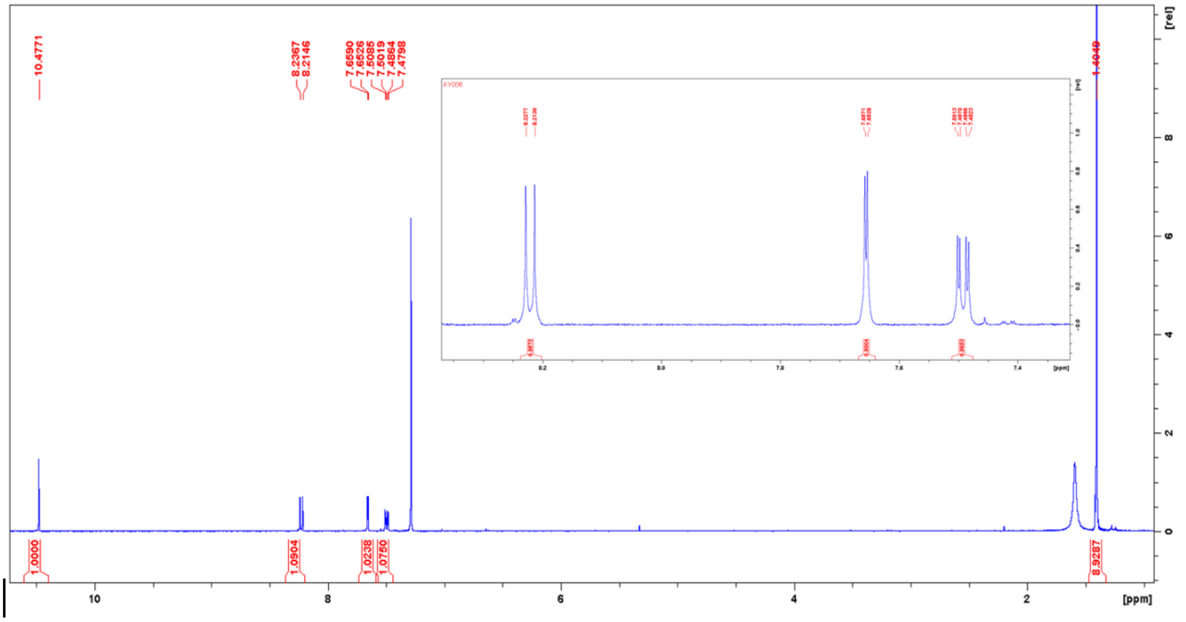

Figure S37. ^{1}H NMR spectrum of **10A** with expansion of 8.4 -7.4 ppm (400 MHz, *d*-chloroform, 25 °C).

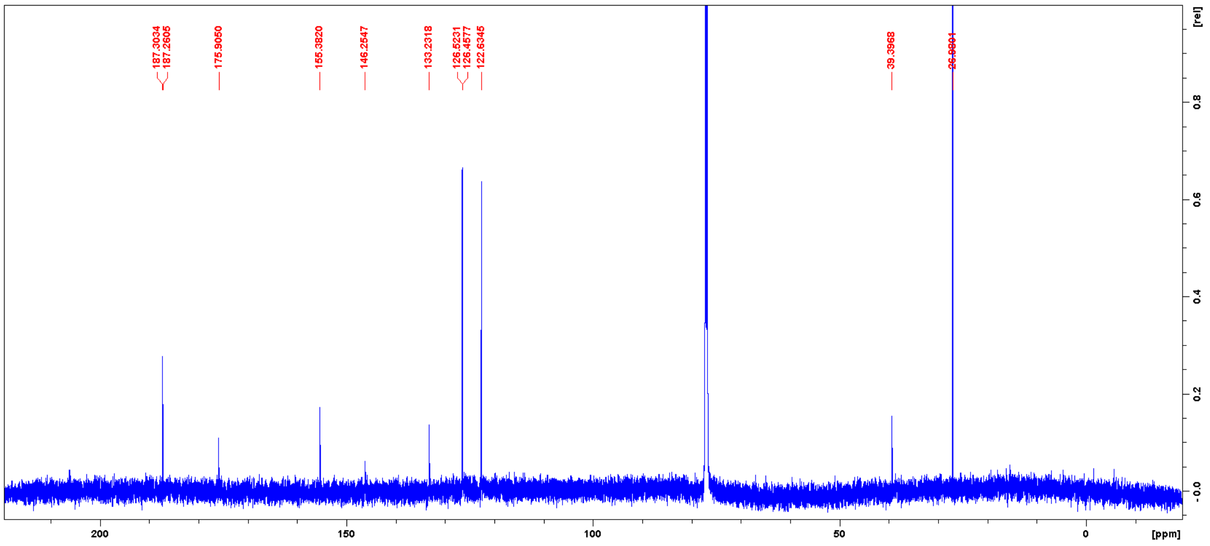

Figure S38. ^{13}C NMR spectrum of **10A** (101 MHz, *d*-chloroform, 25 °C).

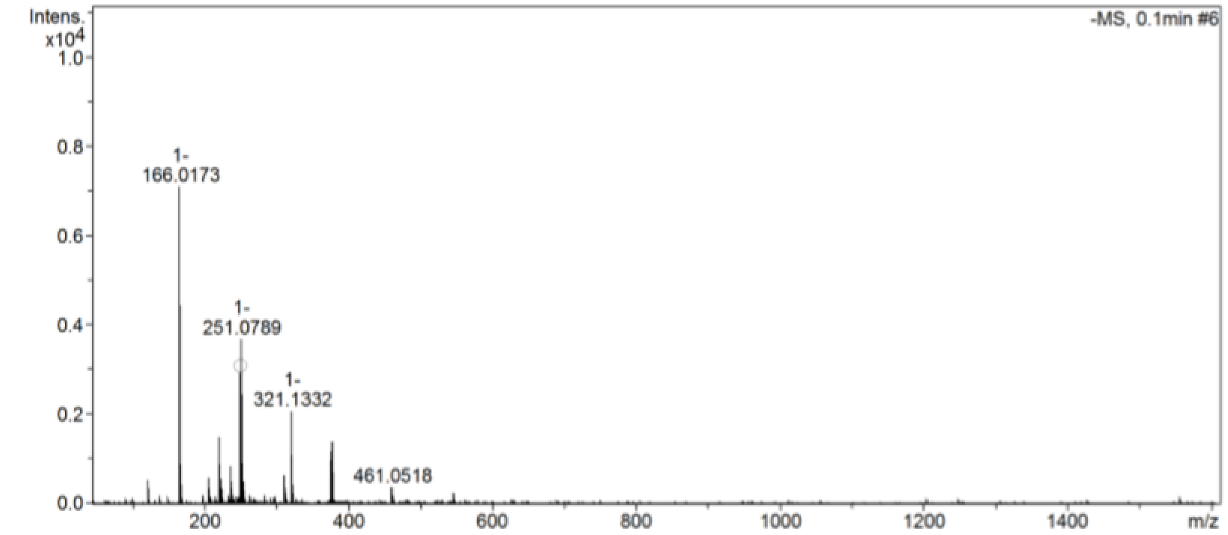

Meas. m/z	#	Ion Formula	m/z	err [mDa]	err [ppm]	rdb	N-Rule	e⁻ Conf	mSigma
250.071779	1	C12H12NO5	250.072096	-0.3	-1.3	7.5	ok	even	528.0

Figure S39. HRMS (APCI) of **10A**.

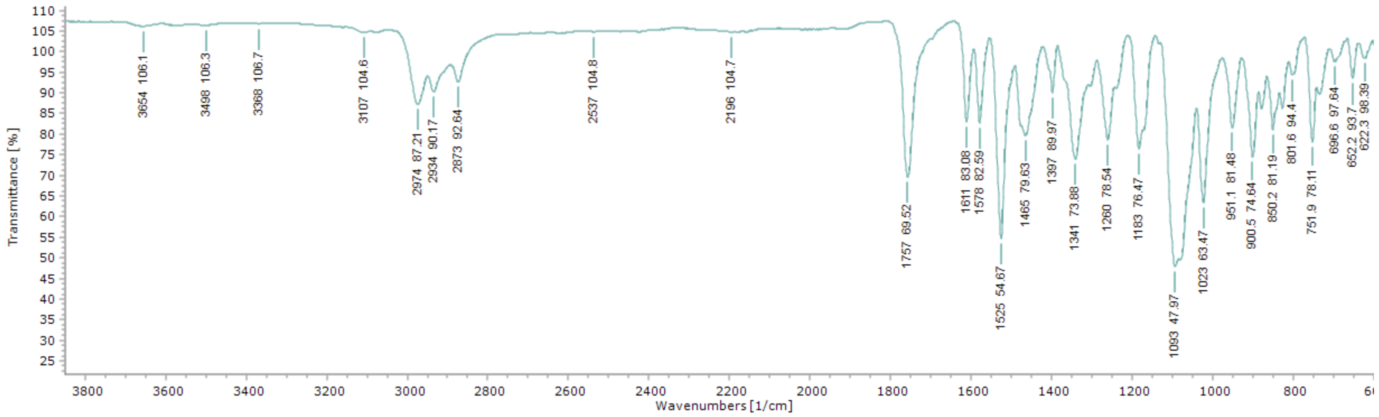

Figure S40. FTIR spectrum of **10A**.

Synthesis and characterization of **10**

2,3,7,8,12,13,17,18-Octaethylporphyrin-5,10,15,20-tetrakis(5-pivaloyloxy-2-nitrophenyl) [10]

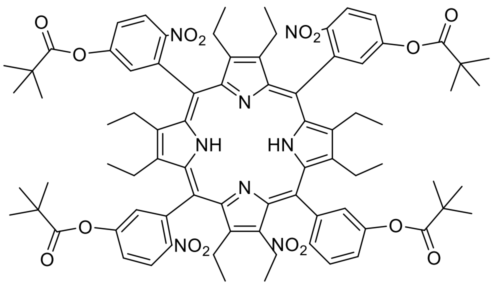

Synthesized *via* the General Procedure from 5-pivaloyloxy-2-nitrobenzaldehyde **10A** (0.934 g; 3.72 mmol; 1.0 eq.), 3,4-diethylpyrrole **9** (0.504 g; 4.09 mmol; 1.1 eq.), $BF_3 \times Et_2O$ (0.075 mL, 0.66 mmol) and DDQ (1.2 g, 5.29 mmol). The crude reaction mixture was quenched with 10% triethylamine. After aqueous work up, the concentrated organic extract was purified by silica gel column (CH_2Cl_2/EtOAc 10:1 *v/v*) and eluted (CH_2Cl_2/TEA 10:1 *v/v*) as a dark brown/green fraction. The residue was transferred to a 250 mL RBF and dissolved in chloroform (100 mL). *N,N*-Diisopropylethylamine (1.39 mL, 7.97 mmol, 8.0 eq.) was added and the reaction was treated dropwise with pivaloyl chloride (0.5 mL, 3.98 mmol, 4 eq.) and stirred for 1 h. The crude mixture was quenched with a few drops of NaOH, followed by extraction with dichloromethane, and washed with $NaHCO_3$ (1 × 150 mL) and water (1 × 150 mL). The organic phase was dried with anhydrous magnesium sulfate, filtered and dried under reduced pressure. Purification by a silica gel column removed black impurities (CH_2Cl_2/acetone 10:1 *v/v*) and the desired intermediate was collected (CH_2Cl_2/TEA 10:1 *v/v*) as a brown coloured product **10** [0.558 g, 3.93 mmol, 20%] HRMS (MALDI) *m/z* calcd. for $C_{80}H_{91}N_8O_{16}$ $[M+H]^+$: 1419.6553, found 1419.6549.

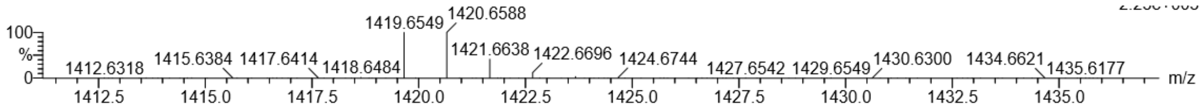

	Mass	Calc. Mass	mDa	PPM	DBE	i-FIT	i-FIT (Norm)	Formula
Minimum:					-1.5			
Maximum:			5.0	50.0	400.0			
	1419.6549	1419.6553	-0.4	-0.3	39.5	59.2	0.0	C80 H91 N8 O16

Figure S41. HRMS (MALDI) of **10**.

Synthesis and characterization of **12**

5,10,15,20-Tetrakis(5-pivaloyloxy-2-nitrophenyl)-2,3,7,8,12,13,17,18-octaethylporphyrinato]nickel(II) [12]

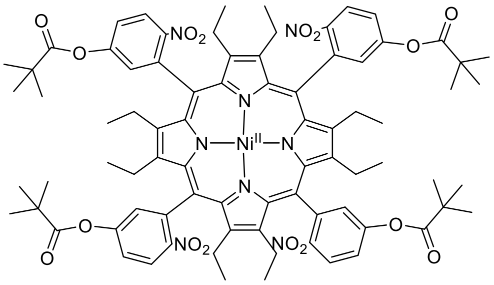

In a 250 mL RBF, the atropisomeric mixture of 10 (0.335 g, 0.236 mmol,1.0 eq.) was dissolved in toluene (100 mL) at room temperature. Nickel(II) acetylacetonate (0.303 g, 1.180 mmol, 5.0 eq.) was added and the reaction was heated to reflux at 111 °C. After 2 h, the mixture was cooled and solvent was evaporated *in vacuo*. The mixture was dissolved in a small volume of dichloromethane and transferred to a silica gel column (CH_2Cl_2). Three green fractions (CH_2Cl_2) were collected and dried under reduced pressure to yield three products as dichromatic (green/purple) crystalline solids:

α,β,α,β-**12** [87.2 mg, 0.059 mmol, 25%]; M.p.: >300°C; R_f = 0.82 (SiO_2, CH_2Cl_2); ^{1}H NMR (600 MHz, $CDCl_3$, 25 °C): δ= 8.35 (d, J_o= 8.7 Hz, 4H, *m*-phenyl-*H*), 7.70 (d, J_m= 2.6 Hz, 4H, *o*-phenyl-*H*), 7.57 (dd, J_o= 8.8 Hz, J_m= 2.2 Hz, 4H, *p*-phenyl-*H*), 2.40 (br, 16H, -C*H2*), 1.45 (s, 36H, *t*-Bu), 0.62 (br, 24H, -CH_2C*H3*) ppm; ^{13}C NMR (100 MHz, $CDCl_3$, 25 °C): δ= 176.45, 153.18, 148.90, 144.03, 136.41, 130.28, 125.60, 123.10, 111.40, 39.40, 27.09, 19.55 ppm; UV/Vis (chloroform): λ_{max} [nm] (log ε[L·mol^{-1} ·cm^{-1}]) = 442.6 (5.00), 572.2 (4.07), 611.3 (4.04); HRMS (MALDI) *m/z* calcd. for $C_{80}H_{88}N_8NiO_{16}$ [M]$^+$: 1474.5672, found 1474.5699; IR (ATR) $\tilde{\nu}$ = 2972.50, 2934.84, 2873.17, 1757.33 (s, C=O stretch.), 1610.59, 1578.45, 1523.98 (s, -NO asym. stretch.), 1466.13 (m, C*H2* bend), 1397.58, 1342.75 (s, -NO sym. stretch.), 1261.73, 1184.14, 1081.52 (s, C-O stretch.), 1023.05 (sh, C-O stretch.), 950.37, 900.54, 853.95, 828.03, 801.43, 752.00, 699.37, 651.67, 617.94 cm^{-1}.

α2β2-**12** [50.7 mg, 0.034 mmol, 15%]; M.p.: 279-280°C (decomposition); R_f = 0.56 (SiO_2, CH_2Cl_2); ^{1}H NMR (600 MHz, $CDCl_3$, 25 °C): δ= 8.35 (d, J_o= 8.7 Hz, 4H, *m*-phenyl-*H*), 7.90 (br, 4H, *o*-phenyl-*H*), 7.57 (dd, J_o=8.9 Hz, J_m= 2.0 Hz, 4H, *p*-phenyl-*H*), 2.40 (br, 16H, -C*H2*), 1.46 (s, 36H, *t*-Bu), 0.63 (br, 24H, -CH_2C*H3*) ppm; ^{13}C NMR (100 MHz, $CDCl_3$, 25 °C): δ= 176.37, 152.89, 149.31, 144.80, 144.18, 136.32, 129.92, 125.79, 122.95, 39.40, 27.11, 19.52; UV/Vis (chloroform): λ_{max} [nm] (log ε[L·mol^{-1} ·cm^{-1}]) = 442.1 (4.97), 573.1 (4.05), 609.4 (4.01); HRMS (MALDI) *m/z* calcd. for $C_{80}H_{88}N_8NiO_{16}$ [M]$^+$: 1474.5672, found 1474.5707; IR (ATR) $\tilde{\nu}$ = 2973.48, 2934.36, 2873.37, 1758.47 (s, C=O stretch.), 1611.04, 1578.83, 1525.46 (s, -NO asym. stretch), 1465.10 (m, C*H2*- bend.), 1397.75, 1341.15 (s, -NO sym. stretch.), 1260.08, 1182.72, 1092.89 (s, C-O stretch.), 1022.92 (sh, C-O stretch.), 951.10, 900.74, 851.23, 826.04, 800.37, 752.30, 697.90, 652.48, 620.27, 578.09 cm^{-1}.

α3β-**12:** [76.6 mg, 0.052 mmol, 22%]; M.p.: 272-276°C (decomposition); R_f = 0.44 (SiO_2, CH_2Cl_2); ^{1}H NMR (600 MHz, $CDCl_3$, 25 °C): δ= 8.44 – 8.32 (m, 4H, *m*-phenyl-*H*), 8.10 (s, 1H, *o*-phenyl-*H*), 8.04 – 7.68 (br, 2H, *o*-phenyl-*H*), 7.61 (s, 1H, *o*-phenyl-*H*), 7.60 – 7.53 (m, 4H, *p*-phenyl-*H*), 2.41 (br, 16H, -C*H2*), 1.45 (s, 36H, *t*-Bu), 0.63 (br, 24H, -CH_2C*H3*) ppm; ^{13}C NMR (100 MHz, $CDCl_3$, 25 °C): δ= 176.36, 153.32, 152.86, 152.62, 149.59, 148.48, 143.92, 136.48, 136.30, 136.12, 130.70, 129.75, 129.57, 126.08, 125.90, 125.52, 123.12, 122.94, 122.80, 111.76, 111.27, 39.39, 27.10, 19.53; UV/Vis (chloroform): λ_{max} [nm] (log ε[L·mol^{-1}·cm^{-1}]) = 442.7 (5.01), 571.8 (4.10), 610.8 (4.06); HRMS (MALDI) *m/z* calcd. for $C_{80}H_{88}N_8NiO_{16}$ [M]$^+$: 1474.5672, found 1474.5724; IR (ATR) $\tilde{\nu}$ = 2973.74, 2934.01, 2873.13, 1757.65 (s, C=O stretch.), 1610.96, 1578.41, 1525.07 (s, -NO asym. stretch), 1465.21 (m, C*H2* bend.), 1397.54, 1340.27 (s, -NO sym. stretch.), 1260.09, 1182.28, 1092.81 (s, C-O stretch.), 1022.79 (sh, C-O stretch.), 951.19, 900.55, 850.52, 826.47, 800.62, 752.16, 697.14, 651.97, 620.27, 575.07 cm^{-1}.

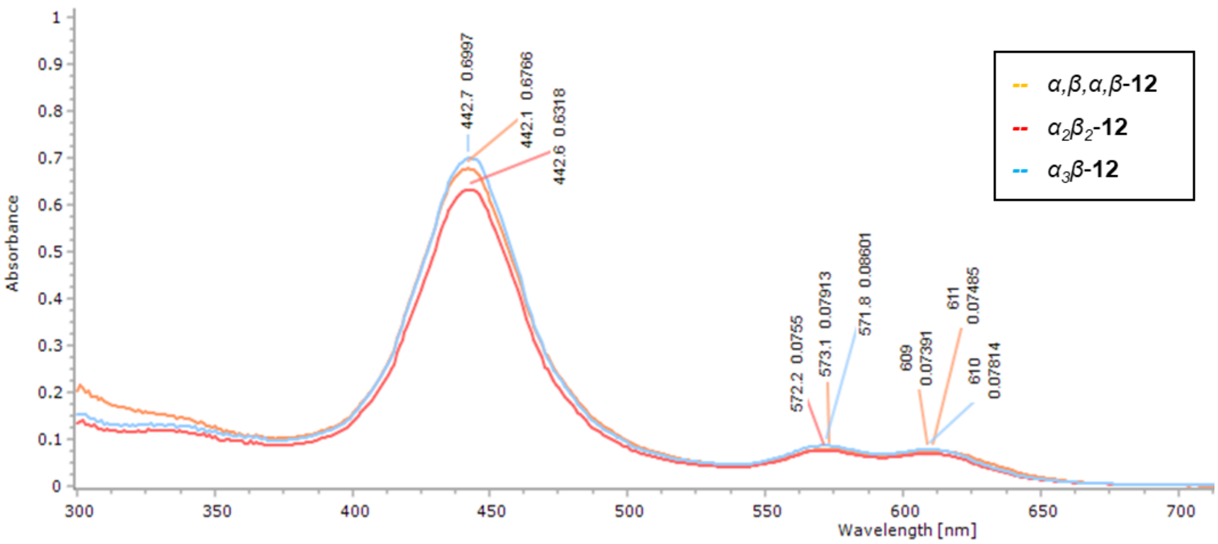

Figure S42. UV-vis spectrum of *α,β,α,β*-**12**, *α2β2*-**12** and *α3β*-**12** in chloroform.

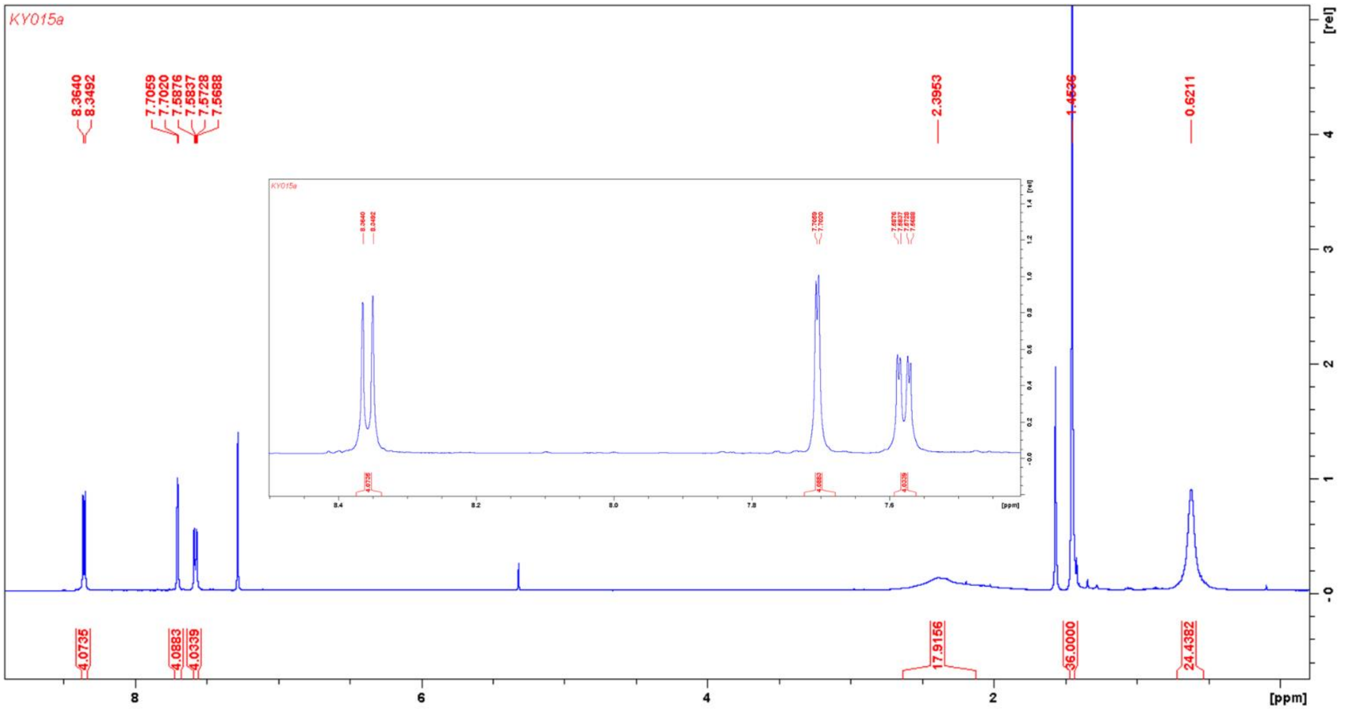

Figure S43. ^{1}H NMR spectrum of *α,β,α,β*-**12** with expansion of 8.5 – 7.4 ppm (600 MHz, *d*-chloroform, 25 °C).

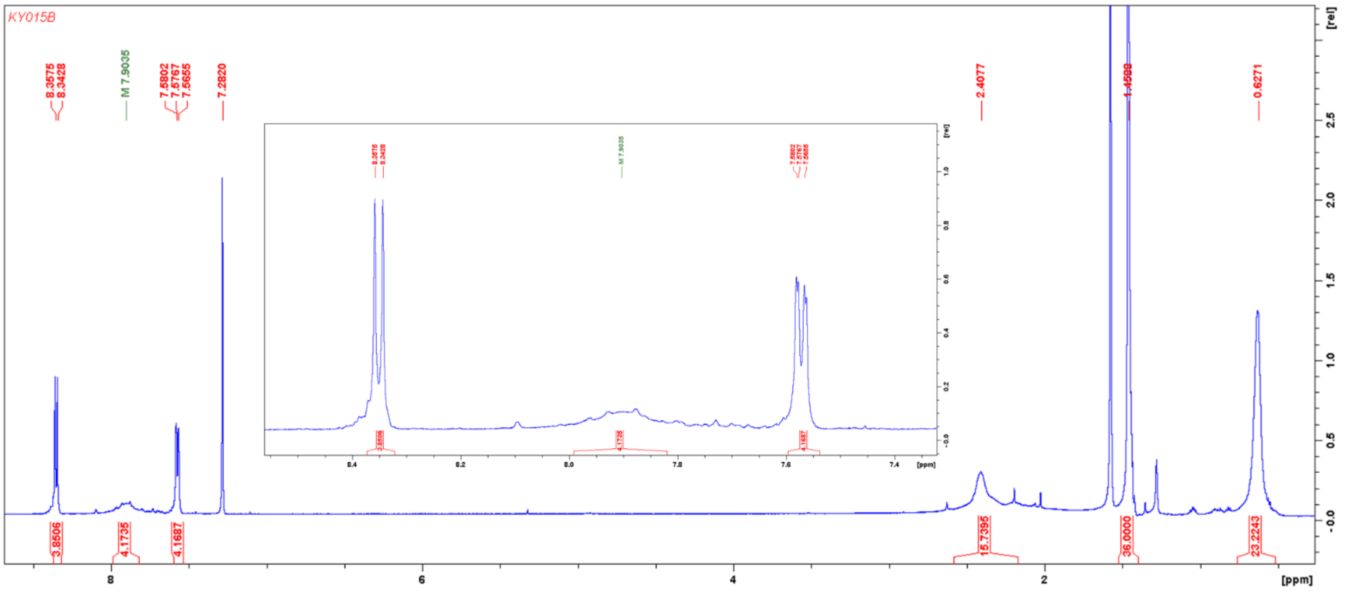

Figure S44. ^{1}H NMR spectrum of $α_2β_2$-**12** with expansion of 8.5 – 7.4 ppm (600 MHz, *d*-chloroform, 25 °C).

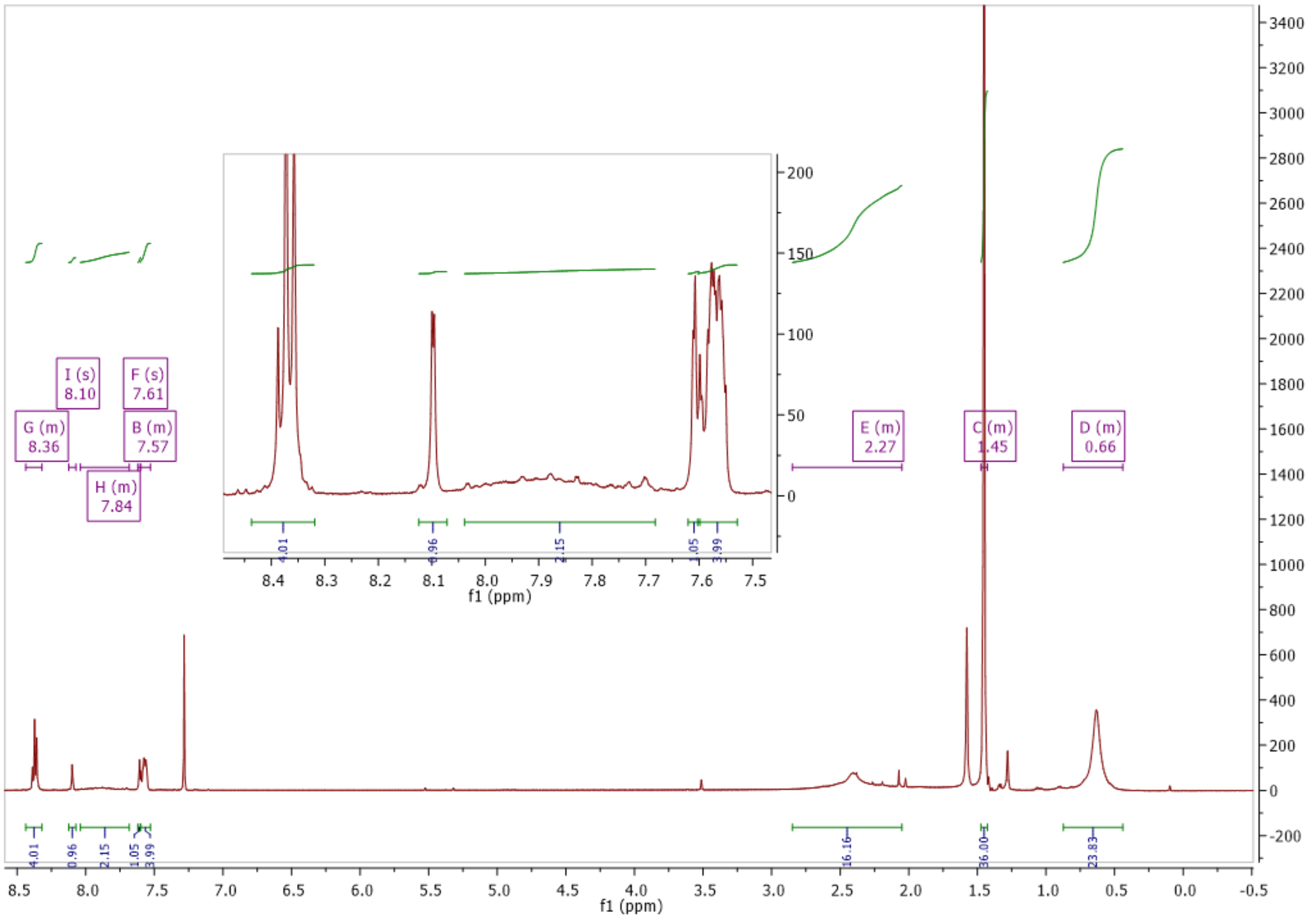

Figure S45. ^{1}H NMR spectrum of *α₃β*-**12** with expansion of 8.5 – 7.5 ppm (600 MHz, *d*-chloroform, 25 °C).

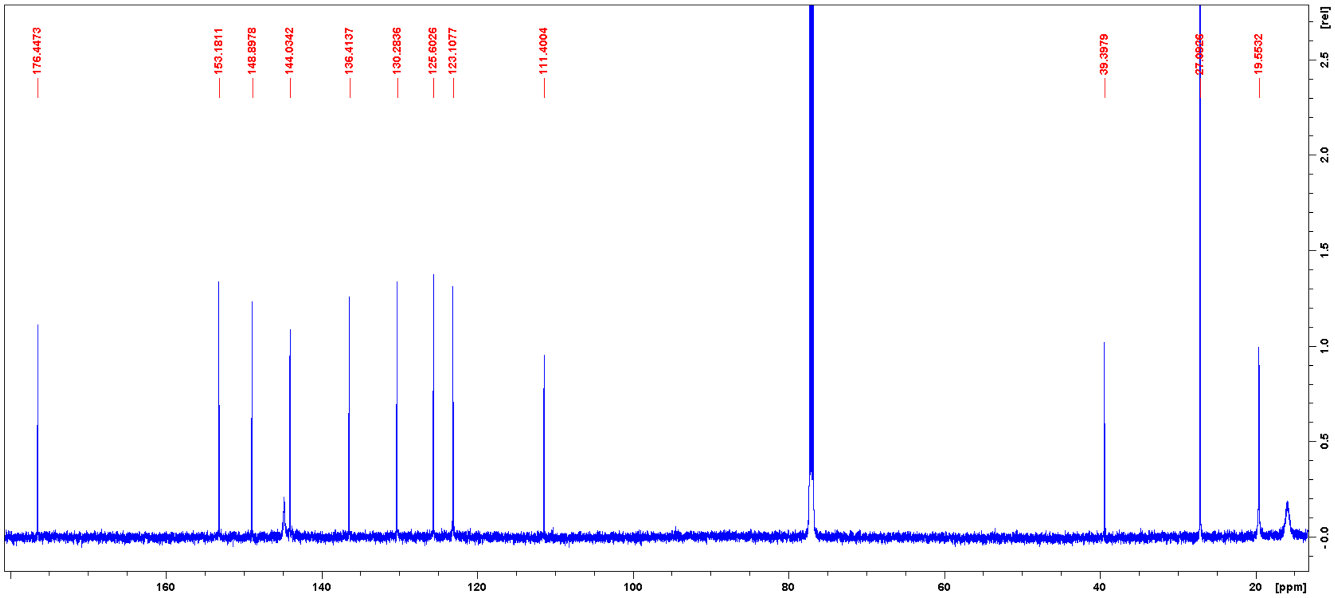

Figure S46. ^{13}C NMR spectrum of *α,β,α,β*-**12** (101 MHz, *d*-chloroform, 25 °C).

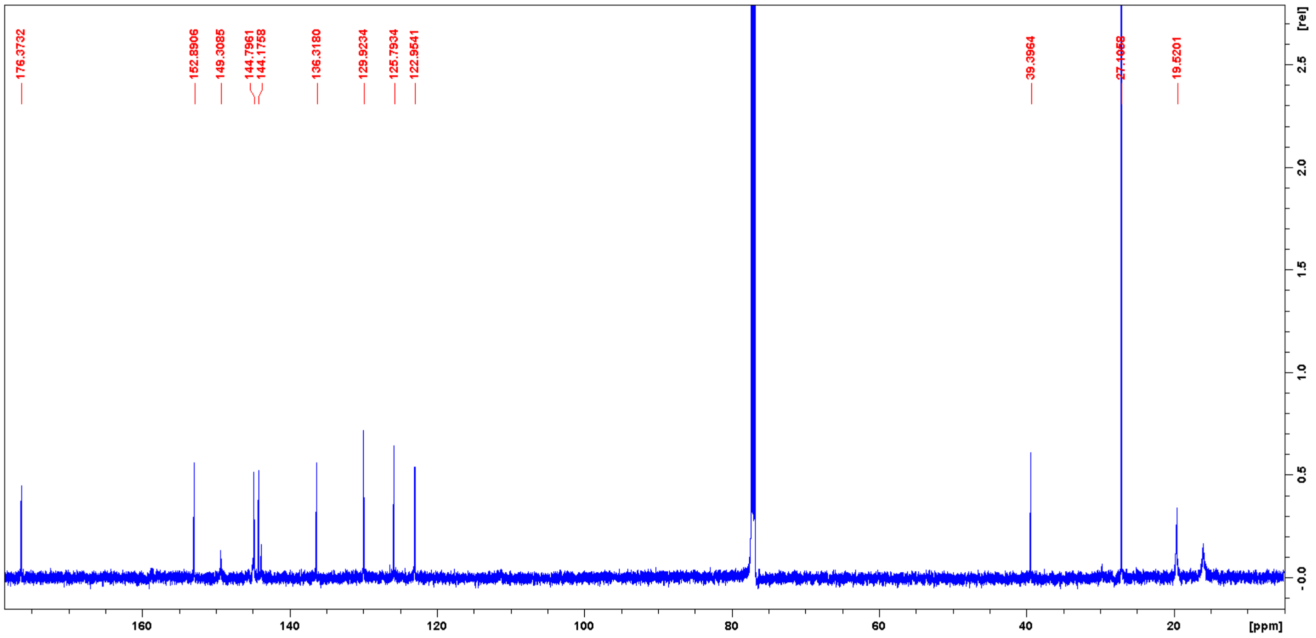

Figure S47. ^{13}C NMR spectrum of *α₂β₂*-**12** (101 MHz, *d*-chloroform, 25 °C).

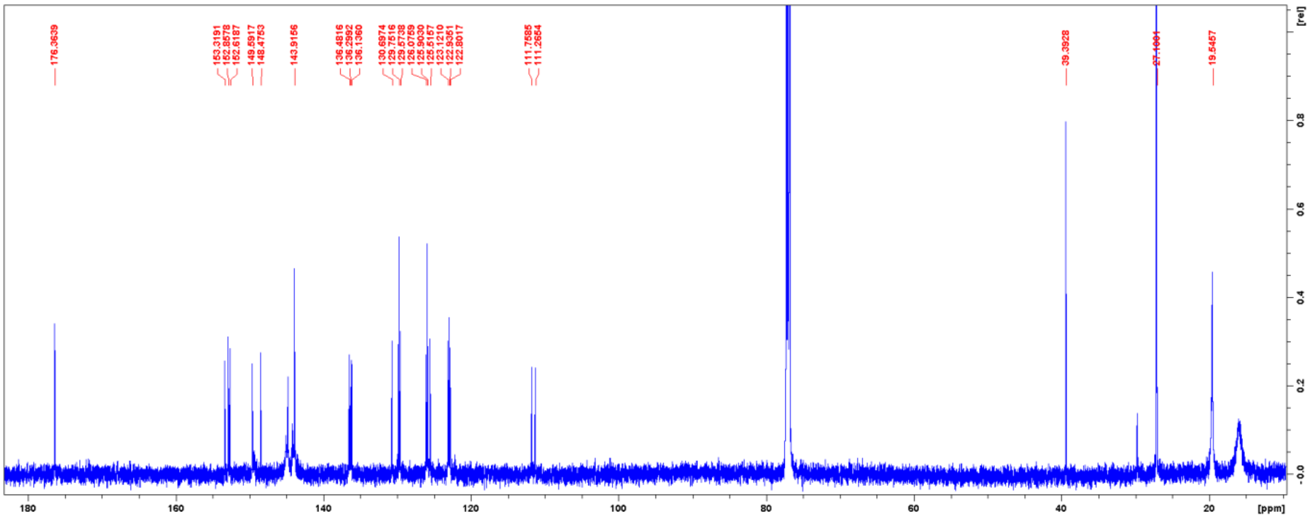

Figure S48. ^{13}C NMR spectrum of *α₃β*-**12** (101 MHz, *d*-chloroform, 25 °C).

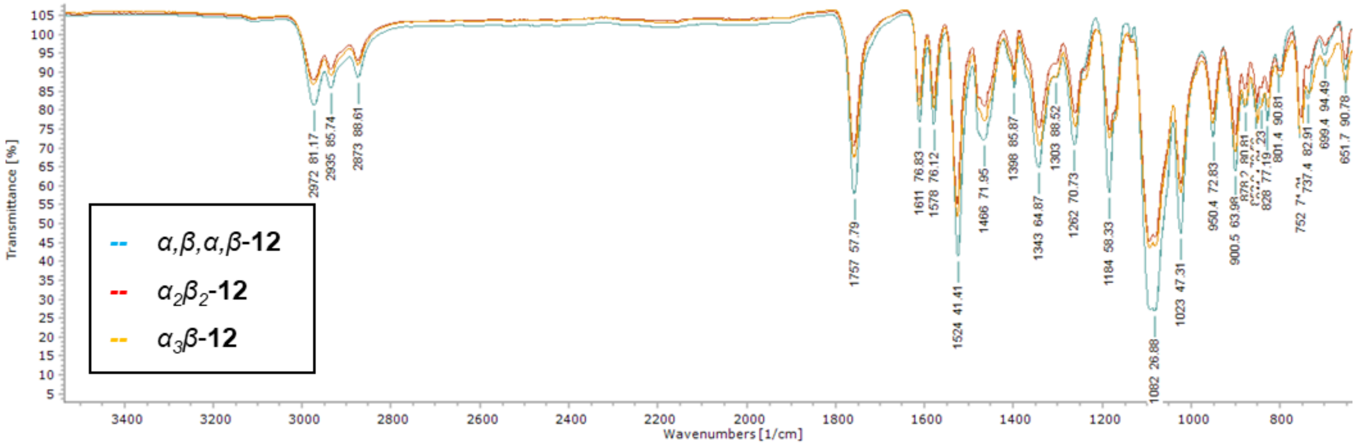

Figure S49. FTIR spectrum of *α,β,α,β*-**12**, *α₂β₂*-**12** and *α₃β*-**12**.

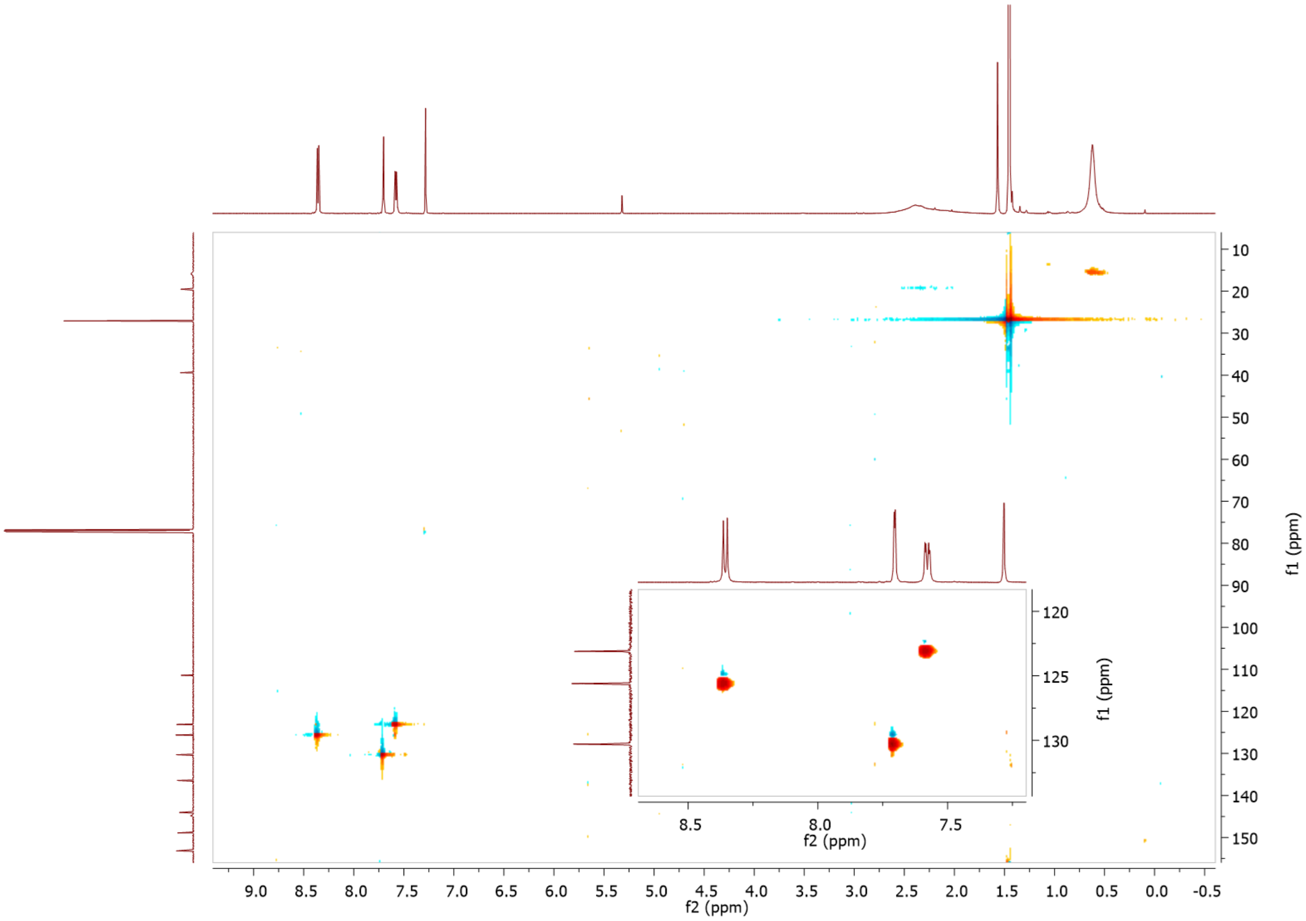

Figure S50. ^{13}C–^{1}H HSQC spectrum with expansion in aromatic region of *α,β,α,β*-**12** (*d*-chloroform, 25 °C).

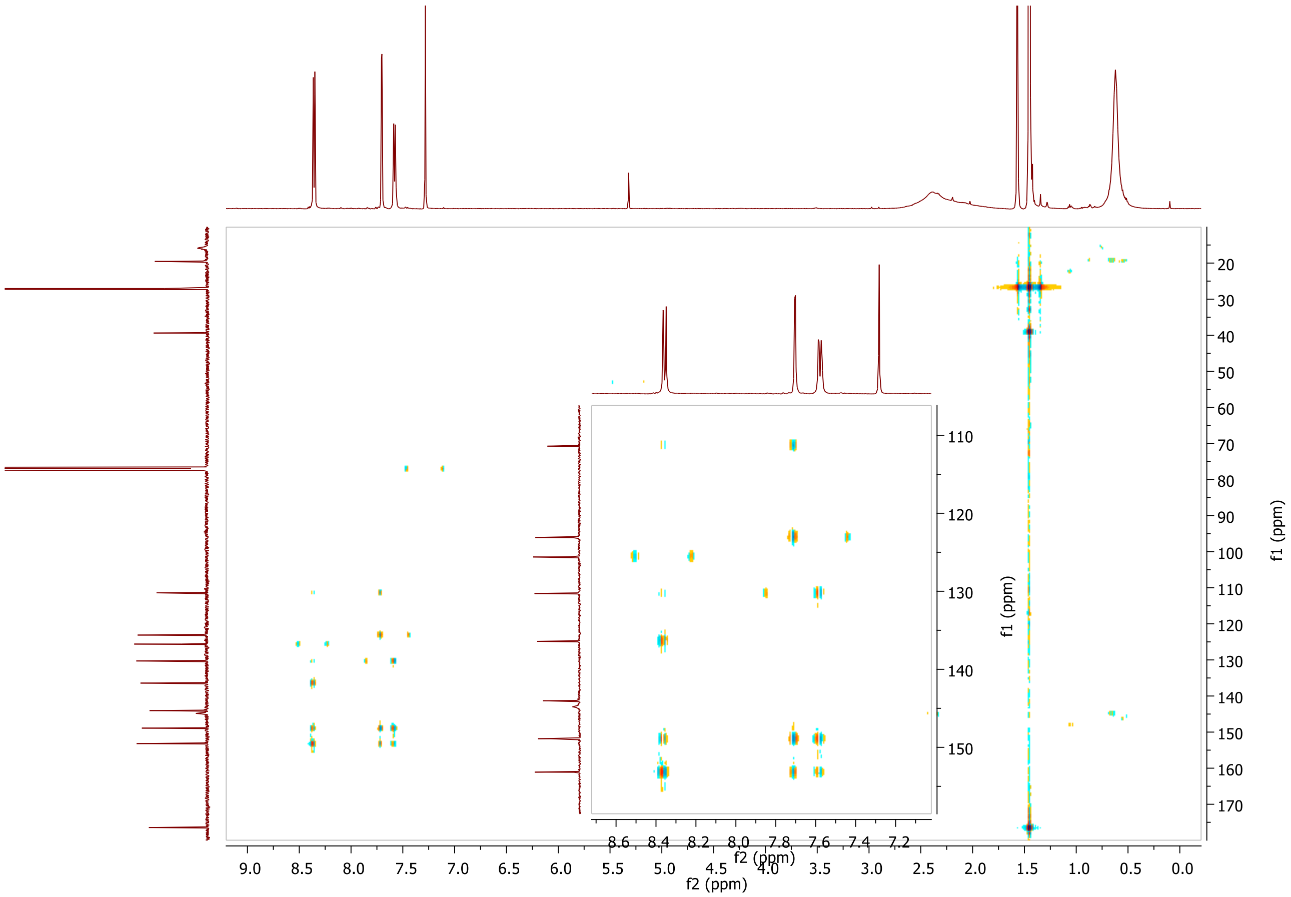

Figure S51. ^{13}C–^{1}H HMBC spectrum with expansion in aromatic region of *α,β,α,β*-**12** (*d*-chloroform, 25 °C).

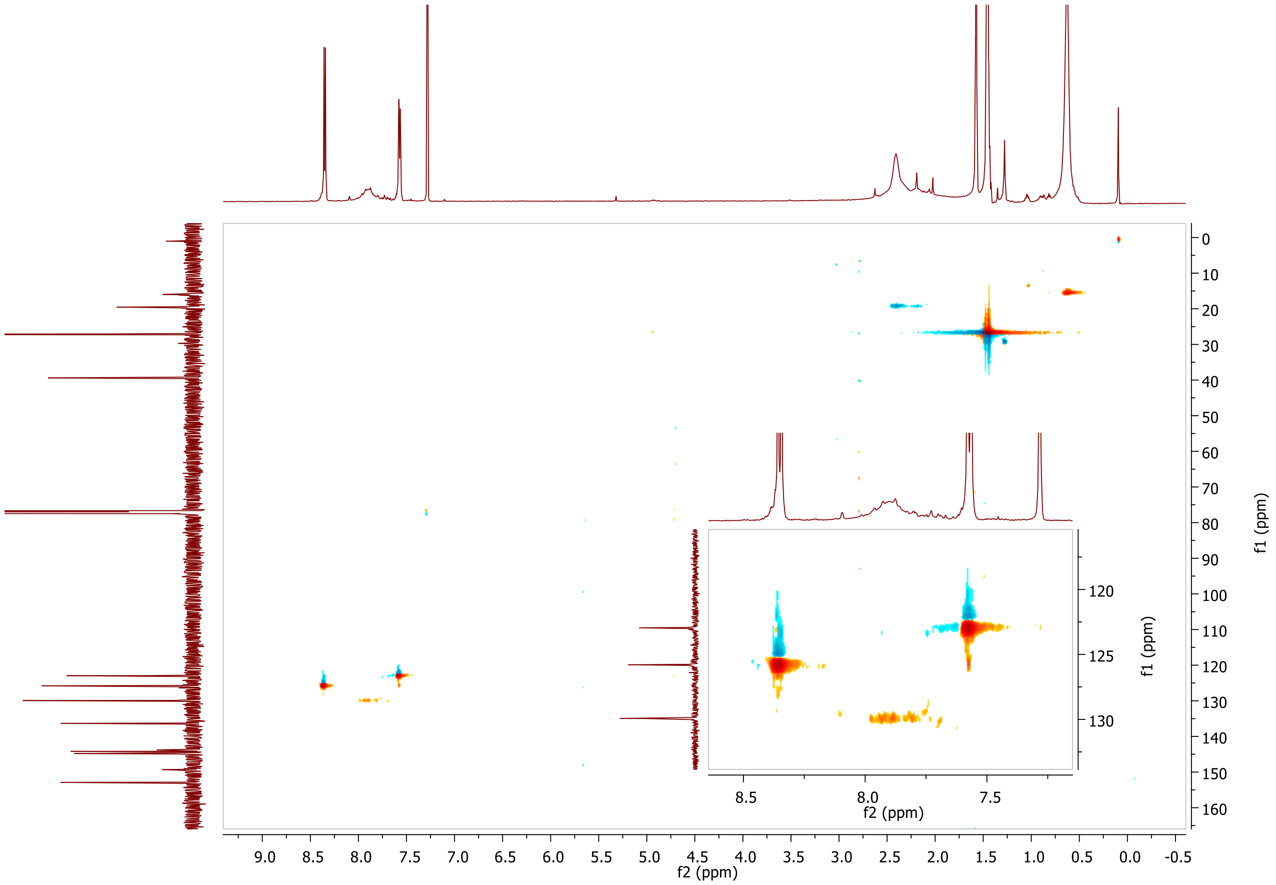

Figure S52. ^{13}C–^{1}H HSQC spectrum with expansion in aromatic region of *α*$_2$*β*$_2$-**12** (*d*-chloroform, 25 °C).

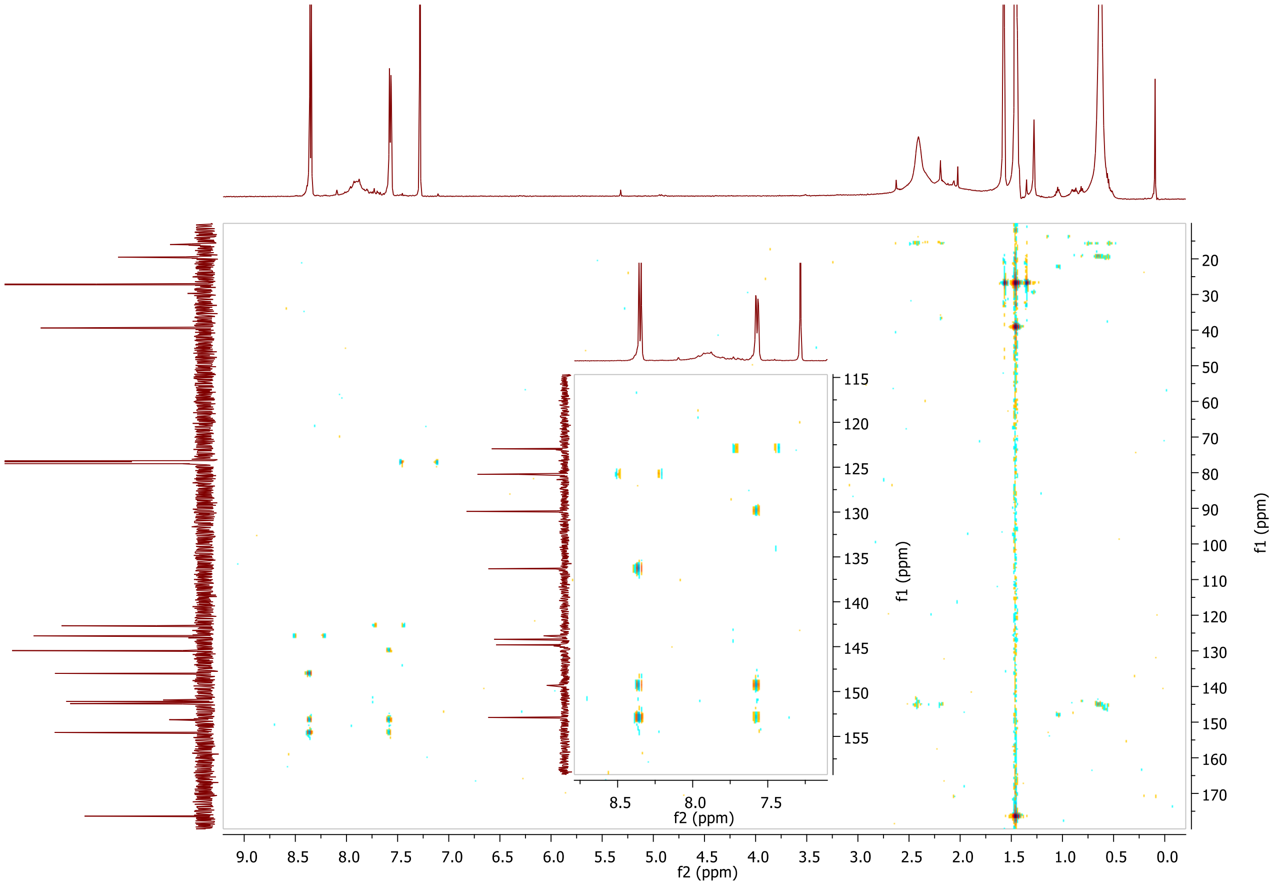

Figure S53. ^{13}C–^{1}H HMBC spectrum with expansion in aromatic region of *α*$_2$*β*$_2$-**12** (*d*-chloroform, 25 °C).

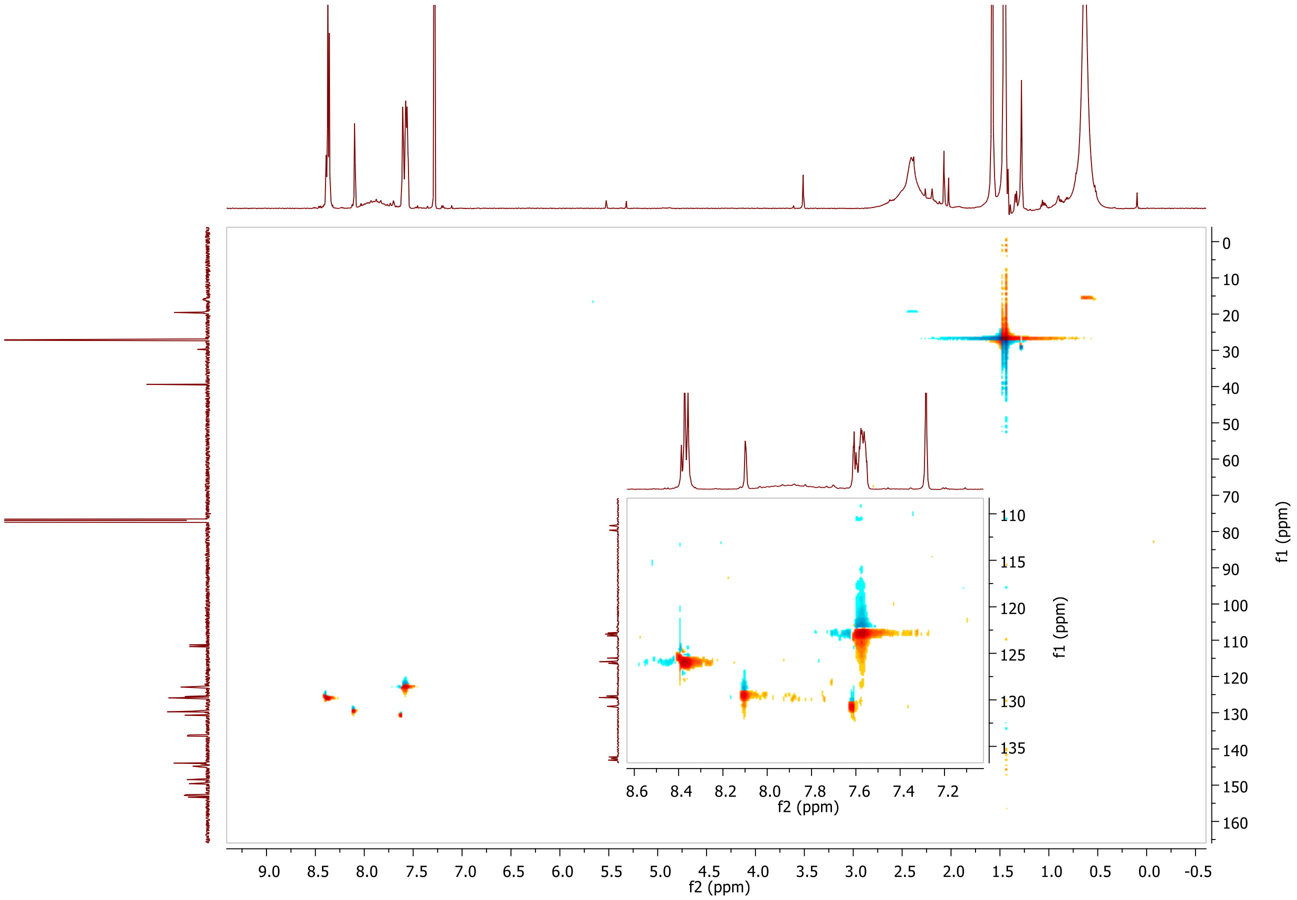

Figure S54. ^{13}C–^{1}H HSQC spectrum with expansion in aromatic region of $\alpha_3\beta$-**12** (*d*-chloroform, 25 °C).

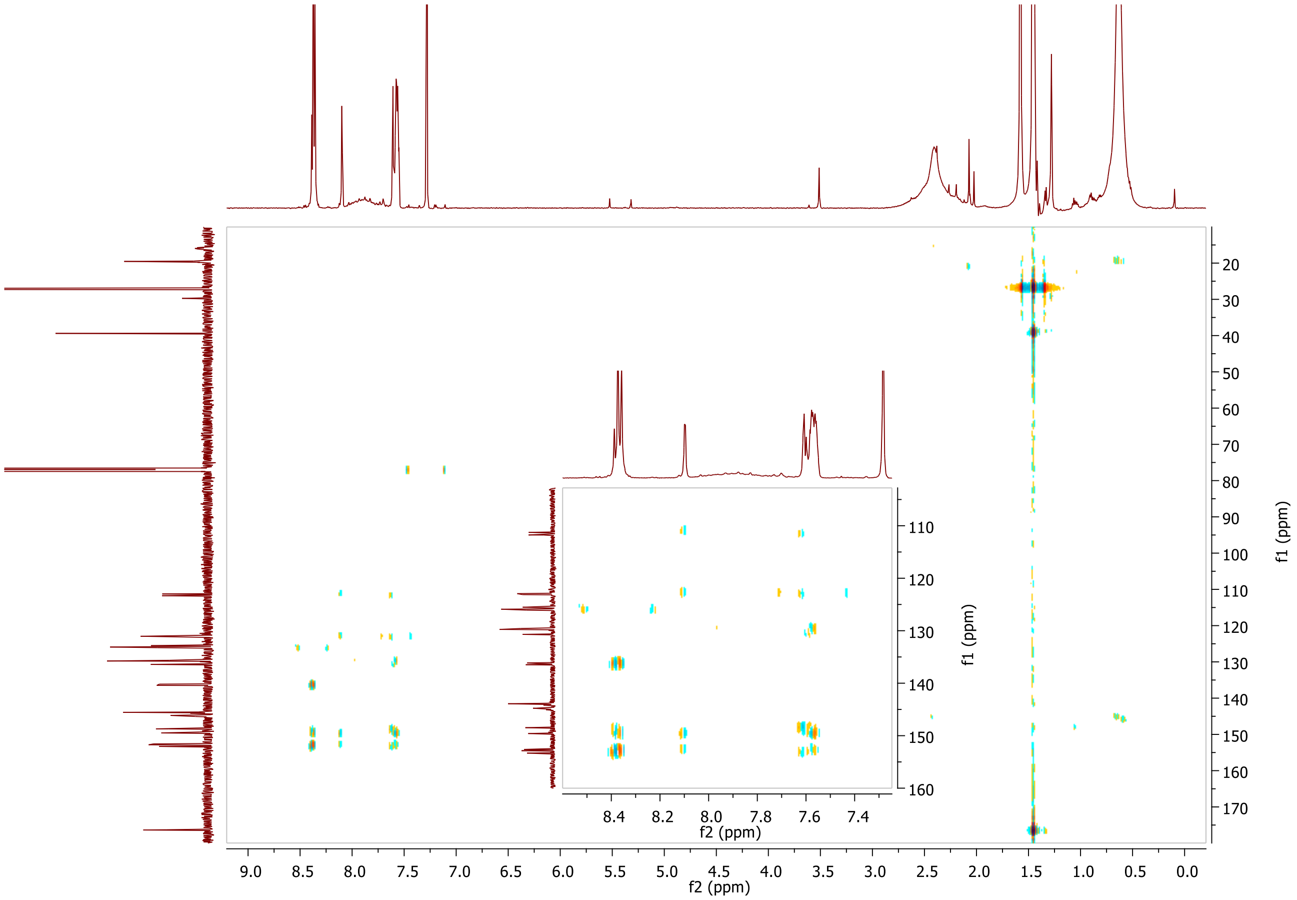

Figure S55. ^{13}C–^{1}H HMBC spectrum with expansion in aromatic region of $\alpha_3\beta$-**12** (*d*-chloroform, 25 °C).

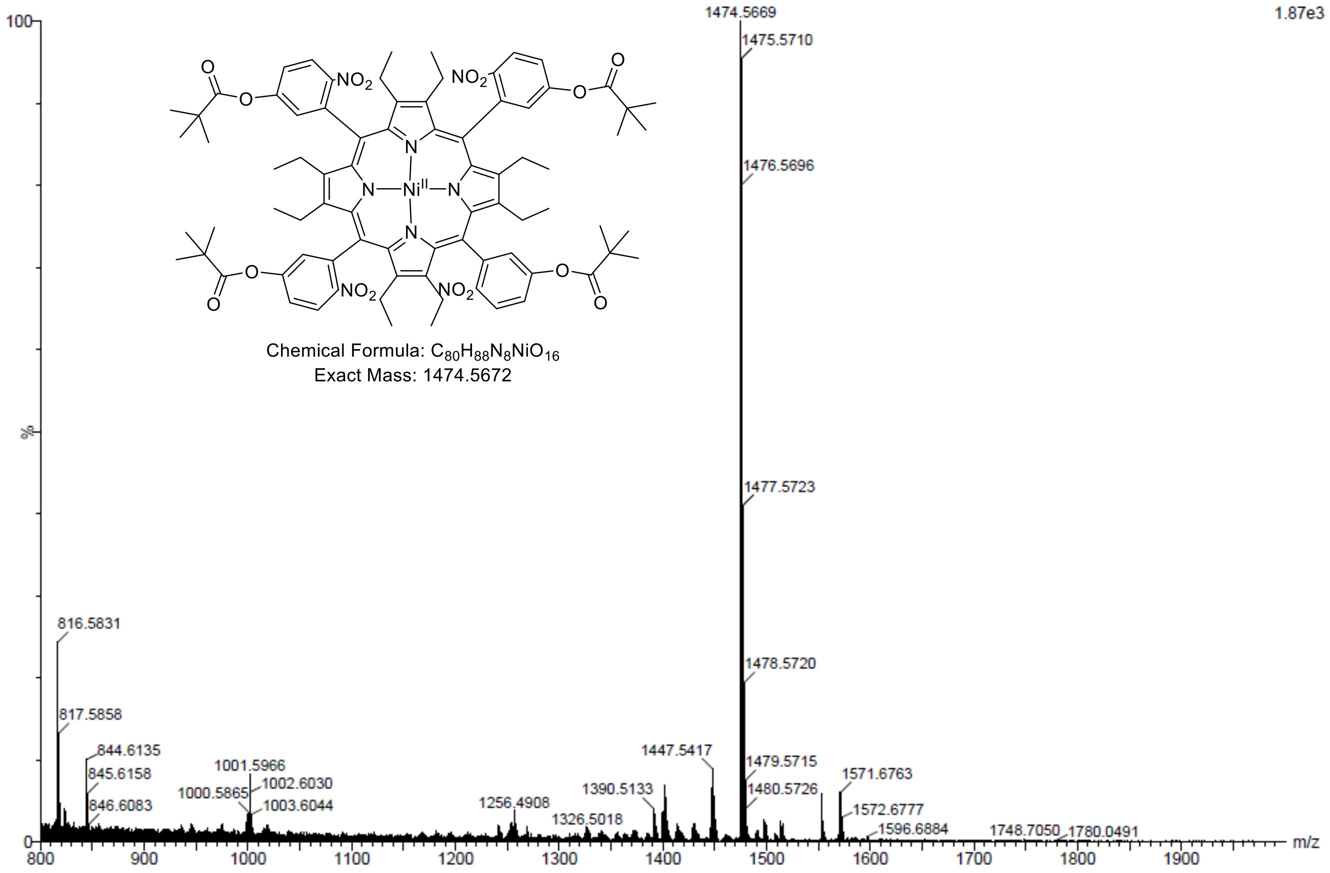

Figure S56. HRMS (MALDI) of *α,β,α,β*-**12**.

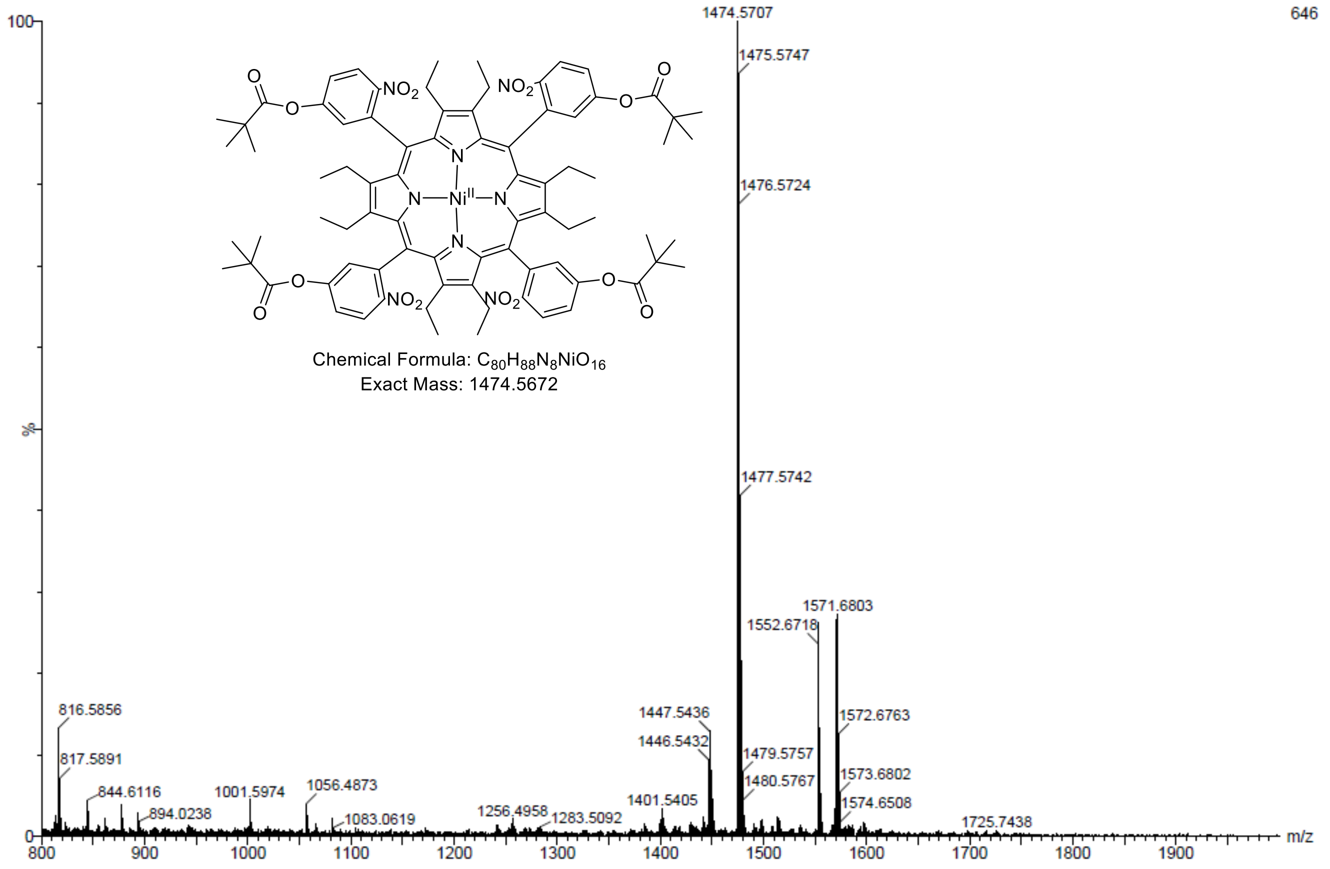

Figure S57. HRMS (MALDI) of $\alpha_2\beta_2$-**12**.

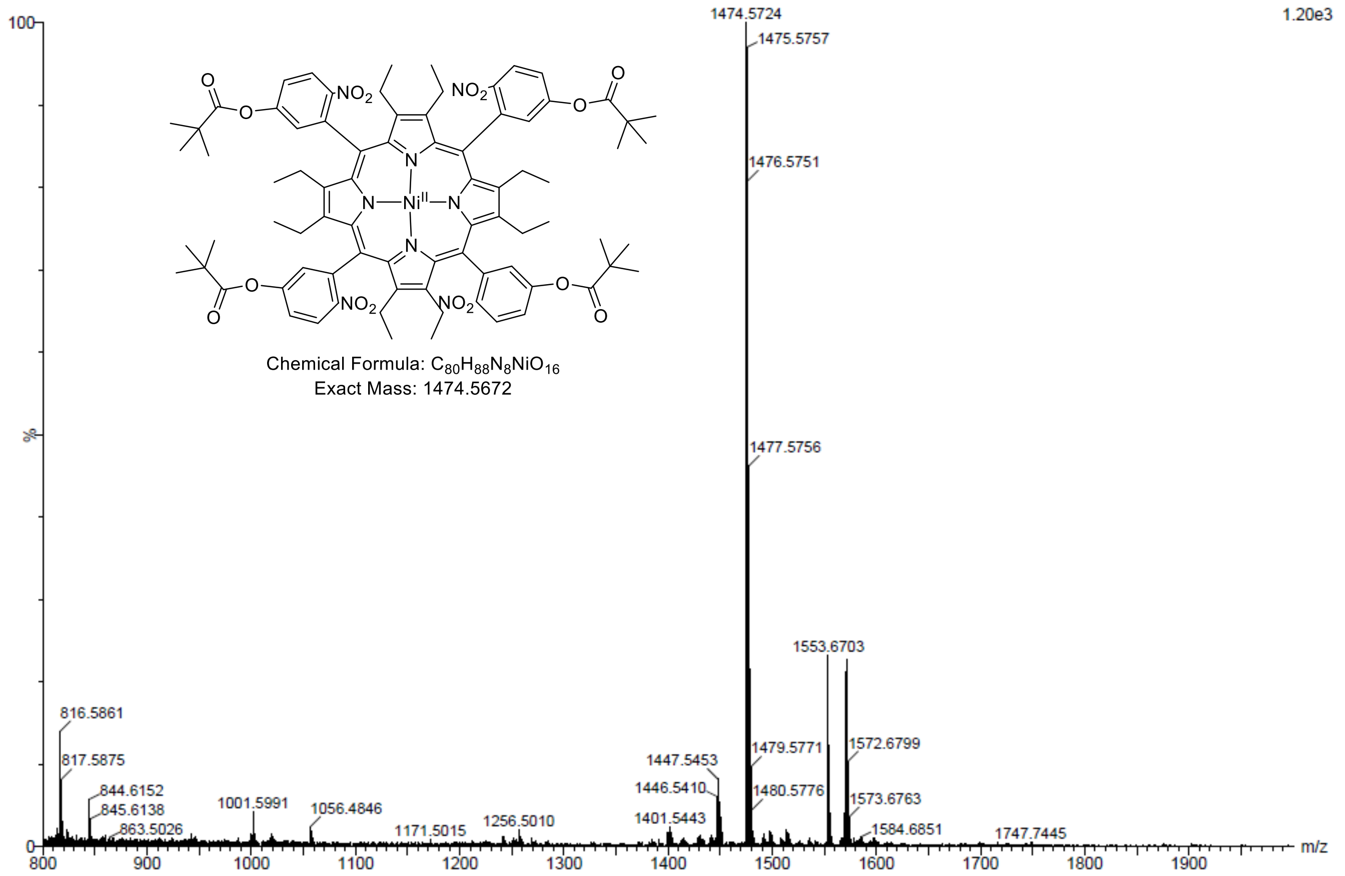

Figure S58. HRMS (MALDI) of *α₃β*-**12**.

Results and Discussion

Structural Determination of Isolated Structures

Crystals were grown following the protocol developed by Hope, liquid-liquid diffusion in $CHCl_3$ and methanol or oversaturated solutions in DMSO.[7] Using Olex2, the structure was solved with the XT structure solution program, using the intrinsic phasing solution method and refined against $|F^2|$ with XL using least squares minimization.[8] The C and N bound H atoms were placed in their expected calculated positions and refined as riding model: N–H = 0.88 Å, C–H = 0.95–0.98 Å, with U_{iso} (H) = 1.5U_{eq} (C) for methyl H atoms and 1.2U_{eq} (C, N) for all other atoms other H atoms. Details of data refinements can be found in Table S1. All images were prepared by using Olex2.[8a]

In the structure of **9A** one t-Bu acetate group modelled as disordered over two positions with almost equal occupancy (53/47%) using restraints (SADI and ISOR) and constraints (EADP/EXYZ for C17C17a and EADP C19/C19a).

In the structure of **9** a pivaloyl group at O114 was modelled over two positions using SIMU restraint in a 72:28% occupancy. Two of the trifluoroacetate molecules was modelled over two positions using rigid models in a 50:50% occupancies.

In the structure of **8** the phenyl moiety at C10_2 was modelled over two positions using restraints (SADI, SIMU, ISOR, AFIX 66) in a 54:46 % occupancy. The ethyl groups at C18_2, C7_2, C12_1 were modelled over two positions using restraints (SADI, SIMU) in 58:42 %, 54:46 %, 75:25 % occupancies respectively. DMSO molecules were modelled using rigid models, moreover, units at S26S, S7S and S1S were modelled over two positions in 25:75, 40:60, 40:60 % occupancies. In the structure there were solvent accessible voids that contained large amounts of solvent molecules, however, due to high disorder these could not be modelled reliably and were omitted using OLEX2 maps.

In the structure of $\alpha_3\beta$-**12** one ethyl group at C18 was disordered and modelled in two locations (36:54% occupied) with restraints (SADI, SIMU). Three pivaloate groups at C10, C15, C20 disordered and modelled in two locations (57:33%; 26:74%; 85:15% correspondingly) with restraints (SADI, SIMU). The solvent molecules in the lattice void were modelled with rigid groups and consist of DMSO

In the structure of $\alpha_2\beta_2$-***12*** one ethyl group at C2 was disordered and modelled in two locations (51:49% occupied) with restraints (SADI, SIMU). One pyrrole ring carbon and ethyl group at C13 was disordered and modelled in two locations (64:36% occupied) with restraints (SADI, SIMU). One pivaloate group at C15 disordered and modelled in two locations (52:48%) with restraints (SADI, SIMU). The solvents in the lattice void were modelled with rigid groups and consist of DMSO and H2O.

The structure *α,β,α,β*-***12*** was refined as a 2-component twin. In the first unit, two pivaloate groups at C15, C20 disordered and modelled in two locations (57:43%; 60:40% correspondingly) with restraints (SADI, SIMU). Two nitro groups at C5, C15 disordered and modelled in two locations (61:49%; 47:53% correspondingly) with restraints (SADI, SIMU). In the second unit, two pivaloate groups at C10B, C15B disordered and modelled in two locations (52:48%; 56:44% correspondingly) with restraints (SADI, SIMU). The solvent molecules in the lattice void were modelled with rigid groups and consist of chloroform and dichloromethane.

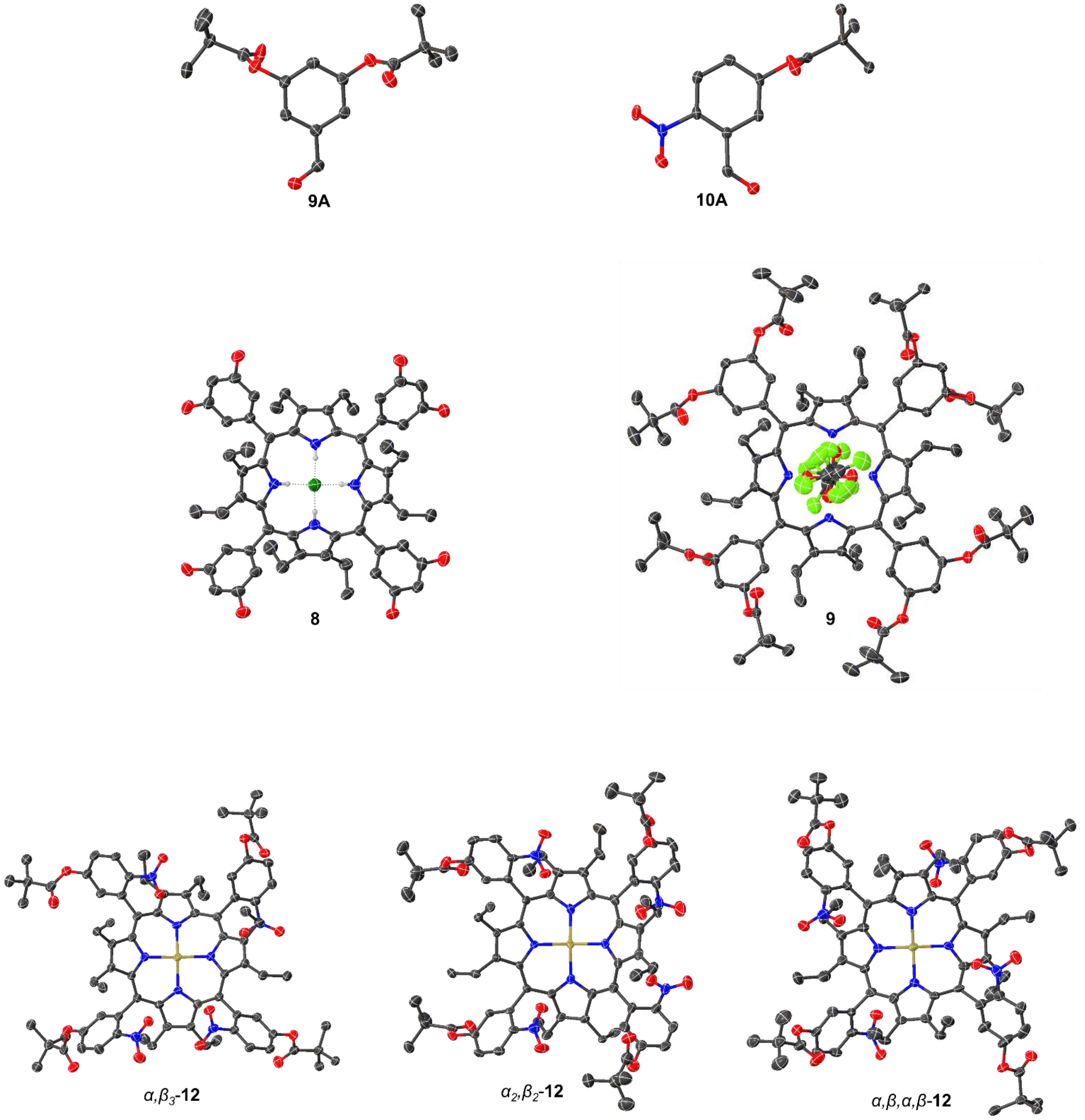

Figure S59. Molecular structures of isolated and analyzed compounds by X-ray crystallography. Hydrogen atoms and solvent molecules omitted for clarity; thermal ellipsoids give 50% probability.

Table S1: Details of XRD data refinement of *α,β,α,β-**12**, α₂β₂-**12***, *α₃β*-**12**, **8**, **9**, **10A**, **9A**.

Compound	*α,β,α,β-**12***	*$\alpha_2\beta_2$-**12***	*$\alpha_3\beta$*-**12**	**8**	**9**	**10A**	**9A**
Internal code	KN016b	KN016	KN015	KN013	KN012	KN011	TCD1366
CCDC #	2058266	2058269	2058267	2058270	2058268	2058264	2058265
Empirical formula	$C_{162}H_{179}Cl_5N_{16}Ni_2O_{32}$	$C_{86.56}H_{108.21}N_8NiO_{19.79}S_{3.29}$	$C_{85}H_{103}N_8NiO_{18.5}S_{2.5}$	$C_{134}H_{172}Cl_4N_8O_{24}S_7$	$C_{56}H_{67}D_6F_3N_3O_{11}S$	$C_{12}H_{13}NO_5$	$C_{17}H_{22}O_5$
Formula weight	3156.87	1741.38	1671.61	2645.01	1059.27	251.23	306.34
Temperature/K	100(2)	100(2)	100(2)	100(2)	100(2)	100(2)	100(2)
Crystal system	Triclinic	Triclinic	Triclinic	Triclinic	Monoclinic	Triclinic	Monoclinic
Space group	$P\bar{1}$	$P\bar{1}$	$P\bar{1}$	$P\bar{1}$	C2/c	$P\bar{1}$	C2/c
a/Å	14.6355(6)	15.936(3)	11.5285(3)	19.4431(8)	35.1721(10)	5.960(9)	18.2037(7)
b/Å	23.0234(10)	18.284(4)	12.6909(3)	19.5835(7)	15.7125(5)	8.693(9)	11.3258(4)
c/Å	23.9896(9)	19.337(4)	30.6892(9)	27.6688(11)	21.2195(6)	12.089(13)	17.6188(6)
α/°	89.203(3)	102.927(5)	84.5201(18)	97.5447(19)	90	80.713(18)	90
β/°	83.210(3)	105.949(7)	82.3776(18)	99.898(2)	98.0799(15)	80.22(4)	106.1224(15)
γ/°	79.715(3)	113.742(5)	77.4770(17)	117.2468(17)	90	75.41(4)	90
Volume/Å^3	7897.6(6)	4586.9(17)	4334.1(2)	8957.7(6)	11610.4(6)	592.7(13)	3489.6(2)
Z	2	2	2	2	8	2	8
D_{calc} g/cm^3	1.328	1.261	1.281	0.981	1.212	1.408	1.166
μ/mm^{-1}	1.719	0.357	1.472	1.799	1.062	0.111	0.702
F(000)	3320.0	1845.0	1770.0	2808.0	4488.0	264.0	1312
Crystal size/mm^3	0.52 × 0.05 × 0.05	0.242 × 0.232 × 0.062	0.15 × 0.06 × 0.02	0.35 × 0.11 × 0.09	0.25 × 0.17 × 0.13	0.28×0.15×0.03	0.2x0.2x0.09
Radiation	CuKα	MoKα	CuKα	CuKα	CuKα	MoKα	MoKα
Wavelength/Å	1.54178	0.71073	1.54178	1.54178	1.54178	0.71073	0.71073
2θ/°	3.71 to 139.574	5.262 to 53.138	5.824 to 139.928	3.338 to 134.998	5.076 to 141.472	5.614 to 55.132	4.651 to 68.294
Reflections collected	28881	75488	57091	103813	69660	8726	11132
Independent reflections	28881	18997	16155	31757	10915	2736	3192
R_{int}	merged	0.1702	0.0595	0.0510	0.0527	0.0445	0.0347
R_{sigma}	0.2389	0.1623	0.0640	0.0511	0.0337	0.0490	0.0327
Restraints	773	769	798	352	177	0	53
Parameters	2274	1372	1298	1865	763	166	248
GooF	1.022	1.018	1.162	1.084	1.128	1.033	1.053
R_1 [I> 2σ (I)]	0.1316	0.1007	0.1158	0.0667	0.0684	0.0461	0.0380
wR_2 [I> 2σ (I)]	0.3383	0.2502	0.3121	0.1958	0.1946	0.0979	0.1084
R_1 [all data]	0.2334	0.2254	0.1561	0.0837	0.0816	0.0809	0.0412
wR_2 [all data]	0.4267	0.3283	0.3495	0.2099	0.2077	0.1130	0.1118
Largest peak/e Å^{-3}	1.08	1.46	1.15	0.80	0.69	0.27	0.203
Deepest hole/e Å^{-3}	-1.28	-0.75	-0.58	-0.75	-0.76	-0.27	-0.171

Demethylation and Substitution attempts

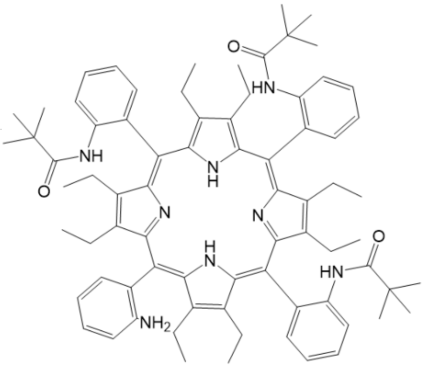

Chemical Formula: $C_{75}H_{90}N_8O_3$
Exact Mass: 1150.7136

Elemental Composition Report

Single Mass Analysis

Tolerance = 20.0 PPM / DBE: min = -1.5, max = 200.0
Element prediction: Off
Number of isotope peaks used for i-FIT = 5

Monoisotopic Mass, Odd and Even Electron Ions
19 formula(e) evaluated with 1 results within limits (up to 10 best isotopic matches for each mass)
Elements Used:
C: 0-75 H: 0-91 N: 0-8 O: 0-3

Karolis Norvaisa (MSe), KN71B_crude
Q-TOF20180409MF003 55 (1.185) AM (Cen,6, 80.00, Ht,10000.0,1570.68,0.70); Sm (SG, 2x3.00); Sb (15,10.00); Cm (8:97-(53:56+78:80)) TOF MS LD+ 1.43e+003

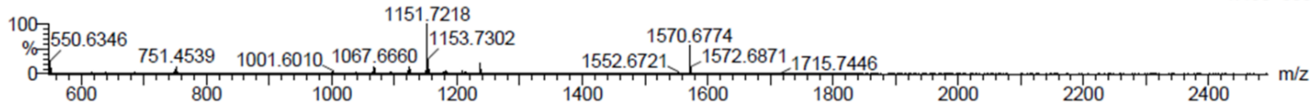

Minimum:				-1.5			
Maximum:		5.0	20.0	200.0			
Mass	Calc. Mass	mDa	PPM	DBE	i-FIT	i-FIT (Norm)	Formula
1151.7218	1151.7214	0.4	0.3	34.5	57.7	0.0	C75 H91 N8 O3

Figure S60. HRMS (MALDI) of the reaction mixture in attempt to produce **2**

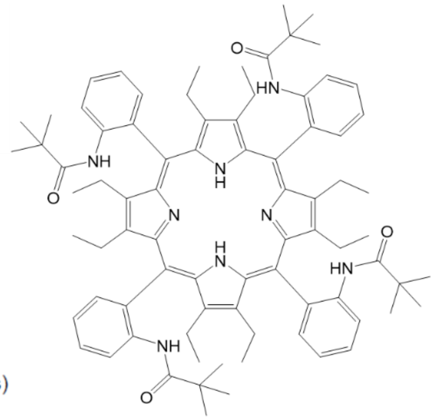

Chemical Formula: $C_{80}H_{98}N_8O_4$
Exact Mass: 1234.7711

Elemental Composition Report

Single Mass Analysis

Tolerance = 5.0 PPM / DBE: min = -1.5, max = 200.0
Element prediction: Off
Number of isotope peaks used for i-FIT = 5

Monoisotopic Mass, Odd and Even Electron Ions
357 formula(e) evaluated with 1 results within limits (up to 10 best isotopic matches for each mass)
Elements Used:
C: 0-80 H: 0-99 N: 0-8 O: 0-4 Ir: 0-1

Karolis Norvaisa (MSe), KN71B_col
Q-TOF20180413MF009 36 (0.667) AM (Cen,6, 80.00, Ht,10000.0,1570.68,0.70); Sm (SG, 2x3.00); Sb (15,10.00); Cm (7:69) TOF MS LD+ 1.04e+003

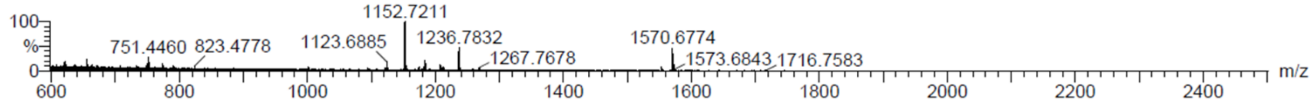

Minimum:				-1.5			
Maximum:		5.0	5.0	200.0			
Mass	Calc. Mass	mDa	PPM	DBE	i-FIT	i-FIT (Norm)	Formula
1235.7780	1235.7789	-0.9	-0.7	35.5	87.4	0.0	C80 H99 N8 O4

Figure S61. HRMS (MALDI) of the reaction mixture in second attempt to produce **2**

Table S2. Demethylation attempts of nonplanar porphyrin **3**.

Entry	Temperature	Time	Solvent	Demethylating agent	Eq.	Number of methyl groups cleaved*
1	20 °C	18 h	DCM	BBr_3	24	4
2	50 °C (Microwave)	10 min	$CHCl_3$	TMSI	40	0
3	50 °C (Microwave)	20 min	$CHCl_3$	TMSI	40	0
4	50 °C (Microwave)	30 min	$CHCl_3$	TMSI	40	0
5	50 °C	24 h	$CHCl_3$	TMSI	40	0
6	20 °C	18 h	DCM	BBr_3	76	6, 5, 4
7	20 °C	48 h	DCM	BBr_3	76	6, 5, 4 + side prod.
8	20 °C	96 h	DCM	BBr_3	76	7, 6, 5 + side prod.
9	20 °C	8 d	DCM	BBr_3	76	Majority of side prod.
10	20 °C	18 h	*o*-Dichlorobenzene	BBr_3	16	4, 5
11	110 °C	2 h	*o*-Dichlorobenzene	BBr_3	16	6
12	110 °C	6.5 h	*o*-Dichlorobenzene	BBr_3	16	8 (trace), 7, 6
13	110 °C (Microwave)	10 min	*o*-Dichlorobenzene	BBr_3	25	4
14	110 °C (Microwave)	30 min	*o*-Dichlorobenzene	BBr_3	25	6, 5, 4
15	110 °C (Microwave)	60 min	*o*-Dichlorobenzene	BBr_3	25	6
16	110 °C	3.5 h	*o*-Dichlorobenzene	BBr_3	75	6
17	20 °C<br>20 °C	18h<br>48h	DCM<br>DCM	BBr_3<br>BBr_3	43<br>43	5, 4<br>7, 6

* Indicated by mass spectrometry

Stability of Individual Atropisomers

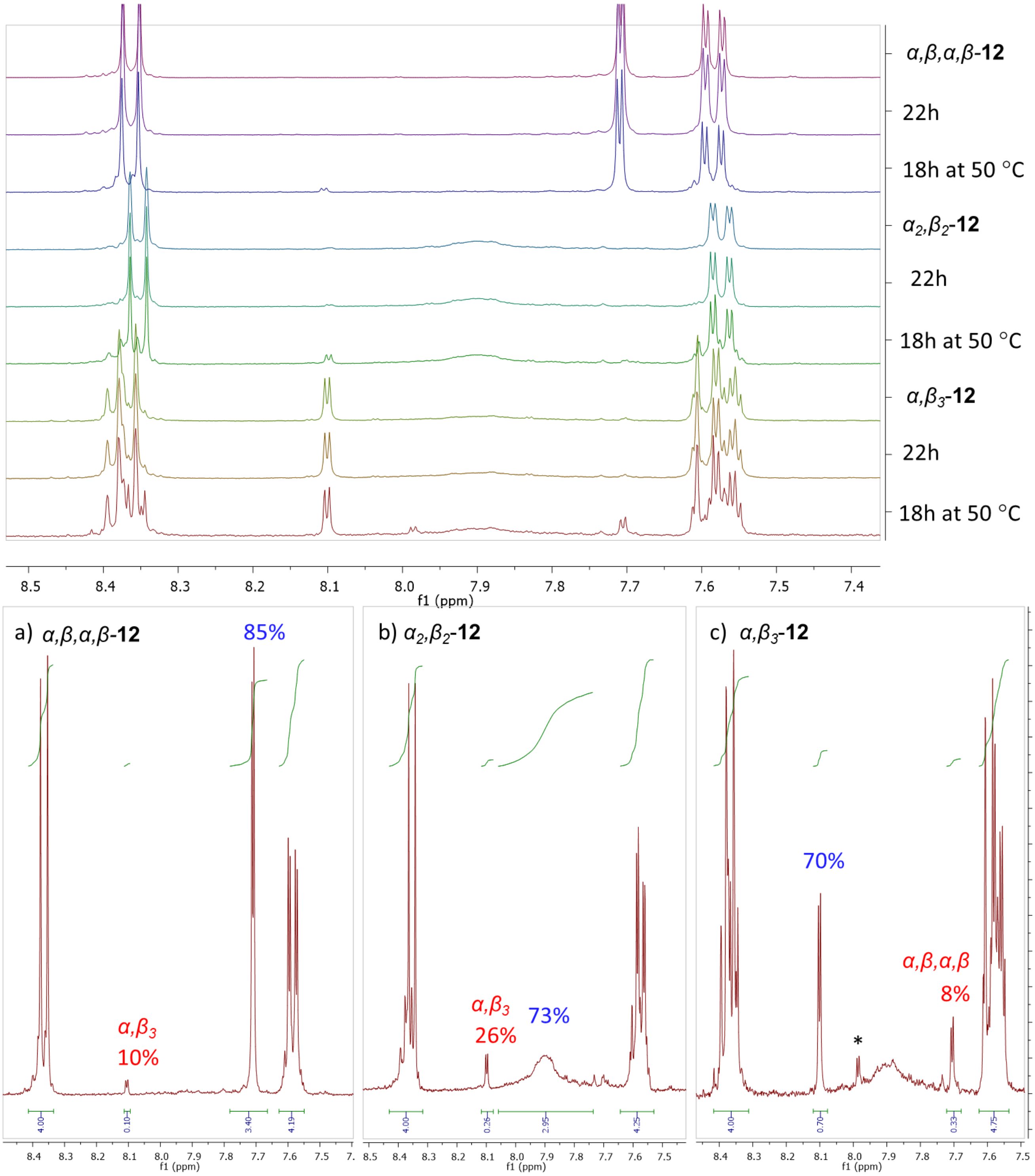

Figure S62. Stability evaluation of *α,β,α,β*-**12**, *$\alpha_2\beta_2$*-**12**, and *$\alpha_3\beta$*-**12**, by ^{1}H NMR recorded in $CDCl_3$. Top: over a period of 22h at 25 °C and 18h at 50°C (note, no spectroscopic changes was observed at room 25 °C). Bottom: enlargement of the aromatic region signals of the samples a) *α,β,α,β*-**12**, b) *$\alpha_2\beta_2$*-**12**, and c) *$\alpha_3\beta$*-**12** recorded after 18h at 50°C. In blue, the percentage value of the integrated characteristic signal corresponding to the domain atropisomer. In red, the predominant observable signal corresponding to the equilibrated atropisomer. Note, due to the very broad profile of *$\alpha_2\beta_2$*-**12** characteristic signal, the tracing the exact atropisomeric composition can be unattainable. Highlighted with star symbol is unidentified signal that could potentially correspond to the formation of *α_4*-**12**.

References

[1] a) J. S. Lindsey, I. C. Schreiman, H. C. Hsu, P. C. Kearney, A. M. Marguerettaz, *J. Org. Chem.* **1987**, *52*, 827–836; b) J. L. Sessler, A. Mozattari, M. R. Johnson, *Org. Synth.* **1998**, *70*, 68–74.

[2] K. Norvaiša, K. J. Flanagan, D. Gibbons, M. O. Senge, *Angew. Chem.* **2019**, *131*, 16705–16709; *Angew. Chem. Int. Ed.* **2019**, *58*, 16553–16557.

[3] APEX3, Version 2016.9-0, Bruker AXS, Inc., Madison, WI, **2016**.

[4] SADABS, Version 2016/2, Bruker AXS, Inc., Madison, WI, **2014**.

[5] W. Jentzen, X.-Z. Song, J. A. Shelnutt, *J. Phys. Chem. B* **1997**, *101*, 1684–1699; C. J. Kingsbury, M. O. Senge, *Coord. Chem. Rev.* **2021**, *431*, 213760.

[6] L. Sun and J. A. Shelnutt, NSDGUI (Version 1.3 Alpha version), Sandia National Laboratory, Albuquerque, USA., **2000–2001**.

[7] H. Hope, *Prog. Inorg. Chem.* **2007**, *41*, 1–19.

[8] a) O. V. Dolomanov, L. J. Bourhis, R. J. Gildea, J. A. K. Howard, H. Puschmann, *J. Appl. Cryst.* **2009**, *42*, 339–341; b) G. Sheldrick, *Acta Crystallogr.* **2015**, *A71*, 3–8.
